# The Efficacy of Cognitive Intervention in Mild Cognitive Impairment (MCI): a Meta-Analysis of Outcomes on Neuropsychological Measures

**DOI:** 10.1007/s11065-017-9363-3

**Published:** 2017-12-27

**Authors:** Dale S. Sherman, Justin Mauser, Miriam Nuno, Dean Sherzai

**Affiliations:** 1 Cedars-Sinai Medical Center, 444 S. San Vicente Blvd, Suite 103, Los Angeles, CA 90048 USA; 2 University of Southern California, Los Angeles, CA USA; 3 Virginia Commonwealth University School of Medicine, Richmond, VA USA; 4 University of California, Davis, Davis, CA USA; 5 Loma Linda University Health, 11370 Anderson Street B100, Loma Linda, CA 92354 USA

**Keywords:** Mild cognitive impairment (MCI): Cognitive interventions, Cognitive training, Cognitive strategies, Cognitive rehabilitation, Treatment efficacy, Neuropsychological outcomes, Meta-analysis

## Abstract

Cognitive training in MCI may stimulate pre-existing neural reserves or recruit neural circuitry as “compensatory scaffolding” prompting neuroplastic reorganization to meet task demands (Reuter-Lorenz & Park, 2014). However, existing systematic reviews and meta-analytic studies exploring the benefits of cognitive interventions in MCI have been mixed. An updated examination regarding the efficacy of cognitive intervention in MCI is needed given improvements in adherence to MCI diagnostic criteria in subject selection, better defined interventions and strategies applied, increased use of neuropsychological measures pre- and post-intervention, as well as identification of moderator variables which may influence treatment. As such, this meta-analytic review was conducted to examine the efficacy of cognitive intervention in individuals diagnosed with mild cognitive impairment (MCI) versus MCI controls based on performance of neuropsychological outcome measures in randomized controlled trials (RCT). RCT studies published from January 1995 to June 2017 were obtained through source databases of MEDLINE-R, PubMed, Healthstar, Global Health, PSYCH-INFO, and Health and Psychological Instruments using search parameters for MCI diagnostic category (mild cognitive impairment, MCI, pre-Alzheimer’s disease, early cognitive decline, early onset Alzheimer’s disease, and preclinical Alzheimer’s disease) and the intervention or training conducted (intervention, training, stimulation, rehabilitation, or treatment). Other inclusion and exclusion criteria included subject selection based on established MCI criteria, RCT design in an outpatient setting, MCI controls (active or passive), and outcomes based on objective neuropsychological measures. From the 1199 abstracts identified, 26 articles met inclusion criteria for the meta-analyses completed across eleven (11) countries; 92.31% of which have been published within the past 7 years. A series of meta-analyses were performed to examine the effects of cognitive intervention by cognitive domain, type of training, and intervention content (cognitive domain targeted). We found significant, moderate effects for multicomponent training (Hedges’ *g* observed = 0.398; CI [0.164, 0.631]; *Z* = 3.337; *p* = 0.001; *Q* = 55.511; *df* = 15; *p* = 0.000; *I* ^*2*^ = 72.978%; *τ* ^*2*^ = 0.146) as well as multidomain-focused strategies (Hedges’ *g* = 0.230; 95% CI [0.108, 0.352]; Z = 3.692; *p*  < 0.001; *Q* = 12.713; *df* = 12; *p* = 0.390; *I* ^*2*^ = 5.612; *τ* ^*2*^ = 0.003). The effects for other interventions explored by cognitive domain, training type, or intervention content were indeterminate due to concerns for heterogeneity, bias, and small cell sizes. In addition, subgroup and meta-regression analyses were conducted with the moderators of MCI category, mode of intervention, training type, intervention content, program duration (total hours), type of control group (active or passive), post-intervention follow-up assessment period, and control for repeat administration. We found significant overall effects for intervention content with memory focused interventions appearing to be more effective than multidomain approaches. There was no evidence of an influence on outcomes for the other covariates examined. Overall, these findings suggest individuals with MCI who received multicomponent training or interventions targeting multiple domains (including lifestyle changes) were apt to display an improvement on outcome measures of cognition post-intervention. As such, multicomponent and multidomain forms of intervention may prompt recruitment of alternate neural processes as well as support primary networks to meet task demands simultaneously. In addition, interventions with memory and multidomain forms of content appear to be particularly helpful, with memory-based approaches possibly being more effective than multidomain methods. Other factors, such as program duration, appear to have less of an influence on intervention outcomes. Given this, although the creation of new primary network paths appears strained in MCI, interventions with memory-based or multidomain forms of content may facilitate partial activation of compensatory scaffolding and neuroplastic reorganization. The positive benefit of memory-based strategies may also reflect transfer effects indicative of compensatory network activation and the multiple-pathways involved in memory processes. Limitations of this review are similar to other meta-analysis in MCI, including a modest number studies, small sample sizes, multiple forms of interventions and types of training applied (some overlapping), and, while greatly improved in our view, a large diversity of instruments used to measure outcome. This is apt to have contributed to the presence of heterogeneity and publication bias precluding a more definitive determination of the outcomes observed.

**Electronic supplementary material:**

The online version of this article (10.1007/s11065-017-9363-3) contains supplementary material, which is available to authorized users.

The cognitive deficits observed in mild cognitive impairment (MCI) signal abnormal changes in neural structure and function representative of possible prodromal markers of Alzheimer’s disease (AD) or other significant neurodegenerative disorder (Albert et al. 2011; Petersen et al. 2001, 2009; Saunders and Summers 2011). These deficits exceed the age-related changes in cognitive efficiency, attention, memory, and executive functions anticipated at the fifth to sixth decade of life and may progressively accelerate into more significant cognitive declines by the seventh to eighth decades (Cabeza et al. 2017; Rog and Fink 2013; Salthouse 1996, 2011; Schaie and Willis 2010). As there are no effective medical or pharmacological intervention for the treatment of MCI or AD, other interventions such as compensatory cognitive strategies, “brain-games”, and other lifestyle changes (i.e. nutrition, exercise, etc.) are aggressively being sought to mitigate or slow illness progression (Cai and Abrahamson 2016; Curlik and Shors 2013; Kivipelto et al. 2013; Lehert et al. 2015; Simons et al. 2016; Smith et al. 2010). This may be especially important as cognitive training in MCI has been associated with increased activation in the hippocampus (Hampstead et al. 2012b; Rosen et al. 2011), right inferior parietal lobe (Belleville et al. 2011), frontoparietal network (Hampstead et al. 2011), occipito-temporal areas (Onur et al. 2016), and implicated in other processes (Barban et al. 2017; Ciarmiello et al. 2015; Maffei et al. 2017). As such, identifying the cognitive interventions effective in MCI may, in turn, aid in targeting the neural networks with greater specificity at prodromal stages to alter illness trajectory away from more serious cognitive decline (Kim and Kim 2014; Shatenstein and Bargerger-Gateau 2015; Sitzer et al. 2006).

In an effort to distill the mechanisms associated with age-related changes and pathognomonic processes across the lifespan, The Scaffolding Theory of Aging and Cognition – Revised (STAC-r) suggests cognitive training may activate compensatory neural processes or “scaffolding” to provide computational support to primary networks or newly established task networks when new skills are acquired (Reuter-Lorenz and Lustig 2017; Reuter-Lorenz and Park 2014). It has also been suggested that training may activate pre-existing cognitive reserves (Stern 2012; Wirth et al. 2014) or prompt hemispheric recruitment to meet processing demands (Hemispheric Asymmetry Reduction in Older Age [HAROLD], Cabeza 2002). The manner in which neural mechanisms are utilized may shift across the lifespan as older adults demonstrate age-related decreases in occipito-temporal activity coupled with increases in prefrontal cortex processing (Posterior-Anterior-Shift with Aging [PASA], Davis et al. 2008). Compensatory mechanisms may, however, reach a ceiling and become ineffective under high load or high demand circumstances (Compensation Related Utilization of Neural Circuits Hypothesis [CRUNCH]; Reuter-Lorenz and Cappel 2008) or become compromised by neuropathological processes. Cognitive training may stimulate pre-existing neural reserves or recruit neural circuitry as “compensatory scaffolding” prompting neuroplastic reorganization to meet task demands, in the context of adaptive factors and divergent trajectories of decline (Hong et al. 2015; van Paasschen et al. 2009).

To this end, multiple types of interventions have been employed in MCI including restorative training, compensatory-based strategies (Bahar-Fuchs et al. 2013; Gates and Valenzuela 2010; Kinsella et al. 2009; Martin et al. 2011; Simon et al. 2012), cognitive stimulation and multicomponent or multimodal forms of intervention. Please see Table S1 in the supplementary material for additional information regarding terms and definitions. However, existing reviews and prior meta-analyses have reported varying findings concerning the benefits of cognitive training. Several reviews report there to be a benefit from cognitive strategies (Coyle et al. 2015; Faucounau et al. 2010; Hill et al. 2017; Jean et al. 2010b; Li et al. 2011; Reijnders et al. 2013; Simon et al. 2012) and other analyses have found little or no advantage (Belleville 2008; Gates et al. 2011; Huckans et al. 2013; Kurz et al. 2011; Martin et al. 2011; Stott and Spector 2011; Vidovich and Almeida 2011; Zehnder et al. 2009).

While we acknowledge and appreciate these prior reviews have been conducted, to the best of our knowledge there have been no meta-analyses which examined cognitive interventions in randomized clinical trials (RCT) across neuropsychological domains exclusively in the MCI population. An updated examination of cognitive interventions is needed given improvements in the interventions and strategies applied, increased use of neuropsychological measures pre- and post-intervention, as well as identification of moderating variables which may influence intervention outcomes (e.g. MCI diagnosis, duration of training, etc.). There has been an increased use of cognitive interventions, generally (Craik et al. 2007; Mahncke et al. 2006; Purath et al. 2014; Stuss et al. 2007; Tardif and Simard 2011; Uchida and Kawashima 2008; Willis and Caskie 2013), as well as proliferation of evidence connecting training to neural substrates and neuroplastic processes (Cabeza et al. 2017; Park and Festini 2017; Reuter-Lorenz and Lustig 2017; Toepper 2017). Of particular significance has been the refinement of diagnostic criteria used to define MCI to recruit subjects and delineate treatment groups (Albert et al. 2011; Petersen et al. 2001, 2009; Winbald et al. 2004). This refinement has resulted in a better selection process and, secondarily, led to an increase in the quality of outcomes observed. However, it has taken time for these changes to find their way into published clinical trials.

A review of current works is also needed due to broadened use of neuropsychological instruments to obtain pre-intervention baseline scores and post-treatment outcomes. Improved technical precision through the use of more robust neuropsychological outcome measures has improved our understanding of the effectiveness of interventions applied (Ellis et al. 2009, 2010; Ibanez et al. 2014; Mitchell 2009) and aided with control of potential confounds due to test-retest and repeated measure experimental design. In addition, while multiple neural centers may be involved when completing test instruments (Matias-Guiu et al. 2017), objective neuropsychological measures have been shown to be sensitive to different stages of illness in neurodegenerative disorder increasing their potential role and discriminability in study execution (Han et al. 2017).

Given these changes and advancements, the present meta-analysis was conducted to examine the efficacy of cognitive interventions on neuropsychological test performance in individuals diagnosed with MCI versus MCI controls conducted in RCTs. We explored the strategies used, in both general intervention approaches and specific forms of cognitive training, in an effort to distill the cognitive tasks effective in MCI. We sought to determine (i) what were the changes in cognition from baseline to outcome after the intervention was applied (ii) what were the common characteristics of interventions found to be effective across studies, (iii) what are specific interventions that may be of benefit to individuals diagnosed with MCI in the clinical setting, (iv) what are the key structural factors needed to set-up an effective MCI intervention program (e.g. duration, frequency, homework, etc.), and (v) what inferences may be made regarding interventions applied and the neural processes involved in MCI?

Provisionally, based on STAC-r and neurocognitive models, we anticipated three possible data-patterns associated with cognitive training could emerge (i) primary network engagement, (ii) compensatory scaffolding activation, or (iii) loss of measurable response. More specifically, evidence of primary network engagement would be suggested by moderate – large effect sizes on domain-specific outcomes after domain-targeted training (direct effects). In this scenario, training would facilitate primary network engagement by creating new primary network paths (scaffolding) to meet task demands without recruiting alternate networks. We would expect interventions which are challenging, novel, and deeply engaging to result in a more ‘youthful’ performance due to greater efficiency and less reliance on compensatory processes (Reuter-Lorenz and Park 2014; Vermeij et al. 2017). Restorative strategies which target specific cognitive functions may facilitate this process as restorative interventions aim to return deficits to premorbid levels (i.e. errorless learning), although other forms of training may also improve efficiency to a lesser degree (i.e. compensatory strategies).

Primary network engagement would also be suggested by moderate to large effect sizes with multicomponent or multidomain interventions. In this instance, training may act on primary networks and complementary processes simultaneously requiring integration of complex skillsets, including lifestyle changes, mitigating structural and functional declines by enhancing processing in specific centers and decreasing the neural burden on other areas (Barban et al. 2017; Hosseini et al. 2014; Suo et al. 2016). Multidomain approaches may also target multiple neural regions for a more enriched and complex neural challenge (Ballesteros et al. 2015; Li et al. 2017). Moreover, multidomain approaches may offer greater utility as most cognitive skills are not unitary, single-domain processes but involve interrelated cognitive functions across several areas which, after training, prompt inclusion of additional networks (Belleville et al. 2011). As such, small to moderate effects may also be demonstrated in non-targeted domains (transfer effects). In addition, cognitively stimulating activities may aid in engaging cognitive reserves to slow decline or reduce the risk of greater pathology (Ciarmiello et al. 2015; De Marco et al. 2016; Herholz et al. 2013; Stern 2012). There may be protective factors or moderator effects on outcome measures such as level of education, occupation, and intelligence as well (Hall et al. 2009).

A second data-pattern, compensatory activation, would be suggested by small effects sizes associated with training in targeted domains, no effects on non-targeted domains (absence of transfer effects), and small to moderate effect sizes associated with multicomponent or multidomain forms of intervention. In this instance, training would recruit alternate networks to meet task demands. However, smaller effect sizes would be anticipated as primary networks would be unable to manage the load due to loss of functionality, decreased efficiency, and an increase in dedifferentiation (decrease in specialization). As such, multimodal forms of intervention may be more efficacious as several strategies can support primary networks and recruited networks simultaneously.

Finally, as a third data-pattern, the absence of any training effect would suggest individuals with MCI have lost sufficient neurocognitive plasticity to engage primary networks and are unable to recruit alternate compensatory mechanisms to meet task demands.

## Methods

The search process and meta-analyses performed followed guidelines outlined by the Preferred Reporting Items for Systematic Reviews and Meta-Analysis (PRISMA; Moher et al. 2009) using a PICOS approach (Participants, Interventions, Control Outcomes, and Study Design). Pursuant to the recommendation by Gates and March (2016), the PRISMA checklist items are addressed in the sections below. Please see Table S2 in the supplemental materials for the PRIMSA items cross-referenced by page.

### Protocol Registration

The research methodology and protocol for this meta-analysis was not registered prior to conducting the review. All methods and procedures regarding the search and analysis are described in the paper with additional information provided in the supplemental materials.

### Study Eligibility – Inclusion & Exclusion Criteria

This review focused on studies published from January 1995 to June 2017 which (i) selected subjects based on established-MCI criteria (Albert et al. 2011; Petersen et al. 2009; Winbald et al. 2004), (ii) performed a RCT in an outpatient setting, (iii) compared cognitive training versus controls (active or passive), and (iv) reported outcomes based on objective neuropsychological measures. The start date of January 1995 was chosen as beginning point as a cursory search for studies prior to this time resulted in no relevant works and it was believed studies published prior to 1995 would not have recruited subjects according to current diagnostic criteria. The definition used for cognitive intervention was any strategy or skill which sought to improve mental processes of attention and concentration, speed of information processing, memory, or executive functions, similar to the guidelines offered by Gates and Valenzuela (2010). We did not define cognitive intervention solely in classical forms such as restorative, compensatory, etcetera, in an effort to obtain a broadly inclusive dataset, although we also recorded the strategy type used according to cognitive training categorization suggested by Simon et al. (2012) to examine specific methods of training. For the purposes of this review, we have adopted the following terminology (i) intervention as a broad-based idiom to refer, generally, to any effort employed, (ii) cognitive stimulation to mean nonspecific and leisure forms of activities, (iii) cognitive training to denote either compensatory or restorative forms of training, and, (iv) multicomponent forms of intervention to mean the combination of several approaches used together. Please see Table S1 for additional information regarding terms and definitions. In addition, we selected only those studies which were RCTs with a clearly defined patient sample population according to MCI criteria (Albert et al. 2011; Petersen et al. 2001; Petersen 2004, Petersen et al. 2009; Winbald et al. 2004) or analogous definition using an algorithm of 1.5 standard deviations below the mean (Vidovich et al. 2015) on established neuropsychological instruments, that is, Consortium of Established Registry for Alzheimer’s Disease (CERAD; Fillenbaum et al. 2008). Studies performed in a day-treatment, institutional, or group-residential setting were not considered due to concerns regarding the influence of non-controlled effects and other potential confounds.

### Information Sources – Databases Searched

Our review was conducted through the OVID-MEDLINE search engine using a collective of the source databases MEDLINE-R, PubMed, Healthstar, Global Health, PSYCH-INFO, and Health and Psychological Instruments. There were no other data sources, information used from informal contact, or data obtained through other methods of communication (e.g., email, conference, etc.).

### Literature Search Parameters

The primary search parameters included (i) terms representative of the MCI diagnostic category (mild cognitive impairment, MCI, pre-Alzheimer’s disease, early cognitive decline, early onset Alzheimer’s disease, and preclinical Alzheimer’s disease), (ii) a descriptor of the intervention or training conducted (intervention, training, stimulation, rehabilitation, or treatment), (iii) RCT, (iv) limit to “1995-Current”, and (v) limit to human. The specific Boolean search strategy statements and result counts are provided in the supplemental materials as Table S3.

### Study Screening & Selection

**Fig. 1:**
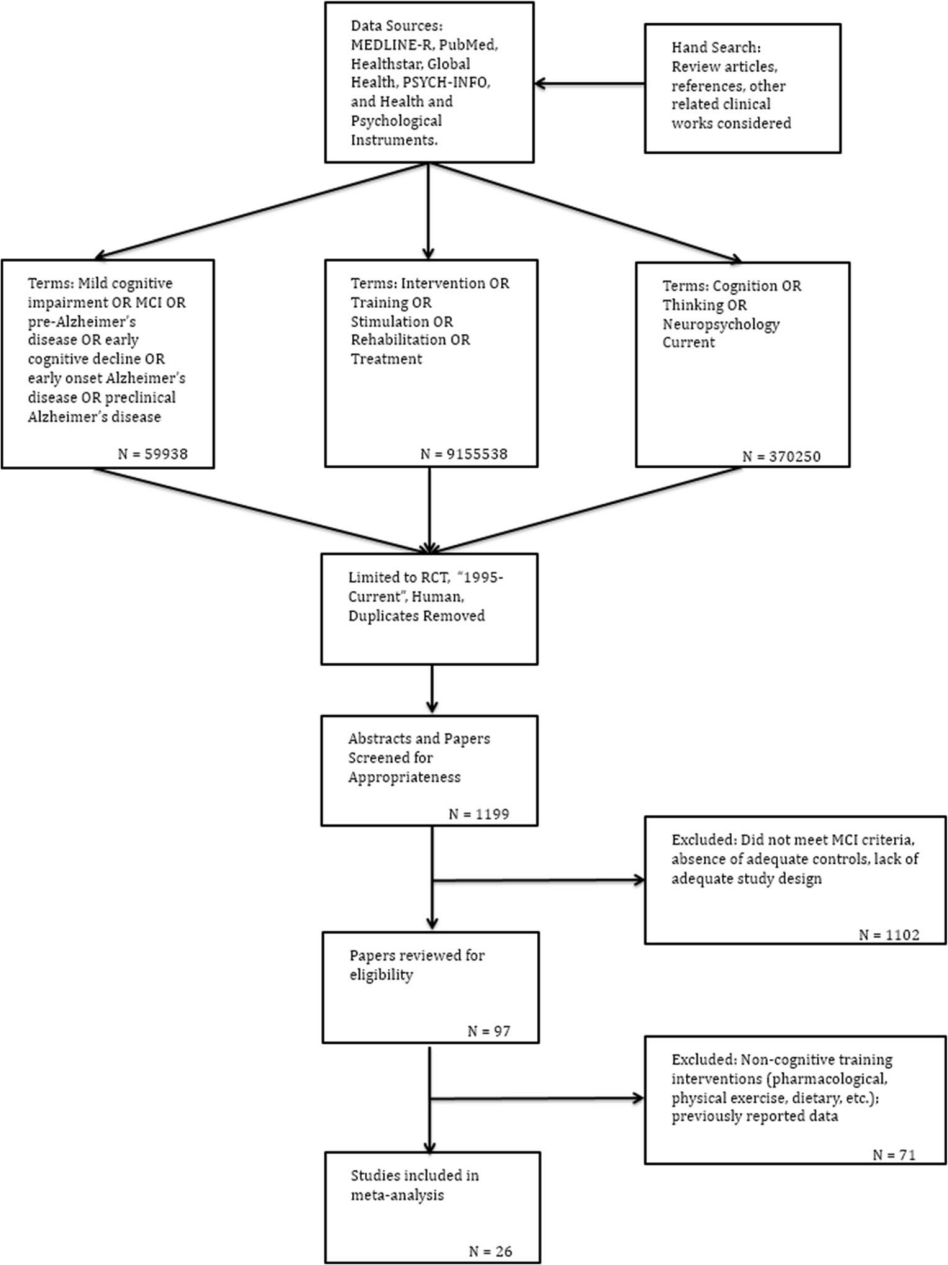
Literature review flow diagram

The process by which studies were identified, screened, considered for eligibility and included is illustrated in Fig. 1. The first two authors (DSS & JM) conducted the literature search and screened potential studies following the search criteria described above. This was done independently in two search waves with periodic confirmation of the eligibility criteria and progress made in study selection.

### Data Collection Process and Data Items

The effects of training were evaluated through subjects’ performance on outcome measures, defined as scores obtained on neuropsychological test instruments. Cognitive domains were delineated into generally accepted pre-established categories of mental status and general cognition, working memory or attention, speed of information processing, language, visual-spatial ability, memory (verbal and non-verbal), and executive functions. Multiple test instruments and test versions were administered specific to study location sensitive to language and culture of origin. We defined mental status and general cognition in broad terms and adopted scores from abbreviated measures as well as summary scores from larger instruments. Measures assessing learning and memory were grouped into (i) general memory overall, then further subdivided into (ii) verbal and (iii) non-verbal categories. Verbal memory was further split into (i) list learning and (ii) story recall, while non-verbal memory measures were not subdivided. A summary of neuropsychological instruments listed by cognitive domain are presented in the supplemental materials as Table S5. Variables of IQ, age, education, and treatment duration (hours) were extracted and coded as continuous variables. In addition, mode of training, type of training, domain targeted (intervention content), type of control, MCI type, period to follow-up assessment, and control for repeat administration were delineated as categorical variables and dummy coded.

### Risk of Bias – Individual Studies

The risk of bias in individual studies and quality of each study was independently weighted by two of the authors (DSS & JM) using the NIH Quality of Assessment of Controlled Intervention Studies Scale (2017). The NIH instrument is a 14-item scale with which publish works may be rated in terms of randomization, blinded status of the participants, drop-out rates, etc. (NIH; www.nhlbi.nih.gov). Studies were not explored for determination of bias secondary to funding sources or other form of bias such as institution or affiliation.

### Summary Measures

To quantify the possible benefit of training, we sought to determine the difference in post-training scores between MCI intervention groups versus MCI controls (waitlisted, non-trained or active control subjects). As such, means and standard deviations from test instruments were extracted from each study as primary summary measures to calculate summary statistics, effect sizes (weighted and un-weighted), and 95% confidence intervals (CI). When means and standard deviations were not reported directly, *p*-values, *t*-values, *F*-values, or confidence interval data were extracted to calculate the mean and standard deviation statistics for intervention and control groups. Data from outcome measures were extracted from the time-point closest to the conclusion of training, if more than one wave of data collection was conducted post-training. Differences between means were calculated according to Hedges’ *g* metric. The overall summary effect size, forest plots as well as individual effect sizes within specific cognitive domains were examined. The values used to interpret effect size were in keeping with established guidelines such that a small effect size was defined as 0.20 or small (range 0–0.20); moderate = 0.50 (range 0.30–0.70); and large = 0.80 (range > = 0.80; Cohen 1988; Durlack 2009). Given the likely heterogeneity resulting from variability of training approaches, range of outcome measures, and differing methodological procedures across studies, a random-effects model was assumed for all analyses (DerSimonian and Laird 1986). A prediction interval was calculated at 95% confidence to approximate the range of effect which might be anticipated under similar intervention conditions with the same outcome measure(s) reported based on the procedure recommended by Borenstein et al. (2016) and performed with a Microsoft excel spreadsheet graciously provided by the authors.

### Synthesis of Results & Measures of Inconsistency

Means and standard deviations from neuropsychological measures for each study were entered for analysis. For studies with more than one outcome measure, a combined outcome, or ‘synthetic variable’, was computed through combining all test results reported from the study to produce a single mean difference, in accordance with the procedure recommended by Borenstein et al. (2009). As such, each study was represented by one score and contributed only one effect size in the meta-analysis regardless of the number of outcomes administered in the study. This approach was taken to restrict artificial inflation, potential interdependence of observations, and to avoid error due to redundancy. To ensure this, we examined summary effects and measures of dispersion by conducting sensitivity analyses (M. Borenstein, personal communication, September 2017) for select meta-analyses assuming various levels of correlation between outcome measures (0.0, 0.20, 0.40, 0.60, 0.80, and 1.0). Meta-analyses were conducted with the Comprehensive Meta-Analysis 3.3.070 software (CMA) with some adjunct exploratory analysis performed with Meta-Easy Add-In for Microsoft Excel Program (Kontopantelis and Reeves 2009; Kontopantelis and Reeves 2010).

Meta-analyses were also run by cognitive domain as this was viewed to be more consistent with the construct validity and scope of outcome instruments administered (Demakis, 2006). As such, we examined summary effects by (i) cognitive domain (irrespective of intervention type), (ii) type of cognitive training, as well as (iii) the focus of interventions on specific outcomes associated with the targeted domain (i.e. effects of memory training specifically on memory measures). A minimum of five studies was used as criterion for analysis due to concerns for over-interpretation. While a minimum of two studies may be used as inclusion criteria and may be appropriate for some meta-analyses, particularly when outcome data is homogeneous or very specific (Valentine et al. 2010), we limited our meta-analysis to five studies given the range of outcome measures administered and due to cautions performing meta-analyses with less than five studies (Borenstein et al. 2009). We also tested significance and calculated confidence intervals for Hedges’ *g* overall effect for meta-analytic trials of twenty or less (<= 20) using the Hartung-Knapp-Sidik-Jonkman (HKSJ) method as this has been demonstrated to yield more adequate error rates, especially when the number of studies is small (IntHout et al. 2014).

With respect to verbal and non-verbal memory, we limited data to the delay aspect of outcome measures omitting learning trials, trial totals, immediate recall indices, and measures associated with prospective memory. Because several studies reported data for verbal and non-verbal recall as indices or composite scores, we ran meta-analyses for (i) all memory measures combined, as well as (ii) verbal memory and (iii) non-verbal memory subdomains separately. Regarding verbal memory in particular, scores from list recall and story recall measures were examined together to maximize data analysis. With respect to mental status and general cognition, we examined the effect of interventions on summary measures as subjects and patients are routinely evaluated with these screening-tests and general instruments (i.e. MMSE, MOCA, DRS, ADAS-Cog, RBANS total, etc.). When examining the effects of interventions directed at a specific domain, we categorized studies based on the intervention and content described and limited the meta-analysis to outcomes associated with that domain.

### Risk of Bias - Publication Bias

To examine the potential for publication bias, we performed several analyses including (i) visual inspection of funnel plot symmetry, plotting the standard difference in means to standard error (Rosenthal 1979; Sterne et al. 2000, 2011), (ii) calculation of Egger’s regression intercept (Egger et al. 1997), and (iii) Duval and Tweedie’s trim-and-fill method for imputed estimates and adjusted values (Duval and Tweedie 2000). The test for heterogeneity was based on Cochran’s *Q* and τ^2^ statistic. The *I* ^*2*^ value was also calculated, although this was reported for descriptive purposes only as this would not be regarded to be an adequate measure of inconsistency and has limited generalizability (Borenstein et al. 2016). Values of *I* ^*2*^ were characterized as small when *I* ^*2*^ = 25% (<= 25%), moderate for *I* ^*2*^ = 50% (26–74%), and large as *I* ^*2*^ = 75% (> = 75%; Higgins et al. 2003). No study was removed when examining potential contributions to heterogeneity nor did we exclude and re-run a meta-analysis as this was regarded as altering the study eligibility criteria previously established for our search (Higgins 2008).

### Additional Analyses – Moderator Variables & Meta-Regression

A subgroup meta-analysis and hierarchical meta-regression were conducted to examine the possible influence of moderator variables on outcome measures as well as contributions to heterogeneity (Borenstein et al. 2009; Borenstein and Higgins 2013; Rutter and Gatsonis 2001; Sedgewick 2013; Thompson and Higgins 2002). This was performed with MCI diagnosis, type of training, mode of treatment (group, individual, computer), primary focus of the intervention (memory, multicomponent, etc.), type of control group (passive or active), time of post-intervention follow-up assessment, and adjustment for repeat administration. These were evaluated using categorical variables dummy coded in the manner outlined above. All studies and outcome measures were included in the analysis, with the exception of outcomes which would have introduced duplicate data (i.e. MOCA – List Recall, MMSE – List Recall, MOCA – Clock Drawing, etc.).

A hierarchical (incremental) meta-regression was also used to explore the incremental contribution of moderators in a systematic manner. Given concerns regarding small number of studies in meta-regression, we limited the number of moderators used to a ratio of one covariate for every ten studies (Borenstein et al. 2009). With this criterion, the number of covariates was limited to two and entered in the order of duration of training and type of cognitive training. We also conducted an additional post hoc meta-regression in accordance with the recommendation of Fu et al. (2010) in which six to ten studies for continuous variables and four studies for categorical variables may be acceptable when effect sizes are moderate or large.

## Results

### Studies Selected

The inclusion and exclusion criteria, search strategy, and paper selection process we followed resulted in a set of 1199 studies for consideration. From this, 1102 were removed for various reasons, including, not meeting MCI criteria, containing mixed diagnostic categories (i.e. MCI and mild AD), not an RCT, lacking adequate controls, improper design or did not have the requisite study arms, did not contain data or not reporting data in a format suitable for extraction, not describing the key elements necessary for consideration in sufficiently clear manner for inclusion (i.e. did not explicitly state MCI definition for subject selection), or similar complication**.** An additional 71 studies were excluded as the interventions used would not be considered to be cognitive in nature (i.e. pharmaceutical, physical exercise, dietary, etc.). Please see Fig. 1 for a flow diagram of the paper selection process and Table S3 for the Boolean search strategy, terms and results of each step taken.

### Study Characteristics

In all, a total of 26 articles met inclusion criteria for meta-analyses. Please see Table 1 for a detailed overview of all studies examined. The total number of studies meeting criteria was considered acceptable and sufficient to proceed (*k* = 26) as this was well above the median number of six studies per meta-analysis reported in a well-known repository of metanalytic reviews, the Cochrane Database of Systematic Reviews, and exceeded 90% of the analyses (*k* = 10) conducted in the mental health and behavioral condition category (Davey et al. 2011). The majority of studies included in our cohort were published recently. 92% of the studies included (24/26) were published within the past 7 years (2010–2017) and 8% of the studies (2/26) were published prior to 2010. The total number of participants who received training was 876 (pooled) with a mean of 33.69 subjects per study (SD = 35.07; Range 6–145). The diagnosis of study participants was comprised of amnestic MCI – single domain (19.23%), amnestic – multiple domain MCI (26.92%) as well as all MCI subtypes combined (53.85%). Samples were drawn from one multicenter group of nations (Italy, Greece, Norway, & Spain) and eleven individual countries, Argentina (*n* = 1), Australia (*n* = 5), Brazil (n = 1), Canada (*n* = 2), France (n = 1), Germany (n = 2), Greece (n = 1), Hong Kong (n = 1), Italy (*n* = 4), Korea (n = 1), and the United States (*n* = 6). A summary of the age, education, mental status, number of participants, treatment duration, and span of involvement is presented in Table S6.

**Table 1:** Summary of findings: cognitive intervention impact on participants with mild cognitive impairment (MCI)

	Author	Study design	Sample size & age means	Intervention and Duration	Outcome measures	Results	General conclusions	NIH quality (standard score)
1.	Balietti et al. (2016)	RCT, inactive control	*n* = 70 (MCI) Tx *n* = 37 Cntl *n* = 33 Age: $\overline{x}$ = 75.74 s = 0.716	• 10 sessions, 60 min-1×/ week (10 weeks) • Group based, multi-component cognitive training using cognitive exercises, effective aids, and mnemonic strategies (errorless learning, spaced retrieval, organization, categorization, clustering, method of loci, visual imagery, and face-name associations)	DS-FWD; CSST; AMT, phonemic fluency, semantic fluency, prose recall, word pair learning	• Cognitive training group demonstrated statistically significant increased improvements in attentive matrices (*p* = 0.010), phonemic verbal fluency (*p* = 0.018), and recall of prose passages (immediate *p* = 0.037, delayed *p* = 0.038, and total recall *p* = 0.006) • MCI controls evidenced no change from baseline scores in follow-up evaluation	• While this study was primarily focused on effect of cognitive training on platelet phospholipases A_2_ activity (tPLA_2_A), a benefit in cognition was observed in several areas of cognition • Increases were noted in AMT, phonemic fluency, and recall of prose passages • There appeared to be no benefit in attention, CSST, semantic fluency, or word pair learning	−0.713
2.	Barban et al. (2016)	RCT, single-blind, inactive control	*n* = 106 (MCI) Tx *n* = 46 Cntl *n* = 60 Age: $\overline{x}$ = 74.4 s = 5.7	• 24 sessions, 60 min session (2×/ week for 3 months) • Group based, computer administered with software designed in multicomponent cognitive exercises/training in memory, logical reasoning, orientation, language, constructional praxis, questions concerning autobiographical data (SOCIABLE)	RAVLT, ROCT, TMT A&B, Phonemic Fluency, MMSE	• Significant differences observed post-training in RAVLT scores (*p* = 0.009), Phonemic fluency (*p* < 0.001), and MMSE (p = 0.01) in the MCI group • No differences found on RCFT or TMT measures • Improvement in RAVLT memory scores maintained after a three month ‘rest’/ period of no training or booster	• There was a medium positive effect of computer-based training on verbal memory, phonemic fluency, and mental status • Training benefit was sustained on verbal memory after three months of ‘rest’ (no training) • Computer-based multi-component training demonstrated positive effects on cognition sustained at three months; verbal memory in particular • Unclear if scores are significantly different from individuals in the passive, control arm	−0.026
3.	Barnes et al. (2009)	RCT, single-blind, active controls	*n* = 47 (MCI) Tx *n* = 17 Cntl *n* = 19 Age: $\overline{x}$ = 74 s = not reported Range = 54–91	• 100 min/ day, 5 days/ week • Computer-based, at-home independent exercises using restorative training strategies targeting auditory processing speed and working memory	1° - RBANS 2° - CVLT-II, COWAT, BNT, California TMT, Design Fluency tests, Spatial Span test, GDS	• Most group differences not statistically significant (i.e. RBANS total scores ↑ 0.36 SD in the intervention group (*p* = 0.097) compared to 0.03 SD in the control group (*p* = 0.88) for a non-significant difference); however, effect sizes for measures of learning/memory favored intervention group (range: 0.16 to 0.53 SD) • Largest effect size observed for Spatial Span, scores ↑ significantly in the intervention group (*p* = 0.04) and ↓ significantly in the control group (*p* = 0.02) for a significant effect size (*p* = 0.003)	• Intensive computer-based mental activity training is feasible in elders with MCI • Training appeared to benefit spatial span ability • Trend toward benefit of training on measures of learning/ memory • Training did not appear to benefit language, visuospatial skills	−0.026
4.	Buschert et al. (2011)	RCT, single-blind, active control	*n* = 43 aMCI *n* = 27 mild AD *n* = 16 Tx *n* = 10 Cntl *n* = 12 Age: $\overline{x}$ =71.2 s = 7.0	• 11 session minimum - 20 “units”, 120 min/ week • Group-based, multicomponent cognitive intervention including memory, cognitive stimulation, face-name association, errorless learning, principles of meta-cognition, education	ADAS-cog, MMSE, TMT B, RBANS, story recall, MADRS, QoL-AD	• Significant interaction between treatment and progression for ADAS-Cog (F = 6.2, *p* = 0.02, η2 = 0.26), MMSE (F = 3.8, *p* = 0.07, η2 = 0.17), RBANS story memory (F = 3.4, *p* = 0.08, η2 = 0.16) and TMT = B (F = 3.5, p = 0.08, η2 = 0.16) • Main effects for treatment were found for MMSE (F = 8.5, *p* < 0.01, η2 = .23), RBANS story memory (F = 12.5, p < 0.01, η2 = 0.41) and RBANS Story recall (F = 9.9, p < 0.01, η2 = 0.36)	• ↑ in global cog status, and specific cog and non-cog functions • aMCI demonstrate significant change in ADAS-Cog scores • aMCI subjects demonstrated tendency toward higher attentional skills demonstrated by TMT-B • No significant effect on memory performance compared to active controls	0.661
5.	Carretti et al. (2013)	RCT, active control	n = 20 (aMCI) Tx n = 10 Cntl n = 10 Age: Tx: $\overline{x}$ = 71.8 s = 2.20 Cntl: $\overline{x}$= 70.6 s = 2.63	• 5, 90 min sessions • Individual-based, one-on-one sessions using restorative strategies which focused on working memory techniques	NPE, Vocab, CWMS, DS-FWD; DS-BWD, Dot matrix, List recall, Pattern comparison, Cattell test	• Significant effect post training in CWMS (3.8), Dot Matrix (2.3), Cattell test (0.50) vs control CWMS (0.60), Dot Matrix (2.3), Cattell test (−0.40). *P*-values for these tests where groups improved by 1 SD are <0.01, <0.05, and <0.05, respectively	• Verbal working memory training is a promising approach to sustaining memory function in aMCI • Working memory training showed transfer to some cognitive components of memory part of the core cognitive impairments responsible for MCI → AD	−0.713
6.	Fiatarone Singh et al. (2014)	RCT, double-blind, active control	*n* = 100 Tx *n* = 24 Cntl = 27 Age: $\overline{x}$ = 70.1 s = 6.7	• 4–45 min exercises (initial/ group setting); then 45 min sessions, 2 days per week for 24 weeks • CT computer-based multicomponent and multi-domain compensatory training in memory, executive functions, attention, and speed of information processing (COGPACK)	ADAS-Cog; MMSE; GP-Cog; CDR; Matrices, Similarities, TMT A & B, LM I&II; BVMT-R; SDMT; Semantic Fluency, COWAT, MARS-MF	• ADAS-Cog scores: No differences between CT and sham cognitive training • CT group demonstrated modest non-significant changes as compared to controls across cognitive domains • CT group maintained memory ability, no significant improvement or decline	• CT reported to attenuate decline in memory • There was no significant change in cognitive outcomes between CT and sham training in global cognition, executive functions, memory, or speed	2.035
7.	Finn and McDonald (2011)	RCT, single-blind, inactive control	n = 16 (MCI-SD/MD) Tx = 8 Cntl = 8 Age: Tx: $\overline{x}$= 69.00 s = 7.69 Cntl; $\overline{x}$= 76.38 s = 6.47	• 30 sessions, 11.43 weeks/ completion • Computer-based restorative training program involving attention, processing speed, visual memory, cognitive control (LUMOSITY)	1° - CANTAB, RVP A, PAL, IED 2° - MFQ, DASS-21	• Significant CANTAB tests: main effect of group - attentional set shifting, visual learning, visual working memory F (2, 14) = 0.35, 1.17, and 3.55, respectively • ↑ in visual sustained attention treatment (pre/ post difference RVPA’ = .03) compared with waitlist ctrl (pre/ post difference RVPA’ = − .06) • No significant data on self-reported memory functioning and perceptions of control over memory	• ↑ in performance on visual sustained attention compared to waitlist controls • No significant changes on other primary outcome measures • MCI patients can ↑ performance significantly when given repeated practice on computerized cognitive exercises but study did not translate to secondary measures	−1.401
8.	Finn and McDonald (2015)	RCT, inactive control	n = 24 (aMCI) Tx = 12 Cntl = 12 Age: Tx: $\overline{x}$= 72.83 s = 5.7 Cntl; $\overline{x}$= 75.08 s = 7.5	• 6 sessions over several weeks (plus one practice session). • Computer-based restorative training program using strategy of repetition-lag training	1° - VPA I & II 2° - WMS-IV – SS; D-KEFS N-S; D-KEFS N-LS; CFQ; DASS-21	• Significant effects of training observed in VPA-II (F = 4.52, *p* = 0.046) • No other significant effects demonstrated with the exception of practice effects in VPA-I	• Computer-based version of modified repetition-lag training program was associated with improvement in recall of verbal pair associates in aMCI • No evidence of transfer effects on other secondary measures	−0.713
9.	Forster et al. (2011)	RCT, single-blind, active control	n = 24(aMCI), n = 15(mild AD) Tx *n* = 9 Cntl *n* = 9 Age: $\overline{x}$=74.5 s = 8.6	• 26, ~120 min sessions (1×/week for 6 months) • Group-based multi-component cognitive interventions focused on global cognition, mood, and quality of life	ADAS-cog, MMSE, FDG PET scan	• Change in ADAS-Cog scores for aMCI demonstrate interaction treatment and progression (F = 4.7; *p* = .045; η2 = .22) • Change in MMSE scores for aMCI demonstrate interaction treatment with main effect for treatment (F = 6.8; p = .02; η2 = .29)	• Marginally significant interaction effect for ADAS-Cog and MMSE • MMSE demonstrated main effect for treatment, possibly due to performance decline in controls • ↑ brain energy metabolism in the MCI intervention subgroup.	0.661
10.	Gagnon and Belleville (2012)	RCT, double-blind, active control	n = 24 (MCI-SD/MD) Tx n = 12 Cntl n = 12 Age: Fixed Priority $\overline{x}$ = 67.00 s = 7.80 Variable Priority $\overline{x}$ = 68.42 s = 6.04	• 6, 60 min sessions (3×/ week for 2 weeks) • Computer-based restorative training method to increase attentional control and meta-cognition	1° - (modified) Dual task visual detection/ classical digit span 2° - TEA, TMT-A/B, DAQ, WBS	• Significant: Fixed priority group vs. variable priority group (*p* < .05) improvement in cost scores for visual detection accuracy was 3.39 to 25.98 respectively. Alpha-arithmetic task, both accuracy and reaction time showed significant main effects of Attention, F (1, 22) = 14.89, (*p* = .001) and F (1, 22) respectively, and of Intervention, F (1, 22) = 72.80, p = .001, and F (1, 22) = 8.18, p = .01, respectively • Trails A: main effect of Intervention F (1, 22) = 15.91, p = .001, η2 = 0.42 (all participants = faster) • No significant impact on WBS	• Cog intervention may ↑ attentional control in pts. with MCI and an executive deficit.	0.661
11.	Giuli et al. (2016)	RCT, inactive control	*n* = 97 Tx *n* = 48 Cntl *n* = 49 Age: Tx: $\overline{x}$ = 76.0 s = 6.3 Cntl: $\overline{x}$= 76.5 s = 5.7	• 10, 45 min sessions (1×/ week) • Patient tailored, individual 1:1 sessions. Multi-component strategies in orientation, memory, categorization and clustering, psychological support, psychoeducation, as well as education regarding healthy lifestyles	MMSE, CSST, DS-FWD & DS-BWD, Prose memory, VPA, AMT, Semantic fluency, phonemic fluency, CDR, GDS-30, PSS, ALD, IADL, MAC-Q, Questionnaire of confidence	• Significant improvements in MCI group after training noted in MAC-Q (p < 0.001), Prose memory (*p* < 0.004), VPA (*p* < 0.018), CSST (*p* < 0.040), and AMT (*p* < 0.001) • No differences observed in DS-FWD, DS-BWD, semantic fluency, or phonemic fluency	• Training in cognitive strategies, psychological support and education regarding healthy lifestyle were associated with improvements in subjective memory complaints as well as an increase in prose recall, word-pairing recall, and sustained attention from baseline scores • Individuals receiving training exceeded performance on outcome measures of passive controls in DS-FWD, DS-BWD, GDS, MAC-Q, IADLs, Prose memory, word pairing, CSST, and AMT.	−0.713
12.	Greenaway et al. (2012)	RCT, inactive control	*n* = 40 (aMCI-SD) Tx n = 20 Cntl *n* = 20 Age: Tx: $\overline{x}$ = 72.7 s = 6.9 Cntl: $\overline{x}$= 72.3 s = 7.9	• 12, 60 min sessions (2×/ week for 6 weeks) • Dyad-based (participant and partner) receiving compensatory training memory strategies (Memory Support System)	DRS-II, MMSE, WMS-R/III, CERAD Word List, WMS-R/ III VR, ECog, QoL-AD, CBQ, Chronic Disease Self-Efficacy Scale, Adherence Assessment	• No significant effects of treatment in vs. control in DRS-II or MMSE • Treatment Ecog significant at 8wk [t (17) = 2.4, *p* < 0.05] but not at 6 months • MCI sense of memory self-efficacy at the end, t (15) = −3.1 (p < 0.01) vs. controls, t (33) = 2.4 (p = 0.02)	• ↑ Functional ability and sense of self-efficacy compared with controls out to 8-week follow-up • ↑ in ADLs and ↑ ECog scores	−1.401
13.	Hampstead et al. (2012a)	RCT, single-blind, inactive control	n = 49 Tx = 11 Cntl = 10 Age: aMCI mnemonic: $\overline{x}$= 73.5 s = 10.1 aMCI exposure: $\overline{x}$ = 70.5 s = 5.8	• 3, 60–90 min sessions, over 2 weeks • Individual-based, one-on-one compensatory training in object-location built on face-name association and mnemonic strategies	MMSE, RBANS, TMT, GDS, FAQ, ILV, F-N Accuracy, fMRI imaging	Correlation (Spearman’s Rho) results: • Significant RBANS DMI: aMCI mnemonic group = .67 (*p* = .009), healthy + MCI mnemonic group = .68 (*p* < .001), and healthy + MCI exposure = .68 (p < .001) •Significant Trails: aMCI mnemonic group = .57 (*p* = .03) •Significant ILV: healthy + MCI mnemonic group = −.75 (p = <.001) and aMCI mnemonic group = −.81 (p = .001) •Significant amygdala: healthy + MCI mnemonic group = .54 (p = .01)	• Mnemonic strategies ↑ memory for specific content for at least 1 month	−0.0.26
14.	Herrera et al. (2012)	RCT, single-blind, active control	n = 22 (aMCI-MD) Tx *n* = 11 Cntl n = 11 Age: Tx: $\overline{x}$= 75.09 s = 1.97 Cntl $\overline{x}$= 78.18 s = 1.44	• 24, 60 min sessions (2×/ week for 12 weeks) • Computer-based restorative training in memory & attention	DS-FWD, DS-BWD, DMS48, 12-word recall (BEM-144), 16-FR/CR test, MMSE, Doors/ People memory	Significant cognitive outcomes: • Trained group immediately at end of training - Doors A (9.64 ± 0.53), Doors B (6.36 ± 0.66), DMS48 (96.91 ± 0.58), forward digit span (4.91 ± 0.21), BEM-144 (7.28 ± 0.26), 16-FR/CR test (42.91 ± 0.76), and MMSE (2.09 ± 0.22) • Trained group 6 months after training - Doors A (8.55 ± 0.39), forward digit span (4.92 ± 0.23), BEM-144 (6.86 ± 0.52)	• Cog training associated with ↑ episodic recall and recognition post-training which was also sustained at 6 months post-training	−0.026
15.	Jean et al. (2010a)	RCT, single-blind, active controls	n = 22 (aMCI) Tx n = 11 Cntl n = 11 Age: Tx: $\overline{x}$ = 68.55 s = 9.16 Cntl: $\overline{x}$= 68.55 s = 5.91	• 6, 45 min sessions (2×/week for 3 weeks) • Individual-session restorative focused training in face-name associations using errorless learning and spaced retrieval	1° - Training measure (free recall and cued recall) 2° - CVLT-II, DRS-2, F-N Recall, MMSE, RBMT, MMQ, SES	• Total profile score RBMT improved significantly (*t* = 7.687, p < .001) while age, MMSE total score, DRS-2 total score, DRS-2 memory subscale score and CVLT-II delayed free recall did not significantly improve model despite the fact that they correlated significantly with the predicted variable	• ↑ explicit residual memory important factor leads to ↑ outcome when using errorless learning or errorful learning to learn face–name associations. • Structured cog training, focusing on memory issues, w/ pt. support, is effective in MCI-A, regardless of the techniques	−0.026
16.	Jeong et al. (2016)	RCT, double-blind, inactive control	*n* = 147 (aMCI) Tx *n* = 71 Cntl *n* = 76 Age: Tx: $\overline{x}$ = 70.8 s = 6.9 Cntl: $\overline{x}$= 71.6 s = 6.5	• 24, 90 min sessions (2×/week for 12 weeks) • Group-based, 5 per group • Multicomponent cognitive training (memory, attention, executive functions, language, reality orientation, visual-spatial functions), activities to improve ADLs, knowledge for health and daily life, reminiscence therapy and discussion	1° - ADAS-Cog 2° - MMSE, Digit Symbol Coding, Stroop, Animal fluency, COWAT, SRT, DS-FWD, DS-BWD, CDR, GDS-15, NPI, Bayer ADL, PRMQ, AD8, PMT, MMT-Strategy, Composite scores	• Improvements observed in ADAS-Cog (p = 0.03), PMT (*p* = 0.03), AD8, and NPI • No differences observed in composite scores for logical memory, working memory, executive functions, MMSE, or CDR	• Benefit of cognitive intervention displayed in ADAS-Cog, prospective memory, and informant rating of subject functioning • Comprehensive multi-modal with multiple approaches targeting multiple domains vs. one approach or single domains. • Benefits of training maintained at 6 months post-completion	2.035
17.	Lam et al. (2015)	RCT, double-blind, inactive control	*n* = 276 (MCI) Tx *n* = 145 Cntl *n* = 131 Age: Tx: $\overline{x}$ = 74.4 s = 6.4 Cntl: $\overline{x}$= 75.4 s = 6.1	• 48, 60 min sessions (3×/ week for 4 months [Time 1], 12 months total) • Group-based, 12–15 per group with homework assignments • Multicomponent or lifestyle activities categorized into cognitive, physical, social, and recreational (33 in total). Cognitive group attended cognitively demanding activities (i.e. reading, discussing newspapers etc.)	1° - CDR-SOB 2° - ADAS-Cog, CMMSE, List Learning (delayed recall), Digit Span, Visual Span, CVFT, C-TMT, MIC, CSDD, CDAD	• No group differences were observed post-training (12 month) • No differences observed in CDR-SOB, CDAD, or CMMSE scores	• No change in general cognition scores (CRD-SOB) over one year may suggest plateau of decline (stabilization), a possible benefit of structured lifestyle activities	2.035
18.	Mowszowski et al. (2014)	RCT, double-blind, inactive control	n = 40 (MCI) Tx *n* = 25 Cntl n = 15 Age: Tx: $\overline{x}$ = 74.4 s = 6.4 Cntl: $\overline{x}$= 75.4 s = 6.1	• 14, 120 min sessions (2×/ week, 7 weeks) • Group-based, 10 per group (60 min per group, 2× per week) followed by individually tailored computer-based training using NEAR model • Multicomponent, computer-based cognition training (not-specified) and psychoeducation	1° - EEG 2° - WTAR, MMSE, RAVLT, FAS, Semantic Fluency (Animals), WAIS-III-DS (total), TMT-B	• Cognitive trained group evidenced improvements in phonemic fluency (FAS), vs. controls who declined during the waitlist period • No differences observed in DS, RAVLT, semantic fluency, or TMT-B	• Data from EEG findings suggest enhanced response from frontal and central regions following cognitive training • Cognitive training associated with improvements in phonemic fluency • No increase observed in attention/ working memory, verbal learning & memory, semantic fluency or cognitive flexibility	−0.026
19.	Olchik et al. (2013)	RCT, single-blind, inactive control	*n* = 30 (MCI) Tx n = 16 Cntl n = 14 Age: Tx: $\overline{x}$ = 70.3 s = 4.3 Cntl: $\overline{x}$= 70.2 s = 5.7	• 8, 90 min sessions (2×/ week, 4 weeks) • Group-based, 10 per group, comprised of both MCI and normal controls • Group sessions multicomponent training focused on memory with each session beginning with explanatory/ education followed by training in a memorization target task/ strategy (active attention, categorization, association, or visual imagery), and exercises to practice the strategy.	MMSE, Lawton IADL, CRD, Semantic Fluency (Animals), COWAT (FAS), RAVLT, RBMT	• There were no statistically significant effects for memory training across groups post-training • Memory training was associated with greater improvement in FAS and RAVLT scores (compared to other groups examined) • MCI participants demonstrated more significant increase in scores than normal controls in RAVLT (immediate & delay) and RBMT (screening)	• Memory training resulted in higher change in scores from pre-training values, beyond education trained or inactive controls • MCI individuals appeared to benefit more from training than normal controls, supporting the compensation hypothesis • Memory training was not associated with improvements in cognitive outcomes of fluency (semantic or phonemic), verbal list learning & recall, or general memory performance	−1.401
20.	Polito et al. (2015)	RCT, single-blind, inactive control	*n* = 44 (MCI) Tx n = 22 Cntl *n* = 22 Age: Tx: $\overline{x}$ = 74.0 s = 1.4 Cntl: $\overline{x}$= 74.3 s = 1.7	• 10, 100 min sessions (2×/ week, 5 weeks) • Group-based, 7–8 per group • Group sessions with multicomponent training using body awakening, reality orientation, and multiple compensatory cognitive exercises. Exercises were designed to stimulate attention (auditory and visual), executive reasoning, language (fluency), semantic memory, visual perception, encoding, information storage, nonverbal learning and executive problem solving.	MMSE, MOCA, and CSST	• Participants receiving either cognitive training or sham treatment both demonstrated a significant improvement in MMSE and MOCA scores • Improvements from baseline scores were observed in the MCI trained group post-training, although this did not reach significance for any outcome measure (MMSE, MOCA, & CSST)	• Cognitive training was not associated with an increase in cognitive performance on outcome measures • An increase in performance observed in the sham training group (inactive controls) may be attributed to a placebo effect or, possibly, represent practice effects	0.661
21.	Rapp et al. (2002)	RCT, single-blind, inactive control	n = 19 (MCI) Tx n = 9 Cntl n = 10 Age: Tx: $\overline{x}$ = 73.33 s = 6.61 Cntl: $\overline{x}$= 75.10 s = 7.03	• 6, 120 min sessions (1× /week for 6 weeks) • Group-based, multicomponent training using education, relaxation training, memory skills training, and cognitive-restructuring	CERAD neuropsychological battery, MMSE, Face-Name, MFQ, Memory Controllability Inventory, POMS	• Mean values noted for word list delay of trained group. The data for pre-test, post-test, and follow-up was [3.56; SD = 2.92], [8.44; SD = 4.22], and [6.71; SD = 3.99]. Follow-up was significant (*p* < 0.07) • Pt’s rated their memory ability higher than controls (i.e. MCI-Present Ability scale, *p* = 0.008) • Training led to ↑ expectations for future improvement (i.e. MCI-Potential Improvement: *p* = 0.005) and ↓ expectations for cognitive decline MCI Inevitable decline: *p* = 0.06) • No change in objective laboratory memory tasks following training	• Cognitive/ behavioral group intervention targeting memory performance and memory appraisals can be effective at changing perceptions of memory ability in a high-risk population of older adults with MCI • Older adults with MCI may need more skills training to achieve and maintain performance improvements.	−0.713
22.	Rojas et al. (2013)	RCT, inactive control	n = 30 (MCI) Tx n = 15 Cntl *n* = 15 Age: Tx $\overline{x}$= 72.00 s = 14.29 Cntl: $\overline{x}$= 76.93 s = 7.05	• 52, 120 min sessions (2× /week for 6 months) • Group-based multicomponent intervention program including cognitive-stimulation, cognitive training, and education	1° - MMSE, CDR 2° - SMB, SF, BNT, PhF, Verbal Fluency, WASI, Similarities and Matrix reasoning, Block Design, TMT A/B, digit span forward/backward WAI-III, QoLQ, NPI, ADL Scale	• Trained group: significant mean of change for BNT [−2.84, *p* = .04] and SF [−3.03, *p* = .004] • Non-trained group: significant mean of change for MMSE [1.77, *p* = .002], Mem-REC [1.00, *p* = 0.036], SF [2.40, *p* = .007], CDR [−.01, *p* = .02] • No significant differences on secondary outcome measures	• Training group improved on BNT and semantic fluency	−1.401
23.	Schmitter-Edgecombe and Dyck (2014)	RCT, single-blind, inactive control	n = 46 (MCI) Tx *n* = 23 Cntl n = 23 Age: Tx: $\overline{x}$= 72.96 s = 7.05 Control $\overline{x}$= 73.35 s = 7.89	• 20, 120 min sessions, (2×/ week for 10 weeks) • Post-Booster: 120 min session (1× /month for 9 months) • Group-based, care-dyad, multicomponent training including workbook lessons, and education workshop	WTAR, TICS, MMAA, EFPT, ADL-PI, RBANS, QOL-AD, CSE, GDS, RBMT-II	• Treatment group performed better than controls on RBMT-II F (1, 43) = 4.20, *p* < .05, n^2^_p_ = .09/ F (1, 22) = 6.84, *p* = .01, n^2^_p_ = .24; RBANS Memory Index Imm F (1, 43) = 4.64, p < .05, n^2^_p_ = .10; RBANS Memory Index Delay = ns • MCI pre vs. post show improvements in RBANS Memory Index Imm F (1, 22) = 14.41, *p* < .001, n^2^_p_ = .40; and Delayed F (1, 22) = 9.79, *p* < .005, n^2^_p_ = .31 • Better post-test performance on MMAA and EFPT bill paying subtest. No significant post-test differences in MCI for coping strategies, quality of life, or depression	• Training group demonstrated improvements in everyday memory and immediate memory index as compared to controls • Training group demonstrated gains in memory performance comparing pre- to post- training	0.661
24.	Tsolaki et al. (2011)	RCT, inactive control	*n* = 201 (MCI) Tx *n* = 122 Cntl n = 76 Age: Tx: $\overline{x}$= 68.45 s = 6.99 Cntl: $\overline{x}$= 66.86 s = 8.79	• 60, 90 min sessions, (3×/ week for 5 months) • Booster session to subset 11 months after training • Group-based, multicomponent training in cognitive strategies, cognitive stimulation, and psycho-therapeutic techniques	HVLT, RAVLT, RBMT, Digit Symbol, FUCAS, BNT, FRSSD, MMSE	• Significant values of experimental group: ↑ general cognitive performance (*p* = 0.000), abilities of attention (*p* < 0.001), language (*p* = 0.006), verbal memory (p = 0.000), executive function (p = 0.000), visual perception (p = 0.000) and activities of daily living (ADL) (*p* = 0.013) • Ctrl group: ↓ in observed ADLs (*p* = 0.004)	• ↑ cog performance and generalized benefit. • Trained group demonstrate improvement in verbal memory, visual-constructive abilities, and executive functions • MCI group show improvement in general cognitive performance, attention, language, verbal memory, executive functions, visual perception, and activities of daily living.	−0.026
25.	Valdes et al. (2012)	RCT, single-blind, inactive control	*n* = 195 (MCI mixed) Tx *n* = 85 Cntl *n* = 110 Age: Tx: $\overline{x}$= 76.95 s = 6.53 Control $\overline{x}$= 78.34 s = 6.3	• 10 sessions, 60 min/ group sessions (5-week duration) • Group-based, computer administered restorative training using a standardized set of visual attention tasks designed to improve speed of information processing (SOPT)	RBMT, RAVLT, RBMT, LS, WS, Computerized UFOV	• SOPT improved UFOV in MCI relative to controls (F1,185) = 81.83, p < 0.001 • All subtypes of MCI appear to benefit from SOPT relative to controls • aMCI subtype appeared to experience greatest benefit from SOPT • MCI who received SOPT show greater rate of improvement over 5 years relative to controls • There was no difference in the slope of change between MCI subtypes across the 5-year period and gains of SOPT were maintained across 5 years	• Individuals with MCI benefit from SOPT • All MCI subtypes appear to benefit from SOPT relative to controls • Individuals with single non-amnestic and multi-domain subtypes demonstrate the greatest immediate improvement • aMCI individuals show the least UFOC improvement from pre- to immediate post-training • Benefits of SOPT training in MCI remain relatively stable across 5-years	−0.026
26.	Vidovich et al. (2015)	RCT, single-blind, active control	*n* = 160 (MCI) Tx n = 80 Cntl *n* = 80 Age: Tx: $\overline{x}$= 75.1 s = 6.1 Cntl: $\overline{x}$= 74.9 s = 5.5	• 10, 90 min sessions (2×/ week for 5 weeks) • Group-based, multicomponent training in cognition, activities related to enhance attention, memory, and executive functions as well as methods to adapt these to everyday life	CAMCOG-R, CVLT-II, WAIS-III DS, WAIS-III SS, TMT-A/B, COWAT	• Training did not affect CAMCOG-R scores over time, relative to controls • Training demonstrated marginally better scores in DS-FWD • Training had no effect on any other outcome measure related to cognition	• With the exception of attention (DS), training showed no effect on cognition either immediately, one-year or two-years post training. • Illness progression and changes in cognitive status over time may limit findings	−0.026

Please see Supplemental Table S4 for a list of abbreviations

In general terms, the types of interventions employed were multidomain including lifestyle elements such as exercise and social support (7/26; 26.92%), general cognitive interventions (7/26; 26.92%), specific mnemonic memory techniques (3/26; 11.54%), computer-based interventions (7/26; 26.92%), and highly developed specialized tasks (2/26; 7.69%). Categorizing interventions with more structured definitions found studies employed cognitive stimulation = 0/26 (0%), restorative training = 8/26 (30.77%), compensatory training = 3/26 (11.54%), and multicomponent approaches = 15/26 (57.69%). The primary content of interventions or domains targeted by treatment generally focused on working memory (7.69%), speed of information processing (7.69%), memory (34.62%), and multidomain training (including components associated with lifestyle and socialization, 50.00%), although there was some overlap in intervention content, strategies applied, and targeted domains (i.e. Jeong et al. 2016). The modes of training conducted were in group format (46.15%), individual plus dyad training (15.38%), and computer based programs (38.46%). The majority of studies completed post-training evaluations within two weeks or less (80.77%; 21/26). Five studies performed follow-up assessments ranging from four to twenty-six weeks (Jean et al. 2010a; Hampstead et al. 2012a; Valdes et al. 2012; Rojas et al. 2013, and Tsolaki et al. 2011, respectively). Treatment duration was roughly divided evenly at either short (8 weeks or less, 46.15%) or long duration (greater than 8 weeks, 53.85%). The majority of control groups were passive (received no training), waitlisted, or provided standard of care (57.69%) while the remaining engaged in an active, non-trained program (42.31%). Thirty-one percent (30.77%; 8/26) attempted to control for potential confound of practice effects through the use alternate or parallel versions of test instruments. The remaining studies (69.23%; 18/26) did not appear to account for practice effects or did not report this in their study. Other variables such as IQ, time since diagnoses, date of onset, etc. could not be considered for extraction due to lack of data across studies.

### Risk of Bias – Individual Studies

While point estimate effect sizes were ultimately used to determine training effect, an NIH derived total score and standard score (*z*) were calculated for each study. This is presented in Table 1.

### Synthesis of Results

A series of meta-analyses were conducted to investigate the effects of training overall (all measures combined) as well as by cognitive domain, training type, and domain targeted (intervention content). Please see Table 2 for an overview of point estimates and summary statistics for each element of interventions examined. Meta-regression and post hoc analyses were also performed to explore possible influence of moderator variables.

**Table 2:** Summary of meta-analyses: By domain, type of training and intervention content

			*k*	Hedges’ g		HKSJ	*p*	*Q*	*df*	*p*	*I* ^2^	*τ* ^*2*^	T&F	Egger’s	*p*
				*g*	*p*	(SMD)							(Trimmed)	Intercept	
Overall – All Interventions & Outcomes			26	0.454	0.003	–	–	205.409	25	0.000	87.829	0.484	0	3.036	0.016
Cognitive Domain															
	Mental Status & General Cognition		16	0.216	0.003	0.218	0.007	19.462	15	0.194	22.938	0.017	7	1.265	0.057
	Working Memory		12	0.614	0.000	0.627	0.011	43.068	11	0.000	74.459	0.227	0	3.113	0.030
	Speed of Information Processing		6	−0.434	0.235	−0.441	0.139	58.656	5	0.000	91.476	0.701	2	1.861	0.656
	Language		7	0.511	0.000	0.519	0.010	13.457	6	0.000	55.141	0.072	3	2.362	0.083
	Visual-Spatial Ability		2	–	–	–	–	–	–	–	–	–	–	–	–
	Memory														
		Verbal + Non-Verbal Combined	20	0.659	0.000	0.675	0.001	90.898	19	0.000	79.098	0.277	0	2.565	0.015
		Verbal	15	0.758	0.000	0.775	0.013	95.811	14	0.000	85.388	0.421	0	2.894	0.042
		Non-verbal	5	0.570	0.006	0.593	0.054	5.082	4	0.279	21.292	0.047	2	3.272	0.187
	Executive Functions		13	0.575	0.019	0.585	0.158	126.404	12	0.000	90.507	0.669	0	2.718	0.240
Intervention Type															
	Cognitive Stimulation		0	–	–	–	–	–	–	–	–	–	–	–	–
	Restorative		8	0.541	0.288	0.568	0.271	111.092	7	0.000	93.699	1.886	0	8.356	0.000
	Compensatory		2	–	–	–	–	–	–	–	–	–	–	–	–
	Multicomponent		16	0.398	0.001	0.404	0.013	55.511	15	0.000	72.978	0.146	0	1.819	0.128
Intervention Content – Cognitive Domain Targeted															
	Working Memory		2	–	–	–	–	–	–	–	–	–	–	–	–
	Speed of Information Processing		2	–	–	–	–	–	–	–	–	–	–	–	–
	Language		0	–	–	–	–	–	–	–	–	–	–	–	–
	Visual-Spatial Ability		0	–	–	–	–	–	–	–	–	–	–	–	–
	Memory		7	1.219	0.007	1.099	0.049	51.777	6	0.000	88.412	1.219	0	3.397	0.510
	Executive Functions		0	–	–	–	–	–	–	–	–	–	–	–	–
	Multidomain		13	0.230	0.000	0.232	0.003	12.713	12	0.390	5.612	0.003	2	1.144	0.100

1. **Intervention Effects – Overall**: The summary effect of interventions overall demonstrated a significant, moderate effect on cognition (Hedges’ *g* observed = 0.454; 95% CI [0.156, 0.753]; *Z* = 2.983; *p* = 0.003). The HKSJ calculation to adjust for a small number of studies was not conducted as the number of studies exceeded the upper limit of twenty (*k* = 26). Heterogeneity was significant and large; an anticipated finding in keeping with the diverse range of training employed and number of outcomes administered (*Q* = 205.409; *df* = 25; *p* = 0.000; *I* ^2^ = 87.829%; τ^2^ = 0.484). Visual inspection of the funnel plot was somewhat asymmetrical with three outliers observed. There were no adjustments for possible publication bias after calculation of Duval and Tweedie’s trim-and-fill method. Egger’s regression intercept was suggestive of small-study effects (Intercept = 3.036; *t* = 2.60; two-tailed *p* = 0.016). Please see Fig. 2 for a forest plot of the effects and overall summary effect and Fig. S2 for a funnel plot of the standard error (SE) by Hedges’ *g*. Please see Table S7 in the supplemental materials for a list of effect sizes, confidence intervals, and *p*-values for outcome measures used in each study (Note: The effect sizes reported in this table assume independence and are for general information only). Sensitivity analysis examining effect sizes and measures of dispersion at various levels of correlation between test instruments demonstrated adequate mean effects and weights consistent with the values used a conservative estimate of combined outcome (please see Table S8).

**Fig. 2:**
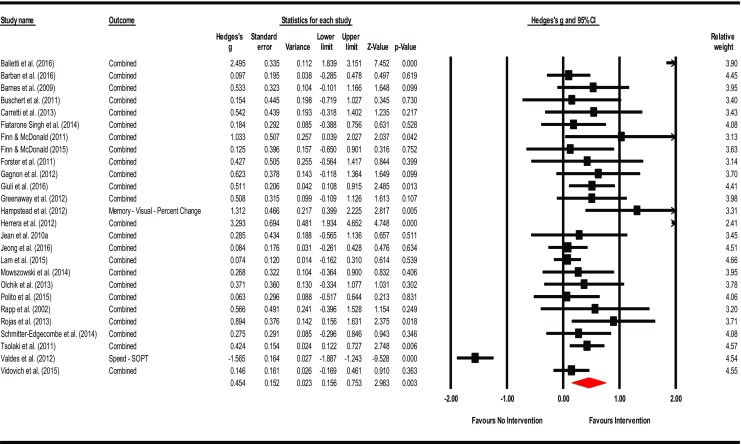
Effects of cognitive interventions on all outcome measures*.* Test for heterogeneity *Q* = 205.409, *df* = 25; *p* = 0.000; *I* ^*2*^ = 87.829; *τ* ^*2*^ = 0.484
2. **Intervention Effects – By Cognitive Domain**: Please see Table 3 for a breakdown by domain including the study, outcome measures, effect sizes, indices of heterogeneity, and prediction intervals.

**Table 3:** Effect sizes by cognitive domain: Effects of outcome measures, confidence intervals and prediction intervals

Cognitive domain	Study	Measure	n	g	Actual/ observed intervals		p	*Q*	*df* (*Q*)	p	*I* ^2^	τ^2^	τ	Prediction intervals	
					Lower (95%)	Upper (95%)								Lower (95%)	Upper (95%)
Mental status/ general cognitive															
	Barban et al. (2016)	MMSE	106	0.111	−0.271	0.493	0.569								
	Barnes et al. (2009)	RBANS Total	36	0.374	−0.272	1.020	0.256								
	Buschert et al. (2011)	Combined (MMSE + ADAS-Cog)	22	0.387	−0.514	1.288	0.400								
	Carretti et al. (2013)	Cattell – CFT	20	0.818	−0.059	1.695	0.068								
	Fiatarone Singh et al. (2014)	ADAS-Cog	22	−0.057	−0.626	0.512	0.844								
	Forster et al. (2011)	Combined (MMSE + ADAS-Cog)	18	0.427	−0.564	1.417	0.399								
	Greenaway et al. (2012)	Combined (MMSE + DRS-2)	40	0.508	−0.109	1.126	0.107								
	Jean et al. (2010a)	Combined (MMSE + RBMT + DRS-2)	20	0.305	−0.545	1.156	0.482								
	Jeong et al. (2016)	Combined (MMSE + ADAS-Cog)	147	−0.086	−0.430	0.258	0.623								
	Lam et al. (2015)	Combined (CMMSE + ADAS-Cog)	276	−0.059	−0.294	0.177	0.626								
	Olchik et al. (2013)	RBMT – Screening Score	30	0.132	−0.567	0.830	0.712								
	Polito et al. (2015)	Combined (MMSE + MOCA)	44	0.079	−0.501	0.660	0.788								
	Rojas et al. (2013)	MMSE	30	0.926	0.191	1.661	0.014								
	Schmitter-Edgecombe et al. (2014)	RBMT-II	46	0.336	−0.236	0.908	0.250								
	Tsolaki et al. (2011)	Combined (MMSE + MOCA)	176	0.486	0.182	0.790	0.002								
	Vidovich et al. (2015)	CAMCOG-R	155	0.247	−0.066	0.561	0.122								
		Random - Observed (k = 16)		0.216	0.076	0.356	0.003	19.462	15	0.194	22.928	0.017	0.131	−0.103	0.5349
		HKSJ Point Estimate Adjustment (SMD)		0.218	0.070	0.366	0.007								
Working memory/attention															
	Balietti et al. (2016)	Combined (DS-FWD + CSST + Matrices)	70	1.469	0.939	2.000	0.000								
	Barnes et al. (2009)	WMS-III Spatial Span	36	0.663	0.006	1.321	0.048								
	Carretti et al. (2013)	Combined (DS-FWD, BWD, CWMS, VS)	20	0.474	−0.383	1.331	0.279								
	Finn and McDonald (2011)	Visual Sustained (RVP)	16	1.056	0.060	2.052	0.038								
	Finn and McDonald (2015)	WMS-IV Spatial Span	24	0.404	−0.377	1.185	0.310								
	Gagnon and Belleville (2012)	Combined (TEA + Visual + DS + DA)	24	0.893	0.173	1.613	0.015								
	Giuli et al. (2016)	Combined (DS + CSST + Matrices)	97	0.414	0.014	0.815	0.043								
	Herrera et al. (2012)	Combined (DS-FWD + DS-BWD)	22	2.941	1.708	4.174	0.000								
	Jeong et al. (2016)	Composite Score (DS-FWD + DS-BWD)	147	0.106	−0.238	0.449	0.547								
	Mowszowski et al. (2014)	WAIS-III DS Total	40	0.254	−0.376	0.884	0.429								
	Polito et al. (2015)	CSST	44	0.031	−0.550	0.611	0.918								
	Vidovich (2015)	Combined (DS-FWD + DS-BWD)	155	0.142	−0.172	0.456	0.375								
		Random - Observed (k = 12)		0.614	0.285	0.943	0.000	43.068	11	0.000	74.459	0.227	0.476	−0.512	1.7395
		HKSJ Point Estimate Adjustment (SMD)		0.627	0.176	1.078	0.011								
Speed of information processing															
	Buschert et al. (2011)	TMT-A	22	−0.327	−1.140	0.486	0.431								
	Fiatarone Singh et al. (2014)	SDMT	22	−0.272	−0.844	0.299	0.350								
	Finn and McDonald (2015)	D-KEFS Number Seq.	24	−0.210	−0.985	0.565	0.596								
	Gagnon et al. (2012)	TMT-A	24	−0.280	−1.057	0.497	0.480								
	Valdes et al. (2012)	SOPT	195	−1.565	−1.887	−1.243	0.000								
	Vidovich et al. (2015)	Combined (TMT-A + Symbol Search)	155	0.137	−0.179	0.454	0.394								
		Random - Observed (k = 6)		−0.434	−1.150	0.282	0.235	58.656	5	0.000	91.476	0.701	0.837	−2.9702	2.1022
		HKSJ Point Estimate Adjustment (SMD)		−0.441	−1.085	0.203	0.139								
Language															
	Balietti et al. (2016)	Semantic Fluency	70	1.031	0.536	1.525	0.000								
	Fiatarone Singh et al. (2014)	Semantic Fluency	22	0.609	0.027	1.191	0.040								
	Giuli et al. (2016)	Semantic Fluency	97	0.134	−0.261	0.530	0.505								
	Lam et al. (2015)	Semantic Fluency	276	0.223	−0.014	0.459	0.065								
	Mowszowski et al. (2014)	Semantic Fluency	40	0.380	−0.252	1.013	0.239								
	Olchik et al. (2013)	Semantic Fluency	30	0.688	−0.031	1.407	0.061								
	Rojas (2013)	Combined (BNT + Semantic Fluency)	30	0.967	0.217	1.717	0.011								
		Random - Observed (k = 7)		0.511	0.231	0.790	0.000	13.457	6	0.000	55.414	0.072	0.269	−0.2698	1.2918
		HKSJ Point Estimate Adjustment (SMD)		0.519	0.179	0.859	0.010								
Visual-spatial															
	Barnes et al. (2009)	RBANS – Figure Copy	36	0.483	−0.089	1.054	0.098								
	Tsolaki et al. (2011)	Combined (RCFT–Copy + Clock Drawing)	176	0.374	0.072	0.676	0.015								
		Random - Observed (k = 2)		0.398	0.131	0.665	0.003	–	–	–	–	–	–	–	–
		HKSJ Point Estimate Adjustment (SMD)		–	–	–	–								
Memory – all (verbal and non-verbal – immediate and delay)															
	Balietti et al. (2016)	Combined (Story + Word Pairs)	70	2.884	2.218	3.550	0.000								
	Barban et al. (2016)	Memory – List Delay (RAVLT)	106	0.083	−0.299	0.464	0.671								
	Barnes et al. (2009)	Memory – Delay (RBANS V + NV)	36	0.611	−0.044	1.266	0.068								
	Buschert et al. (2011)	Memory – Story Delay (RBANS)	22	1.276	0.385	2.167	0.005								
	Carretti et al. (2013)	Memory – List Delay	20	0.538	−0.318	1.394	0.218								
	Fiatarone Singh et al. (2014)	Combined (List, Story, BVMT-R)	22	0.216	−0.355	0.786	0.459								
	Finn and McDonald (2011)	Memory – Delay (PAL + PRM)	16	0.928	−0.054	1.910	0.064								
	Finn and McDonald (2015)	Combined (VPA I + VPA II)	24	0.166	−0.608	0.940	0.673								
	Giuli et al. (2016)	Combined (Stories Imm + Word Pairs)	97	0.678	0.271	1.084	0.001								
	Hampstead et al. (2012a)	Memory – Visual (Percent Change)	21	1.312	0.399	2.225	0.005								
	Herrera et al. (2012)	Combined (List Delay(s) + Figure Delay)	22	3.158	1.778	4.537	0.000								
	Jean et al. (2010a)	Combined (CVLT-II + Face-Name + RBMT)	20	0.093	−0.752	0.937	0.830								
	Jeong et al. (2016)	Memory – Composite (Verbal)	147	0.161	−0.183	0.505	0.360								
	Lam et al. (2015)	Memory – List Delay	276	0.190	−0.046	0.426	0.115								
	Mowszowski et al. (2014)	Memory – List Delay (RAVLT)	40	0.356	−0.276	0.989	0.269								
	Olchik et al. (2013)	Combined (RAVLT + RBMT Story)	30	0.468	−0.240	1.176	0.195								
	Rapp et al. (2002)	Combined (Word List, Story, & F-N Visual)	16	0.566	−0.396	1.528	0.249								
	Rojas et al. (2013)	Memory – List Delay	30	0.944	0.208	1.680	0.012								
	Schmitter-Edgecombe et al. (2014)	Combined (RBANS V + NV, RBMT-II)	46	0.244	−0.327	0.814	0.402								
	Vidovich et al. (2015)	Combined (CVLT-II)	176	0.325	0.009	0.640	0.044								
		Random - Observed (k = 20)	155	0.659	0.383	0.936	0.000	90.898	19	0.000	79.098	0.277	0.526	−0.4859	1.8039
		HKSJ Point Estimate Adjustment (SMD)		0.675	0.305	1.045	0.001								
Memory - verbal															
	Combined (List Delay + Story Recall)														
	Balietti et al. (2016)	Combined (Story Delay + Word Pairs)	70	2.824	2.165	3.483	0.000								
	Barban et al. (2016)	RAVLT (Delay)	106	0.083	−0.299	0.464	0.671								
	Buschert et al. (2011)	Story Delay (RBANS)	22	1.276	0.385	2.167	0.005								
	Carretti et al. (2013)	List Delay	20	0.538	−0.318	1.394	0.218								
	Fiatarone Singh et al. (2014)	Story Delay (WMS-III LM II)	22	0.296	−0.276	0.868	0.310								
	Finn and McDonald (2015)	VPA-II	24	0.182	−0.592	0.956	0.645								
	Giuli et al. (2016)	Word Pairs	97	0.812	0.401	1.223	0.000								
	Herrera et al. (2012)	Combined (List Delay)	22	4.644	3.053	6.234	0.000								
	Jean et al. (2010a)	CVLT-II List Long Delay	20	0.005	−0.839	0.849	0.991								
	Lam et al. (2015)	List Delay	276	0.190	−0.046	0.426	0.115								
	Mowszowski et al. (2014)	RAVLT Delay	40	0.356	−0.276	0.989	0.269								
	Olchik et al. (2013)	RAVLT Delay	30	0.487	−0.221	1.196	0.178								
	Rapp et al. (2002)	Combined (Shopping, Word list + Story)	16	0.654	−0.309	1.617	0.183								
	Rojas et al. (2013)	List Delay	12	0.944	0.208	1.680	0.012								
	Vidovich et al. (2015)	CVLT-II List Long Delay	155	0.291	−0.024	0.606	0.070								
		Random - Observed (k = 15)		0.758	0.382	1.133	0.000	95.811	14	0.000	85.388	0.421	0.649	−0.7034	2.2194
		HKSJ Point Estimate Adjustment (SMD)		0.775	0.188	1.362	0.013								
Memory – non-verbal															
	Fiatarone Singh et al. (2014)	Form Recall – (BVMT-R)	22	0.254	−0.317	0.825	0.383								
	Finn and McDonald (2011)	Combined (PAL + PRM)	16	0.928	−0.054	1.910	0.064								
	Hampstead et al. (2012a)	Visual – Percent Change	21	1.312	0.399	2.225	0.005								
	Herrera et al. (2012)	Figure Delay (Rey-O)	22	0.185	−0.620	0.991	0.652								
	Rapp et al. (2002)	Non-Verbal - Delay (Face – Name)	16	0.619	−0.339	1.577	0.205								
		Random - Observed (k = 5)		0.570	0.160	0.980	0.006	5.082	4	0.279	21.292	0.047	0.217	−0.3887	1.5287
		HKSJ Point Estimate Adjustment (SMD)		0.593	−0.016	1.202	0.054								
Executive functions															
	Balietti et al. (2016)	Phonemic Fluency	70	5.266	4.278	6.254	0.000								
	Buschert et al. (2011)	TMT-B	22	−0.954	−1.809	−0.099	0.029								
	Fiatarone Singh et al. (2014)	Combined (FAS, Similarities, Matrices)	22	0.248	−0.324	0.821	0.395								
	Finn and McDonald (2011)	Combined (SWM + CANTAB-IED)	16	1.096	0.094	2.097	0.032								
	Finn and McDonald (2015)	D-KEFS N-L Switching	24	0.099	−0.675	0.872	0.803								
	Gagnon and Belleville (2012)	Combined (TEAVis Elev + TMT-B)	24	0.129	−0.663	0.921	0.749								
	Giuli et al. (2016)	Phonemic Fluency	97	0.943	0.527	1.360	0.000								
	Jeong et al. (2016)	Composite Score	147	0.065	−0.278	0.408	0.711								
	Mowszowski et al. (2014)	Combined (FAS + TMT-B)	40	0.170	−0.462	0.802	0.598								
	Olchik et al. (2013)	FAS	30	0.270	−0.431	0.971	0.450								
	Rojas et al. (2013)	FAS	30	0.663	−0.053	1.380	0.069								
	Tsolaki et al. (2011)	FUCAS Planning	176	0.343	0.042	0.645	0.026								
	Vidovich et al. (2015)	Combined (FAS + TMT-B)	155	−0.103	−0.418	0.212	0.522								
		Random - Observed (k = 13)		0.575	0.093	1.056	0.019	126.40	12	0.000	90.507	0.669	0.818	−1.3045	2.4545
		HKSJ Point Estimate Adjustment (SMD)		0.585	−0.262	1.432	0.158

  - **Mental Status & General Cognition**: A total of sixteen studies reported scores for mental status and general cognition. The effect of training on MCI compared to controls was small and significant (Hedges’ *g* observed = 0.216; 95% CI [0.076, 0.356]; *Z* = 3.015; *p* = 0.003). The HKSJ calculation was also significant (HKSJ point estimate adjustment SMD = 0.218; 95% CI [0.070, 0.366]; *t* = 3.146; *df* = 15; *p* = 0.007). Test for heterogeneity was not significant (*Q* = 19.462; *df* = 15; *p* = 0.194; *I* ^2^ = 22.928%; τ^2^ = 0.017). Visual inspection of the funnel plot was asymmetrical and Duval and Tweedie’s trim-and-fill method (to the left) trimmed seven studies generating a very small adjusted point estimate reflective of probable publication bias (Adjusted point estimate = 0.073; 95% CI [−0.087, 0.233]; *Q* = 44.231). Egger’s regression intercept was not significant for small-study effects (Intercept = 1.265; *t* = 2.073; *p* = 0.057; two-tailed). The summary effects and forest plot for mental status and general cognition is presented in Fig. 3a and funnel plot is provided in the supplemental materials as Fig. S3a.

**Fig. 3:**
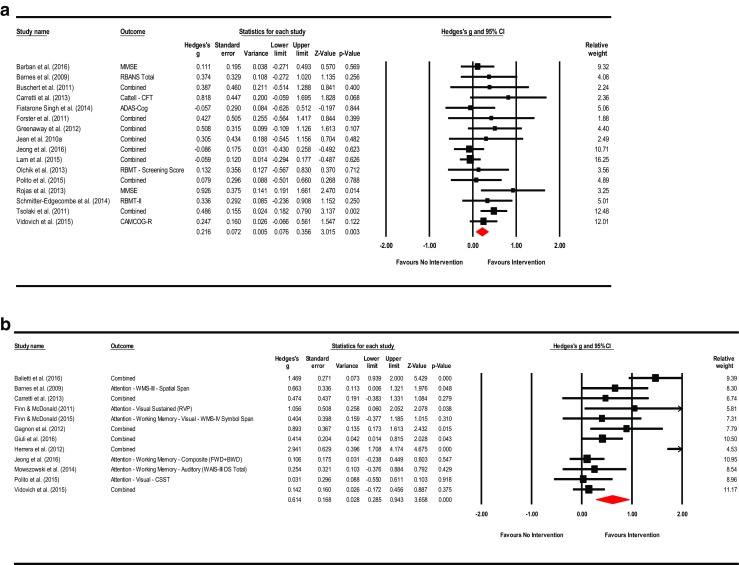

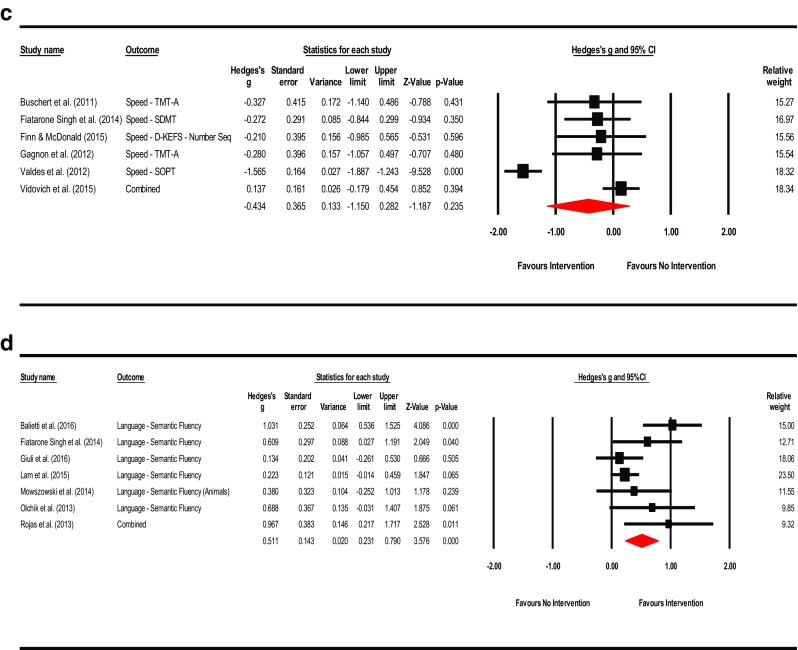

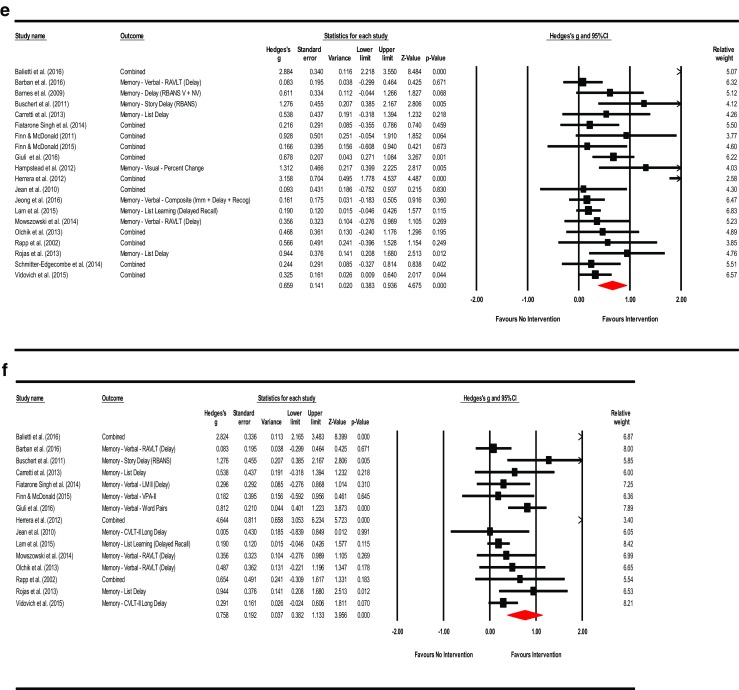

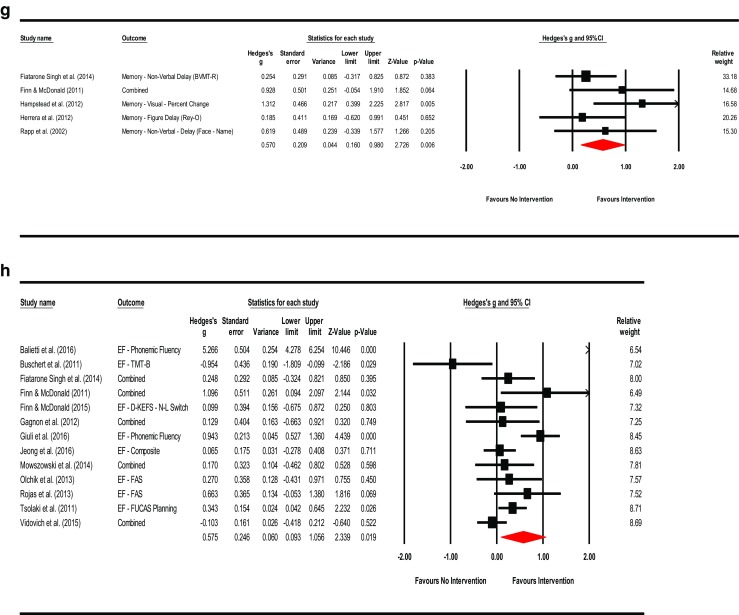
**a** Meta-analysis of interventions on mental status and general cognition*.* HKSJ point estimate adjustment SMD = 0.218; *t* = 3.146; *p* = 0.007. Test for heterogeneity *Q* = 19.462; *df* = 15; *p* = 0.194; *I* ^*2*^ = 22.928; *τ* ^*2*^ = 0.017. **b** Meta-analysis of interventions on working memory/attention. HKSJ point estimate adjustment SMD = 0.627; *t* = 3.062; *p* = 0.011. Test for heterogeneity *Q* = 43.068; *df* = 11; *p <*  0.001; *I* ^*2*^ = 74.459; *τ* ^*2*^ = 0.227. **c** Meta-analysis of interventions on speed of information processing. HKSJ point estimate adjustment SMD = −0.441; *t* = −1.759; *p* = 0.139. Test for heterogeneity *Q* = 58.656; df = 5; *p* = 0.000; *I* ^*2*^ = 91.476; *τ* ^*2*^ = 0.701. **d** Meta-analysis of interventions on language. HKSJ point estimate adjustment SMD = 0.519; *t* = 3.730; *p* = 0.010. Test for heterogeneity *Q* = 13.457; *df* = 6; *p* <  0.001; *I* ^*2*^ = 55.141; *τ* ^*2*^ = 0.072. **e** Meta-analysis of interventions on memory: verbal + non-verbal combined. HKSJ point estimate adjustment SMD = 0.675; *t* = 3.823; *p* = 0.001. Test for heterogeneity *Q* = 90.898; *df* = 19; *p <*  0.001; *I* ^*2*^ = 79.098; *τ* ^*2*^ = 0.277. **f** Meta-analysis of interventions on memory: verbal. HKSJ point estimate adjustment SMD = 0.775; *t* = 2.833; *p* = 0.013. Test for heterogeneity *Q* = 95.811; *df* = 14; *p <*  0.001; *I* ^*2*^ = 85.388; *τ* ^*2*^ = 0.421. **g** Meta-analysis of interventions on memory: non-verbal. HKSJ point estimate adjustment SMD = 0.593; *t* = 2.705; *p* = 0.054. Test for heterogeneity *Q* = 5.082; *df* = 4; *p* = 0.279; *I* ^*2*^ = 21.292; *τ* ^*2*^ = 0.047. **h** Meta-analysis of interventions on executive functions. HKSJ point estimate adjustment SMD = 0.585; *t* = 1.505; *p* = 0.158. Test for heterogeneity *Q* = 126.404; *df* = 12; *p* < 0.001; *I* ^*2*^ = 90.507; *τ* ^*2*^ = 0.669
  - **Working Memory**: A total of twelve studies reported outcome measures associated with working memory and attention. The summary effect was moderate and significant (Hedges’ *g* observed = 0.614; 95% CI [0.285, 0.943]; *Z* = 3.658; *p* = 0.000). Examination of the HKSJ result adjusting for the small number of studies was also significant (HKSJ point estimate adjustment SMD = 0.627; 95% CI [0.176, 1.078]; *t* = 3.062; *df* = 11; *p* = 0.011). Heterogeneity across studies was significant (*Q* = 43.068; *df* = 11; *p* <  0.001; *I* ^2^ = 74.459%; τ^2^ = 0.227). Visual inspection of the funnel plot was somewhat asymmetrical, with two outliers (Balietti et al. 2016 & Herrera et al. 2012) There were no adjustments after calculation of Duval and Tweedie’s trim-and-fill method. Egger’s regression intercept was significant suggestive of small study effects (Intercept = 3.113; *t* = 2.533; *p* = 0.030; two-tailed). A figure of effect sizes and forest plot is presented in Fig. 3b and a funnel plot is provided in the supplemental materials as Fig. S3b.
  - **Speed of Information Processing**: Six studies reported scores associated with speed of information processing. The summary effect size was moderate and non-significant (Hedges’ *g* observed = −0.434; 95% CI [−1.150, − 0.282]; *Z* = −1.187; *p* = 0.235). Calculation of the HKSJ adjustment was also not significant (HKSJ point estimate adjustment SMD = −0.441; 95% CI [−1.085, 0.203]; *t* = −1.759; *df* = 5; *p* = 0.139). Heterogeneity values were significant (*Q* = 58.656; *df* = 5; *p* = 0.000; *I* ^2^ = 91.476%; *τ* ^*2*^ = 0.701). Visual inspection of the funnel plot was asymmetrical and Duval and Tweedie’s trim-and-fill method trimmed two studies generating a moderate adjusted point estimate (Adjusted point estimate = −0.650; 95% CI [−1.232, −0.068]; *Q* = 80.117). Egger’s regression intercept was not significant (Intercept = 1.861; *t* = 0.480; *p* = 0.656; two-tailed). The mean of effect sizes and forest plot is presented in Fig. 3c and a funnel plot is provided in the supplemental materials as Table S3c.
  - **Language**: There were seven studies which reported outcome data related to language functioning. The summary effect size was moderate and significant (Hedges’ *g* observed = 0.511; 95% CI [0.231, 0.790]; *Z* = 3.576; *p* <  0.001). Calculation of the HKSJ adjustment was also significant (HKSJ point estimate adjustment SMD = 0.519; 95% CI [0.179, 0.859]; *t* = 3.730; *df* = 6; *p* = 0.010). Heterogeneity indicators were significant (*Q* = 13.457; *df* = 6; *p* = 0.000; *I* ^2^ = 55.141%; τ^2^ = 0.072). Visual inspection of the funnel plot was asymmetrical with one outlier (Balietti et al. 2016). Duval and Tweedie’s trim-and-fill method trimmed three studies generating a medium adjusted point estimate (Adjusted point estimate = 0.282; 95% CI [−0.022, 0.587]; *Q* = 30.609). Egger’s regression intercept was not significant (Intercept = 2.362; *t* = 2.158; *p* = 0.083; two-tailed). The mean of effect sizes and forest plot is presented in Fig. 3d and a funnel plot is provided in the supplemental materials as Table S3d.
  - **Visual-Spatial**: Two studies reported outcomes regarding visual-spatial ability. Given the number of studies fell below minimal cutoff of five, an analysis was not conducted for this domain.
  - **Memory**: A total of twenty studies reported outcome scores for memory; either verbal memory, non-verbal memory, or both combined. The overall effect was moderate and significant (Hedges’ *g* observed = 0.659; 95% CI [0.383, 0.936]; *Z* = 4.6675; *p* = 0.000). HKSJ adjustment was also significant (HKSJ point estimate adjustment SMD = 0.675; 95% CI [0.305, 1.045]; *t* = 3.823; *df* = 19; *p* = 0.001), however, heterogeneity values were significant (*Q* = 90.898; *df* = 19; *p* < 0.001; *I* ^2^ = 79.098%; τ^2^ = 0.277). Visual inspection of the funnel plot was largely asymmetrical with two outliers (Balietti et al. 2016 *g* = 2.884; Herrera et al., 2011 *g* = 3.158). There were no adjustments after calculation of Duval and Tweedie’s trim-and-fill method. However, Egger’s regression intercept was significant (Intercept = 2.565; *t* = 2.682; *p* = 0.015; two-tailed). The mean of effect sizes and forest plot is presented in Fig. 3e and a funnel plot is provided in the supplemental materials as Table S3e.
    i. **Verbal Memory**: There were fifteen studies which reported outcome data for verbal learning and memory (word list recall and story recall). The summary effects were large and significant (Hedges’ *g* observed = 0.758; 95% CI [0.382, 1.133]; *Z* = 3.956; *p* <  0.001). Calculation of the HKSJ adjustment was also significant (HKSJ point estimate adjustment SMD = 0.775; 95% CI [0.188, 1.362]; *t* = 2.833; *df* = 14; *p* = 0.013). Heterogeneity between studies was significant (*Q* = 95.811; *df* = 14; *p* = 0.000; *I* ^2^ = 85.388%; τ^2^ = 0.421). Visual inspection of the funnel plot was asymmetrical with two outliers (Balietti et al. 2016; Herrera et al. 2012). Egger’s regression intercept was significant suggestive of small-study effects (Intercept = 2.894; *t* = 2.256; *p* = 0.042; two-tailed). The overall effect size and forest plot for verbal memory is presented in Fig. 3f and a funnel plot is provided the supplemental materials as Fig. S3f.
    ii. **Non-Verbal Memory**: Five studies reported outcome data regarding non-verbal memory. The summary effect size was moderate and significant (Hedges’ *g* observed = 0.570; 95% CI [0.160, 0.980]; *Z* = 2.726; *p* = 0.006). However, the HKSJ adjustment failed to reach significance (HKSJ point estimate adjustment SMD = 0.593; 95% CI [−0.016, 1.202]; *t* = 2.705; *df* = 4; *p* = 0.054). Heterogeneity was not significant (*Q* = 5.082; *df* = 4; *p* = 0.279; *I* ^2^ = 21.292%; τ^2^ = 0.047). Visual inspection of the funnel plot was asymmetrical and Duval and Tweedie’s trim-and-fill method trimmed two studies generating a moderate-sized adjusted point estimate (Adjusted point estimate = 0.317; 95% CI [−0.158, 0.792]; *Q* = 12.965). Egger’s regression intercept was not significant (Intercept = 3.272; *t* = 1.701; *p* = 0.187; two-tailed). The effect sizes and forest plot for non-verbal memory is presented in Fig. 3g and a funnel plot is provided the supplemental materials as Fig. S3g.
  - **Executive Functions**: A total of thirteen studies reported outcome data regarding the effects of cognitive training on executive functions. The summary effect size was moderate and significant (Hedges’ *g* observed = 0.575; 95% CI [0.093, 1.056]; *Z* = 2.339; *p* = 0.019). However, the HKSJ calculation failed to demonstrate significance (HKSJ point estimate adjustment SMD = 0.585; 95% CI [− 0.262, 1.432]; *t* = 1.505; *df* = 12; *p* = 0.158). Values representative of heterogeneity were significant (*Q* = 126.404; *df* = 12; *p* = 0.000; *I* ^2^ = 90.507%; *τ* ^*2*^ = 0.669). Visual inspection of the funnel plot was asymmetrical and there were no adjustments with Duval and Tweedie’s trim-and-fill method. Egger’s regression intercept was not significant (Intercept = 2.718; *t* = 1.242; *p* = 0.240; two-tailed). The effect sizes and forest plot for executive functioning measures is presented in Fig. 3h and a funnel plot is provided the supplemental materials as Fig. S3h.
3. **Intervention Effects – By Training Type**: Please see Table 4 for a breakdown of effects by training including neuropsychological instruments, individual effect sizes, and overall effects.

**Table 4:** Effect sizes by intervention type: Effects, confidence intervals and prediction intervals

Cognitive domain	Study	Measure	n	g	Actual/ observed intervals		p	*Q*	*df* (*Q*)	p	*I* ^2^	τ^2^	τ	Prediction intervals	
					Lower (95%)	Upper (95%)								Lower (95%)	Upper (95%)
Cognitive stimulation															
	No study used interventions exclusively in this area		–	–	–	–	–	–	–	–	–	–	–	–	–
Restorative training															
	Barnes et al. (2009)	Combined	36	0.533	−0.101	1.166	0.099								
	Carretti (2013)	Combined	20	0.542	−0.318	1.402	0.217								
	Finn and McDonald (2011)	Combined	16	1.033	0.039	2.027	0.042								
	Finn and McDonald (2015)	Combined	24	0.125	−0.650	0.901	0.752								
	Gagnon (2012)	Combined	24	0.623	−0.118	1.364	0.099								
	Herrera et al. (2012)	Combined	22	3.293	1.934	4.652	0.000								
	Jean et al. (2010a)	Combined	20	0.285	−0.565	1.136	0.576								
	Valdes et al. (2012)	SOPT	195	−1.565	−1.887	−1.243	0.000								
		Random - Observed (k = 8)		0.541	−0.456	1.539	0.288	111.09	7	0.000	93.699	1.886	1.373	−3.0429	4.1249
		HKSJ Point Estimate Adjustment (SMD)		0.568	−0.555	1.691	0.271								
Compensatory training															
	Greenaway et al. (2012)	Combined	40	0.508	−0.109	1.126	0.107								
	Hampstead et al. (2012a)	Memory – Visual	21	1.312	0.399	2.225	0.005								
		Random - Observed (k = 2)		0.837	0.063	1.612	0.034	–	–	–	–	–	–	–	–
		HKSJ Point Estimate Adjustment (SMD)	–	–	–	–	–								
Multicomponent interventions															
	Balietti et al. (2016)	Combined	70	2.495	1.839	3.151	0.000								
	Barban et al. (2016)	Combined	106	0.097	−0.285	0.478	0.619								
	Buschert et al. (2011)	Combined	22	0.154	−0.719	1.027	0.730								
	Fiatarone Singh et al. (2014)	Combined	22	0.184	−0.388	0.756	0.528								
	Forster et al. (2011)	Combined	18	0.427	−0.564	1.417	0.399								
	Giuli et al. (2016)	Combined	97	0.511	0.108	0.915	0.013								
	Jeong et al. (2016)	Combined	147	0.084	−0.261	0.428	0.634								
	Lam et al. (2015)	Combined	276	0.074	−0.162	0.310	0.539								
	Mowszowski et al. (2014)	Combined	40	0.268	−0.364	0.900	0.406								
	Olchik et al. (2013)	Combined	30	0.371	−0.334	1.077	0.302								
	Polito et al. (2015)	Combined	44	0.063	−0.517	0.644	0.831								
	Rapp (2002)	Combined	16	0.566	−0.396	1.528	0.249								
	Rojas et al. (2013)	Combined	30	0.894	0.156	1.631	0.018								
	Schmitter-Edgecombe (2014)	Combined	46	0.275	−0.296	0.846	0.346								
	Tsolaki et al. (2011)	Combined	176	0.424	0.121	0.727	0.006								
	Vidovich et al. (2015)	Combined	155	0.146	−0.169	0.461	0.363								
		Random - Observed (k = 16)		0.398	0.164	0.631	0.001	55.511	15	0.000	72.978	0.146	−0.382	−0.4603	1.2563
		HKSJ Point Estimate Adjustment (SMD)		0.404	0.098	0.710	0.013

  - **Cognitive Stimulation**: There were no studies which exclusively employed cognitive stimulation as a method of intervention in the works examined.
  - **Restorative Cognitive Training**: Eight studies reported outcome data after using restorative training forms of intervention. The summary effect size was moderate and not significant (Hedges’ *g* observed = 0.541; 95% CI [−0.456, 1.539]; *Z* = 1.064; *p* = 0.288). Calculating the HKSJ adjustment to account for small number of studies was also non-significant (HKSJ point estimate adjustment SMD = 0.568; 95% CI [−0.555, 1.691]; *t* = 1.196; *df* = 7; *p* = 0.271). Indicators of heterogeneity were significant (Q = 111.092; *df* = 7; *p* = 0.000; *I* ^2^ = 93.699%; τ^2^ = 1.886). Visual inspection of the funnel plot was asymmetrical. There were no adjustments with Duval and Tweedie’s trim-and-fill method. Egger’s regression intercept was significant (Intercept = 8.356; *t* = 8.501; *p* = 0.000; two-tailed). As such, this would reflect the presence of heterogeneity, publication bias, and concerns due to small-study effects. The effect sizes and forest plot for restorative training is presented in Fig. 4a and a funnel plot is provided the supplemental materials as Fig. S4a.

**Fig. 4:**
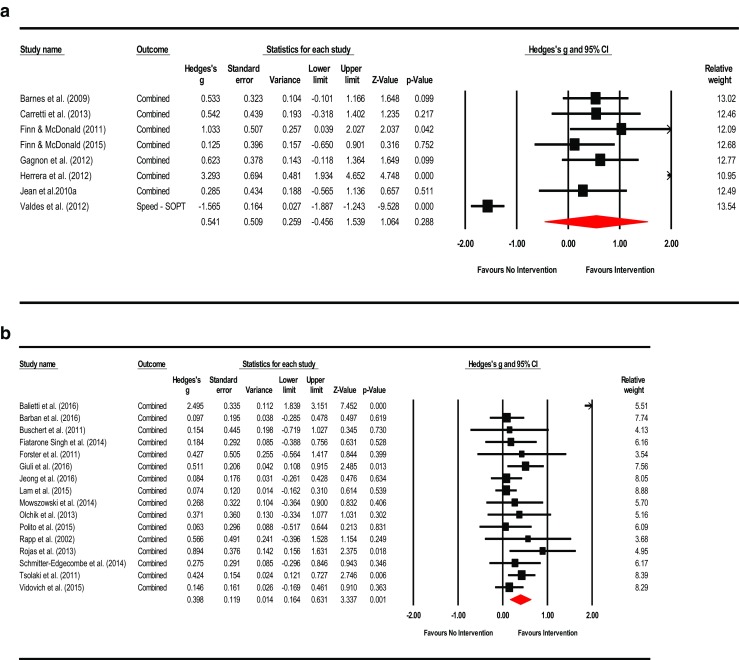
**a** Meta-analysis of restorative training on cognition (all outcomes). HKSJ point estimate adjustment SMD = 0.568; *t* = 1.196; *p* = 0.271. Test for heterogeneity *Q* = 111.092; *df* = 7; *p <*  0.001; *I* ^*2*^ = 93.699; *τ* ^*2*^ = 1.886. **b** Meta-analysis of multicomponent training on cognition (all outcomes). HKSJ point estimate adjustment SMD = 0.404; *t* = 2.810; *p* = 0.013. Test for heterogeneity *Q* = 55.511; *df* = 15; *p* < 0.001; *I* ^*2*^ = 72.978; *τ* ^*2*^ = 0.146
  - **Compensatory Cognitive Training**: Two studies reported using compensatory cognitive training as an intervention in their study. An analysis was not conducted for this form of training given the number of studies fell below minimal cutoff of five.
  - **Multicomponent Training**: Sixteen studies reported outcome data after using multicomponent training techniques. The summary effect size was moderate and significant (Hedges’ *g* observed = 0.398; 95% CI [0.164, 0.631]; *Z* = 3.337; *p* = 0.001). Calculating the HKSJ adjustment to account for the small number of studies was also significant (HKSJ point estimate adjustment SMD = 0.404; 95% CI [0.098, 0.710]; *t* = 2.810; *df* = 15; *p* = 0.013). Significant heterogeneity was observed (*Q* = 55.511; *df* = 15; *p* <  0.001; *I* ^2^ = 72.978%; τ^2^ = 0.146). Visual inspection of the funnel plot was asymmetrical with one prominent outlier (Bialetti et al. 2016). There were no adjustments with Duval and Tweedie’s trim-and-fill method. In addition, Egger’s regression intercept was not significant (Intercept = 1.819; *t* = 1.616; *p* = 0.128; two-tailed). While there was an indication of heterogeneity, significant point estimates in the context of relatively low values on bias indicators would favor there to be positive benefits from multicomponent training. The effect sizes and forest plot for multicomponent training is presented in Fig. 4b and a funnel plot is provided the supplemental materials as Fig. S4b. Sensitivity analysis examining effect sizes and measures of dispersion at various levels of correlation between test instruments demonstrated adequate mean effects and weights consistent with the values used a conservative estimate of combined outcome (Please see Table S8 in the supplementary materials).
4. **Interventions Effects – By Targeted Domain**: We also conducted meta-analyses by intervention method and its associated domain to examine the effects of interventions used on the cognitive outcome in which the intervention method was designed to target. Please see Table 5 for a breakdown of summary effects. Several domains were not examined due to the very small number or lack of studies which specifically targeted that domain (working memory *n* = 2, speed of information processing *n* = 2, language *n* = 0, visual-spatial ability *n* = 0, or executive functions *n* = 0). Additionally, while one study reported focusing primarily on memory, additional cognitive domains were included in their intervention methods and a composite score consisting of immediate and recognition paradigms was used to measure memory (Jeong et al. 2016). As such, this study was used only in the multidomain analysis.

**Table 5:** Intervention Effects by Content (cognitive domain targeted): Effect sizes, confidence intervals and prediction intervals

Domain targeted	Study	Measure	n	g	Actual/ observed intervals		p	*Q*	*df* (*Q*)	p	*I* ^2^	τ^2^	τ	Prediction intervals	
					Lower (95%)	Upper (95%)								Lower (95%)	Upper (95%)
Working memory/attention															
	Carretti (2013)	Combined	20	0.474	−0.383	1.331	0.279								
	Gagnon (2012)	Combined	24	0.893	0.173	1.613	0.015								
		Random - Observed (k = 2)		0.720	0.168	1.271	0.010	–	–	–	–	–	–	–	–
Speed of information processing															
	Barnes (2009)	*No outcome specifically for speed*		–	–	–	–								
	Valdes (2012)	SOPT	195	−1.565	−1.887	−1.243	0.000								
		Random - Observed (k = 1)		–	–	–	–	–	–	–	–	–	–	–	–
Language															
	*No interventions exclusively in this area*			–	–	–	–	–	–	–	–	–	–	–	–
Visual-spatial															
	*No interventions exclusively in this area*			–	–	–	–	–	–	–	–	–	–	–	–
Memory (verbal and non-verbal delay)															
	Balietti et al. (2016)	Combined	70	2.824	2.165	3.483	0.000								
	Finn and McDonald (2015)	VPA-II	24	0.182	−0.592	0.956	0.645								
	Hampstead et al. (2012a)	Memory – Visual (Percent Change)	21	1.312	0.399	2.225	0.005								
	Herrera et al. (2012)	Combined	22	3.158	1.778	4.537	0.000								
	Olchik et al. (2013)	RAVLT – Delay	30	0.487	−0.221	1.196	0.178								
	Rapp (2002)	Combined	16	0.645	−0.317	1.607	0.189								
	Schmitter-Edgecombe (2014)	Memory – Delay (RBANS V + NV)	46	0.293	−0.278	0.865	0.314								
		Random - Observed (k = 7)		1.219	0.338	2.100	0.007	51.777	6	0.000	88.412	1.219	1.104	−1.8453	4.2833
		HKSJ Point Estimate Adjustment (SMD)		1.099	0.008	2.190	0.049								
Executive functions															
	*No interventions exclusively in this area*			–	–	–	–	–	–	–	–	–	–	–	–
Multiple domain and lifestyle															
	Barban et al. (2016)	Combined	106	0.097	−0.285	0.478	0.619								
	Buschert et al. (2011)	Combined	22	0.154	−0.719	1.027	0.730								
	Fiatarone Singh et al. (2014)	Combined	22	0.184	−0.388	0.756	0.528								
	Finn and McDonald (2011)	Combined	16	0.427	−0.564	1.417	0.399								
	Forster et al. (2011)	Combined	18	0.427	−0.564	1.417	0.399								
	Giuli et al. (2016)	Combined	97	0.511	0.108	0.915	0.013								
	Jeong et al. (2016)	Combined	147	0.084	−0.261	0.428	0.634								
	Lam et al. (2015)	Combined	276	0.074	−0.162	0.310	0.539								
	Mowszowski et al. (2014)	Combined	40	0.268	−0.364	0.900	0.406								
	Polito et al. (2015)	Combined	44	0.063	−0.517	0.644	0.831								
	Rojas (2013)	Combined	30	0.894	0.156	1.631	0.018								
	Tsolaki (2011)	Combined	176	0.424	0.122	0.727	0.006								
	Vidovich (2015)	Combined	155	0.146	−0.169	0.461	0.363								
		Random - Observed (k = 13)		0.230	0.108	0.352	0.000	12.713	12	0.390	5.612	0.003	0.054	0.0475	0.4125
		HKSJ Point Estimate Adjustment (SMD)		0.232	0.094	0.370	0.003

  - **Memory**: Seven studies used mnemonic-focused forms of intervention and reported outcome data for verbal or non-verbal recall measures. While there were ten studies using memory focused interventions in total, one study did not administer outcomes specifically assessing memory (Greenaway et al. 2012), another study did not have sufficient data from primary outcomes to enter for analysis (Jean et al. 2010a), and one study used a memory composite score which would introduce unrelated non-delay data into the analysis, namely, immediate recall and recognition (Jeong et al. 2016). These studies were excluded from the analysis leaving a total of seven studies to examine. The summary effect of memory training on memory outcomes was large and significant (Hedges’ *g* observed = 1.219; 95% CI [0.338, 2.100]; *Z* = 2.713; *p* = 0.007). HKSJ adjustment for the small number of studies was also significant (HKSJ point estimate adjustment SMD = 1.099; 95% CI [0.008, 2.190]; *t* = 2.465; *df* = 6; *p* = 0.049). However, heterogeneity across studies was significant (*Q* = 51.777; *df* = 6; *p* = 0.000; *I* ^*2*^ = 88.412%; τ^2^ = 1.219). Visual inspection of the funnel plot was asymmetrical with two outliers (Balietti et al. 2016 and Herrera et al. 2012), although there were no adjustments after calculation of Duval and Tweedie’s trim-and-fill method. Egger’s regression intercept was not indicative of small-study effects (Intercept = 3.397; *t* = 0.709; *p* = 0.510; two-tailed). A forest plot of the effects and overall summary of memory strategies on memory outcomes is presented in Fig. 5a. A funnel plot is provided the supplemental materials as Fig. S5a.

**Fig. 5:**
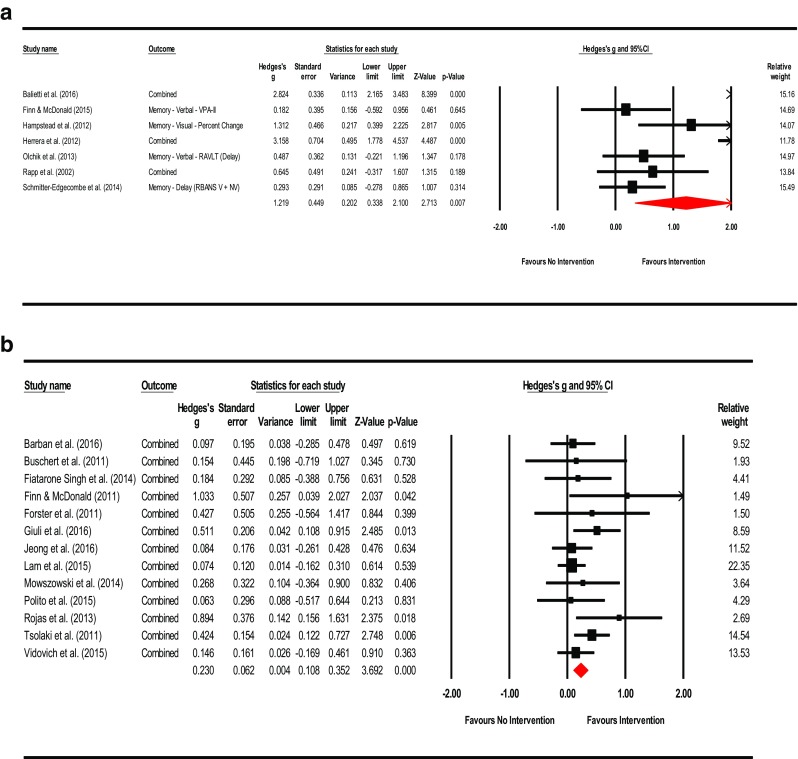
**a** Meta-analysis of interventions by targeted domain: memory (verbal + non-verbal combined). HKSJ point estimate adjustment SMD = 1.099; *t* = 2.465; *p* = 0.049. Test for heterogeneity *Q* = 51.777; *df* = 6; *p* = 0.000; *I* ^*2*^ = 88.412; *τ* ^*2*^ = 1.219. **b** Meta-analysis of interventions by targeted domain: multidomain. HKSJ point estimate adjustment SMD = 0.232; *t* = 3.667; *p* = 0.003. Test for heterogeneity *Q* = 12.713; *df* = 12; *p* = 0.390; *I* ^*2*^ = 5.612; *τ* ^*2*^ = 0.003
  - **Multiple Domain and Lifestyle**: A total of thirteen studies applied interventions which targeted multiple cognitive domains and other facets including lifestyle changes (i.e. education regarding meta-cognition, etc.). The summary effect was small and significant (Hedges’ *g* = 0.230; 95% CI [0.108, 0.352]; *Z* = 3.692; *p* = 0.000). Calculation of the HKSJ adjustment was also significant (HKSJ point estimate adjustment SMD = 0.232; 95% CI [0.094, 0.370]; *t* = 3.667; *df* = 12; *p* = 0.003). Heterogeneity indicators across studies was not significant (*Q* = 12.713; *df* = 12; *p* = 0.390; *I* ^2^ = 5.612%; τ^2^ = 0.003). With respect to possible publication bias, visual inspection of the funnel plot was slightly asymmetrical and Duval and Tweedie’s trim-and-fill method trimmed two studies generating a small adjusted point estimate (Adjusted point estimate = 0.205; 95% CI [0.056,0.350]; *Q* = 19.100). Egger’s regression intercept was not significant inferring a low likelihood of small-study effects (Intercept = 1.144; *t* = 1.795; *p* = 0.100; two-tailed). The significant point estimates and summary statistics would imply that interventions with content covering multiple cognitive domains may display improved performance on outcome measures (all included). As such, this finding would suggest individuals with MCI who receive interventions targeting multiple domains (including lifestyle changes) are apt to demonstrate a slight benefit on cognitive instruments. However, caution interpreting this finding is warranted due to the indication of some bias and inherently large number of intervention methods used and outcomes examined. While these may be statistically favorable findings, there may be other unknown factors unaccounted for by these analyses. Effect sizes, overall effect and forest plot for multidomain interventions on all outcomes (combined) is presented in Fig. 5b and a funnel plot is provided the supplemental materials as Fig. S5b.
5. **Influence of Moderator Variables**:
  - **Categorical Covariates**: A subgroup analysis was conducted with moderator variables of MCI diagnosis, mechanism of intervention (mode), type of training, intervention content, type of control, period of follow-up assessment, and control for repeat administration. The details concerning the findings for each covariate examined is provided in Table 6.

**Table 6:** Subgroup analysis: Examination of moderator variables and effect sizes on outcome measures across studies

Moderator	Subgroup	k	g	Actual/ observed intervals		Z	p	*Q*	df (*Q*)	p	*I* ^2^	τ^2^	τ	Sig.
				Lower (95%)	Upper (95%)									
MCI diagnosis														
Fixed-effect analysis														
	aMCI, single domain	5	0.570	0.205	0.935	3.060	0.002	3.535	4	0.473	0.000	0.000	0.000	
	aMCI, multiple domain	7	0.339	0.103	0.575	2.815	0.005	21.297	6	0.002	71.827	0.307	0.555	
	MCI, all	14	0.096	−0.016	0.208	1.684	0.092	107.524	13	0.000	92.376	0.583	0.764	
	Total Within							195.356	23	0.000				
	Total Between							8.250	2	0.016				
	Overall	26	0.171	0.074	0.269	3.445	0.001	203.606	25	0.000	87.721	0.479	0.692	
Mixed effects analysis														
	aMCI, single domain	5	0.585	−0.145	1.314	1.571	0.116							
	aMCI, multiple domain	7	0.647	0.040	1.254	2.089	0.037							
	MCI, all	14	0.329	−0.071	0.729	1.612	0.107							
	Total Between							0.887	2	0.642				ns
	Overall	26	0.453	0.149	0.756	2.893	0.003							
Mechanism of intervention (Mode)														
Fixed-effect analysis														
	Group	12	0.213	0.086	0.340	3.283	0.001	8.425	11	0.675	0.000	0.000	0.000	
	Individual	4	0.884	0.601	1.168	6.114	0.000	28.459	3	0.000	89.459	0.801	0.895	
	Computer	10	−0.200	−0.380	−0.020	−2.173	0.030	125.714	9	0.000	92.841	1.201	1.096	
	Total Within							162.599	23	0.000				
	Total Between							41.007	2	0.000				
	Overall	26	0.171	0.074	0.269	3.445	0.001	203.606	25	0.000	87.721	0.479	0.692	
Mixed effects analysis														
	Group	12	0.297	−0.116	0.710	1.411	0.158							
	Individual	4	1.008	0.293	1.723	2.762	0.006							
	Computer	10	0.394	−0.072	0.859	1.656	0.098							
	Total Between							2.918	2	0.232				ns
	Overall	26	0.445	0.161	0.728	3.073	0.002							
Training type (all outcomes)														
Fixed-effect analysis														
	Cognitive Stimulation	0	–	–	–	–	–	–		–	–	–	–	
	Restorative Training	8	−0.426	−0.650	−0.202	−3.732	0.000	109.189	7	0.000	93.589	1.847	1.359	
	Compensatory Training	3	0.505	0.123	0.886	2.594	0.009	4.211	2	0.122	52.507	0.134	0.365	
	Multicomponent	15	0.294	0.181	0.407	5.104	0.000	55.373	14	0.000	74.717	0.156	0.395	
	Total Within							167.441	23	0.000				
	Total Between							34.833	2	0.000				
	Overall	26	0.171	0.074	0.269	3.445	0.001	203.606	25	0.000	87.721	0.479	0.692	
Mixed effects analysis														
	Cognitive Stimulation	0	–	–	–	–	–							
	Restorative Training	8	0.389	−0.149	0.927	1.418	0.156							
	Compensatory Training	3	0.623	−0.224	1.470	1.441	0.150							
	Multicomponent	15	0.438	0.072	0.804	2.344	0.019							
	Total Between							0.211	2	0.900				*ns*
	Overall	26	0.445	0.160	0.730	3.061	0.002							
Primary domain targeted (all outcomes)														
Fixed-effect analysis														
	Attention & Concentration	2	0.589	0.027	1.150	2.055	0.040	0.020	1	0.889	0.000	0.000	0.000	
	Speed of Information Processing	2	−1.134	−1.421	−0.847	−7.748	0.000	33.460	1	0.000	97.011	2.134	1.461	
	Language	0	–	–	–	–	–	–		–	–	–	–	
	Visual-Spatial Ability	0	–	–	–	–	–	–		–	–	–	–	
	Memory	8	0.549	0.278	0.820	3.968	0.000	19.315	7	0.007	63.759	0.278	0.527	
	Executive Functions	0	–	–	–	–	–	–		–	–	–	–	
	Multiple (Cognition, lifestyle, etc.)	14	0.294	0.180	0.408	5.040	0.000	57.279	13	0.000	77.304	0.173	0.415	
	Total Within							110.074	22	0.000				
	Total Between							93.532	3	0.000				
	Overall	26	0.171	0.074	0.269	3.445	0.001	203.606	25	0.000	87.721	0.479	0.692	
Mixed effects analysis														
	Attention & Concentration	2	0.585	−0.334	1.504	1.247	0.212							
	Speed of Information Processing	2	−0.636	−1.438	0.166	−1.554	0.120							
	Language	0	–	–	–	–	–							
	Visual-Spatial Ability	0	–	–	–	–	–							
	Memory	8	0.671	0.207	1.136	2.832	0.005							
	Executive Functions	0	–	–	–	–	–							
	Multiple (Cognition, lifestyle, etc.)	14	0.449	0.135	0.763	2.801	0.005							
	Total Between							7.942	3	0.047				*
	Overall	26	0.420	0.182	0.659	3.449	0.001							
Type of control group														
Fixed-effect analysis														
	Passive	15	0.091	−0.022	0.204	1.575	0.115	172.301	14	0.000	91.875	0.596	0.772	
	Active	11	0.406	0.213	0.599	4.128	0.000	23.655	10	0.009	57.726	0.163	0.403	
	Total Within							195.957	24	0.000				
	Total Between							7.649	1	0.006				
	Overall	26	0.171	0.074	0.269	3.445	0.001	203.606	25	0.000	87.721	0.479	0.692	
Mixed effects Analysis														
	Passive	15	0.341	−0.046	0.727	1.729	0.084							
	Active	11	0.621	0.143	1.100	2.546	0.011							
	Total Between							0.799	1	0.371				*ns*
	Overall	26	0.452	0.151	0.752	2.945	0.003							
Follow-up assessment: period post-intervention														
Fixed-effect analysis														
	Within 2-weeks	21	0.310	0.198	0.421	5.444	0.000	73.511	20	0.000	72.793	0.193	0.439	
	More than 2-weeks	5	−0.274	−0.475	−0.074	−2.689	0.007	105.089	4	0.000	96.194	1.622	1.274	
	Total Within							178.600	24	0.000				
	Total Between							25.006	1	0.000				
	Overall	26	0.171	0.074	0.269	3.445	0.001	203.606	25	0.000	87.721	0.479	0.692	
Mixed effects analysis														
	Within 2-weeks	21	0.515	0.190	0.840	3.109	0.002							
	More than 2-weeks	5	0.173	−0.485	0.830	0.514	0.607							
	Total Between							0.838	1	0.360				*ns*
	Overall	26	0.448	0.157	0.739	3.016	0.003							
Control for repeat administration														
Fixed-effect analysis														
	No	17	0.077	−0.036	0.191	1.339	0.181	170.271	16	0.000	90.603	0.584	0.764	
	Yes	9	0.437	0.246	0.628	4.492	0.000	23.236	8	0.003	65.570	0.183	0.428	
	Total Within							193.507	24	0.000				
	Total Between							10.099	1	0.001				
	Overall	26	0.171	0.074	0.269	3.445	0.001	203.606	25	0.000	87.721	0.479	0.692	
Mixed effects analysis														
	No	17	0.335	−0.031	0.702	1.793	0.073							
	Yes	9	0.685	0.165	1.205	2.581	0.010							
	Total Between							1.160	1	0.281				*ns*
	Overall	26	0.451	0.152	0.751	2.952	0.003

    i. **MCI Diagnosis**: With respect to effect sizes for individual diagnostic categories, there was a significant effect for aMCI multiple domain (Hedges’ *g* = 0.647; *p* = 0.037) and a non-significant effect for aMCI single domain (Hedges’ *g* = 0.585; *p* = 0.116) and MCI-all (Hedges’ *g* = 0.329; *p* = 0.107). However, the overall test comparing diagnostic types would indicate there to be no difference in effects by MCI diagnostic category (Total Between *Q* = 0.887; *df* = 2; *p* = 0.642).
    ii. **Mechanism of Intervention (Mode)**: The overall test exploring the mode of intervention was not significant. While the individual point estimate for an individual approach was significant (Hedges’ *g* = 1.008; *p* = 0.006), the overall test examining mode of intervention did not reach significance (Total Between *Q* = 2.918; *df* = 2; *p* = 0.232).
    iii. **Training Type**: The overall test comparing training type found no difference in the effectiveness of interventions based on type of training applied (Total Between *Q* = 0.211; *df* = 2; *p* = 0.900). Individual point estimates for multicomponent interventions were significant (Hedges’ *g* = 0.438; *p* = 0.019) while restorative and compensatory types of training were unremarkable (Hedges’ *g* = 0.389; *p* = 0.156 and Hedges’ *g* = 0.623; *p* = 0.150, respectively). There were no studies which exclusively used cognitive stimulation in the group of studies examined.
    iv. **Intervention Content**: The overall significance for domains targeted by the intervention was significant (Total Between *Q* = 7.942; *df* = 3; *p* = 0.047). Individual point estimates were also significant for memory (Hedges’ *g* = 0.671; *p* = 0.005) and multidomain (Hedges’ *g* = 0.449; *p* = 0.005) forms of content. This would suggest that interventions which focused on memory and multiple domains had a significant influence on outcomes (all cognitive measures combined). The larger effect size observed for memory would further suggest that interventions with memory content may be more effective than interventions with multidomain content. Of note, the lack of significance in other areas is likely due to very low number of studies (or no studies) and would not be regarded to be an indicator of ineffectiveness.
    v. **Type of Control (Passive v. Active)**: The overall test comparing passive versus active forms of control groups was not significant (Total Between *Q* = 0.799; *df* = 1; *p* = 0.371). This would suggest the type of control group did not have an impact on the outcomes observed.
    vi. **Period of Post-Intervention Assessment**: The overall test comparing period of follow-up assessment post-intervention was not significant (Total Between Q = 0.838; *df* = 1; *p* = 0.360). This would suggest the period of post-assessment, within 2-weeks versus more than 2-weeks, did not appear to be a significant factor on the outcomes reported in the studies examined.
    vii. **Control for Repeat Administration**: Examining control for repeat test administration was not significant (Total Between *Q* = 1.160; *df* = 1; *p* = 0.281). This would imply that methods employed to counter test-retest effects such as alternate or parallel test versions were unlikely to have had an influence on measurement outcome for the instruments used.
  - **Covariates Combined**: Twenty-five studies were included in the meta-regression analysis, one study could not be included as the duration of training (in hours) was not reported (Finn and MacDonald, 2015). A meta-regression analysis testing the model duration of training and type of training regressed on Hedges’ *g* was not significant. This would suggest duration of training and type of training was not related to effect size (*k* = 25; *Q* = 0.15; *df* = 3; *p* = 0.9848). Test for Goodness of Fit was also significant further indicating tht the model was also not predictive of an effect (τ^2^ = 0.4862; τ = 0.6973; *I* ^2^ = 87.38%; *Q* = 166.44; *df* = 21; *p* < 0.00). Test for between-study variance was not significant (*τ* ^*2*^ = 0.4903; *τ* = 0.7002; *I* ^2^ = 88.21%; *Q* = 203.59; *df* = 24; *p* = 0.0000; *R* ^*2*^ analog = 0.01). Examining increments at each step of the model, none of the covariates entered were significant. Please see Table 7 for a print out of the main results and Fig. 6 for a scatterplot of the regression of Hedges’ g on training duration.

**Table 7:**
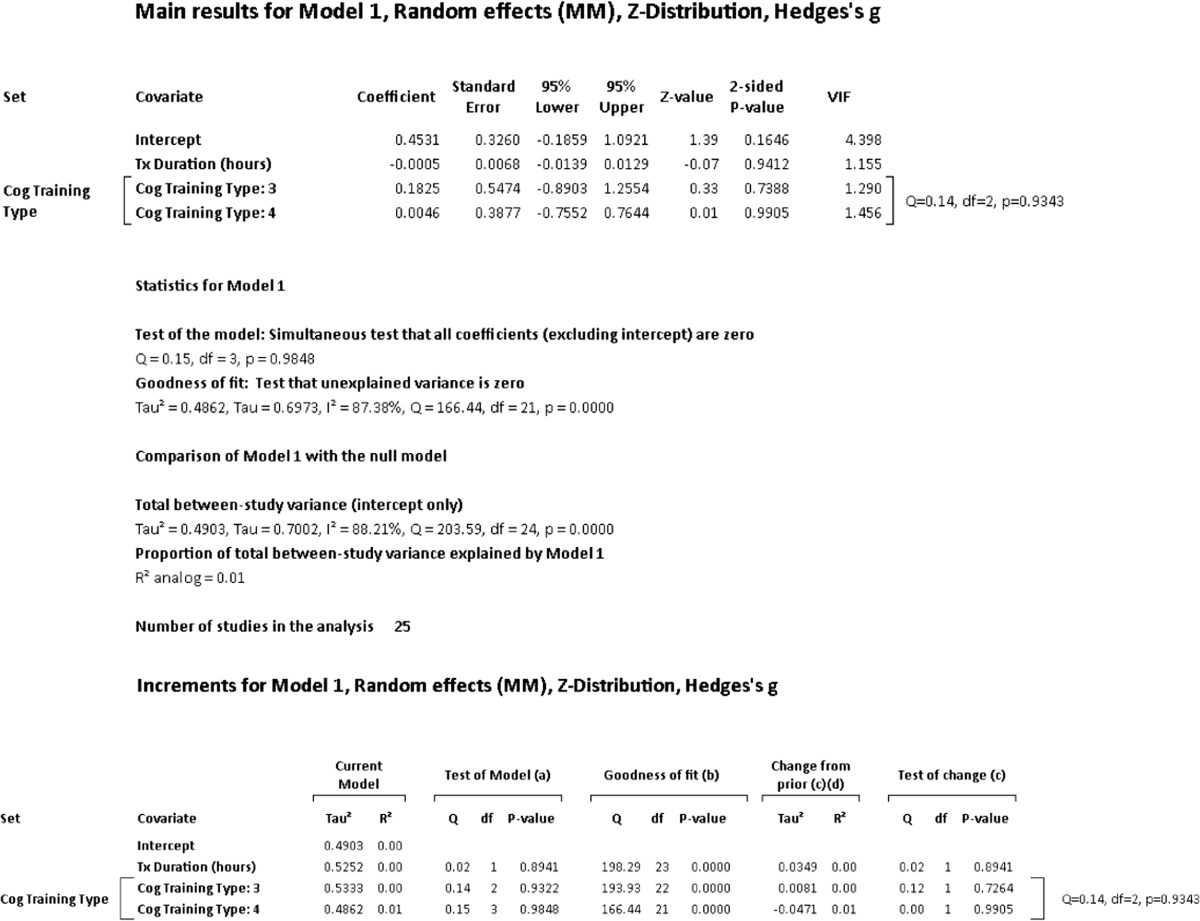
Meta-regression of duration of intervention and type of cognitive training: main results and increments

**Fig. 6:**
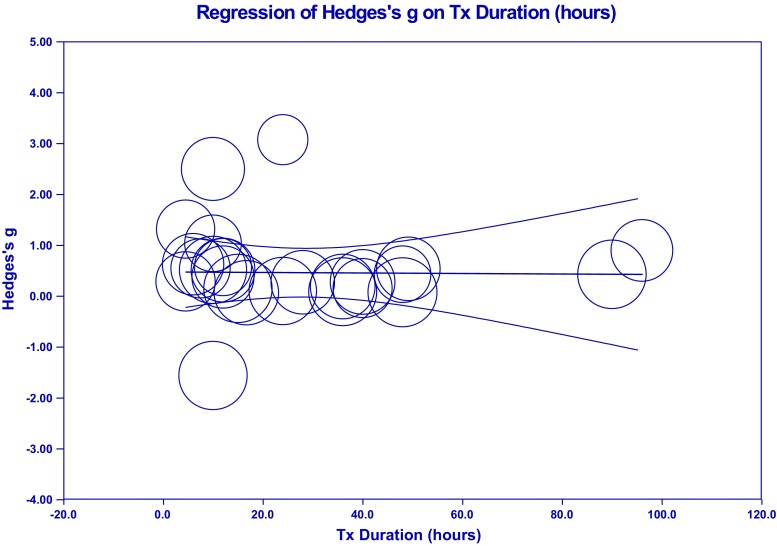
Scatterplot of regression of Hedges’ g on training duration
6. **Post Hoc** ***Analyses***: An additional hierarchical regression was also conducted using three moderators, control for repeat administration, duration of training (total length of time), and type of cognitive training, to explore how the combination of these may be associated with the effect of intervention outcomes. The model was not significant (*k* = 25; *Q* = 1.37; *df* = 4; *p* = 0.8487. Test for Goodness of Fit was significant indicating the model was not fully predictive of the entire effect (τ^2^ = 0.4651; τ = 0.6820; *I* ^2^ = 86.55%; *Q* = 148.73; *df* = 20; *p* = 0.0000). Test for between-study variance was significant with the covariates explaining 5% of the variance (τ^2^ = 0.4903; τ = 0.7002; *I* ^2^ = 88.21%; *Q* = 203.59; *df* = 24; *p* = 0.0000; *R* ^*2*^ analog = 0.05). Examining increments and test of change at each step of the model was not significant for control for repeat administration (Step 1 Test of Model *Q* = 1.04; *df* = 1; *p* = 0.3068), duration of intervention (Step 2 = *Q* = 0.01; *df* = 1; *p* = 0.9123) or type of training (Step 3 = *Q* = 0.27; *df* = 2; *p* = 0.8750). Please see Table 8 for a print out of the main results and details for incremental steps in the analysis.

**Table 8:**
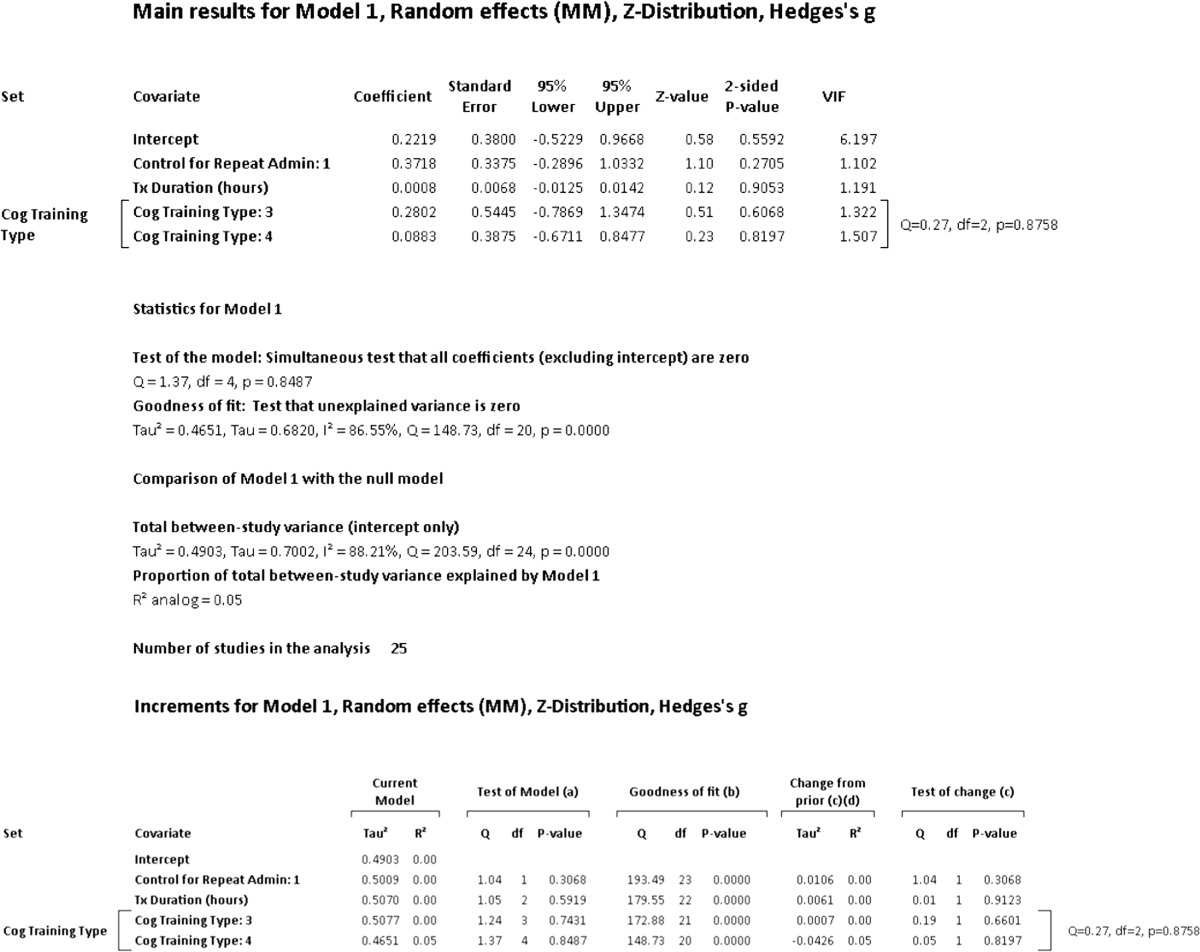
Post hoc analysis: meta-regression of control for repeat administration, duration of intervention and type of cognitive training: main results and increments

## Discussion

Results from these meta-analyses provide new information regarding the efficacy of cognitive interventions and training in MCI and expand existing literature in several important ways. First, we found significant, moderate effects for multicomponent training (Hedges’ *g* observed = 0.398; 95% CI [0.164, 0.631]; *Z* = 3.337; *p* = 0.001; *Q* = 55.511; *df* = 15; *p* <  0.001; *I* ^*2*^ = 72.978%; *τ* ^*2*^ = 0.146) as well as multidomain focused strategies (Hedges’ *g* = 0.230; 95% CI [0.108, 0.352]; Z = 3.692; p < .001; *Q* = 12.713; *df* = 12; *p* = 0.390; *I* ^*2*^ = 5.612; *τ* ^*2*^ = 0.003). These results suggest that individuals with MCI who received multicomponent forms of training or used interventions which targeted multiple cognitive domains (including lifestyle changes) also demonstrated small – moderate improvements on measures of cognition post-intervention. This is consistent with other reviews which found support for multidomain forms of intervention (Ballesteros et al. 2015; Bamidis et al. 2014; Li et al. 2011, 2014, 2017; Maffei et al. 2017; Suo et al. 2016; Yin et al. 2014). Regarding the other interventions examined, there was insufficient evidence to offer greater clarification of effects by cognitive domain or type of training. This is also consistent with prior reports which found modest improvements after training and interpretive complications associated with methodological and technical factors (Belleville 2008; Gates et al. 2011; Huckans et al. 2013; Kruz et al., 2011; Stott and Spector 2011; Vidovich and Almeida 2011). Caution interpreting some findings is warranted and, in some instances, precludes interpretation due to heterogeneity arising from the number of strategies employed, measures used to quantify outcome, other differences in methodology, and diversity of settings.

Second, subgroup examination of the moderator effects associated with intervention content revealed a significant overall effect (Total Between *Q* = 7.492; *df* = 3; *p* = 0.047) with significant point estimates for memory-based (Hedges’ *g* = 0.671; *p* = 0.005) and multidomain forms of content (Hedges’ *g* = 0.449; *p* = 0.005). This would suggest that, in the MCI populations within the studies examined, interventions which were memory- or multidomain-based had sufficient influence on training to have made an appreciable difference on test performance post-intervention, with memory-based content possibly being more effective than multidomain methods. Of note, however, there was an insufficient number of studies in other domains to provide a meaningful investigation of other forms of content. In addition, results regarding the influence of moderator variables would be regarded as observational only and not the equivalent of conducting direct comparisons of each approach in well-designed RCTs. As such, cautions over interpreting these findings are warranted.

Third, apart from intervention content, the subgroup analyses and meta-regressions performed were not significant for any of the other categorical moderators examined. The lack of significance of these covariates would suggest that MCI diagnostic type, training type, mode of intervention, type of control, post-intervention assessment period, or control for repeat administration did not have an appreciable influence on subjects’ performance on outcome measures. This would imply that the structural elements of intervention programs and the mechanism of instruction (i.e. group vs. computer, etc.) may be less of a factor than the content of the interventions applied. The finding that duration of intervention (number of hours) had little influence on magnitude of outcomes in both meta-regression models is consistent with other reports which did not find a dose-response relationship between length of training and outcome benefit. As such, the duration of training may have little or no effect on intervention outcome and shorter sessions may be associated with larger treatment gains in older adults (Verhaeghen et al. 1992). In addition, there were no moderator effects observed for repeat test administration; the use of MCI controls under RCT conditions is likely to mitigate concerns for practice effects. This is further underscored by reports which found nominal consequences of test-retest administration in older adults (Mitrushina and Satz 1991).

Taken together, the moderate summary effects observed after multicomponent and multidomain interventions would be consistent with activation of compensatory mechanisms described by the STAC-r model such that multifaceted cognitive interventions may recruit alternate networks as neurocomputational support to aid primary functional networks process load demands (Barban et al. 2017; Ciarmiello et al. 2015; Onur et al. 2016). As such, strategies which target both primary functional networks as well as alternate pathways simultaneously may be the most efficacious form of intervention for individuals with MCI. These types of interventions may challenge primary and alternate networks together, prompting neuroplastic reorganization. However, training results in small to moderate effects due to decreased functionality and efficiency of primary networks  and limitations of alternate networks to fully compensate processing needs. The summary effect observed with multicomponent and multidomain forms of intervention may reflect this process. Individuals with MCI may, by definition, demonstrate less of a benefit from domain-targeted interventions due to loss of neural structure and function and better outcome with multidomain forms of intervention. While there are a limited number of RCT studies which specifically outline the neurocognitive pathways active after cognitive training with neuroimaging tools, this intepretation would be consistent with increased cerebral blood flow in parahippocampal areas and maintenance of neural efficiency after multicomponent training in MCI (Maffei et al. 2017).

Moreover, the positive benefit of strategies with memory-based content may represent some transfer effects to other domains. Although the creation of new primary network paths appears limited in MCI, interventions with memory-based content may facilitate partial activation of neuroplastic reorganization and compensatory processes in several areas (Hampstead et al. 2011; Hampstead et al. 2012a; Rosen et al. 2011). The positive benefit of memory-based strategies may also be indicative of the complex interrelated pathways involved in memory (Belleville et al. 2011). While inconclusive due to study limitations, the relative absence of significant effects of training by cognitive domain or domain-specific interventions in our data may reflect the loss of primary network efficiency and limited activation of compensatory mechanisms. However, this question could not be explored further due to the limited number of studies, the presence of heterogeneity, and lack of data interpretability regarding other interventions (i.e. restorative training). Similarly, mechanisms reflective of cognitive reserve could not be fully examined due to lack of data (i.e. premorbid IQ, level of education, etc.). The neurocognitive conclusions drawn from our analyses and offered here would be regarded as speculative only and require comprehensive examination of intervention methods with the appropriate neuroimaging tools in well-designed RCTs.

With respect to the specific questions we sought to answer,

1. *What were the changes in cognition from baseline to outcome after the intervention was applied?* Improvements in cognition were, generally, observed with multicomponent types of training. Benefits were also observed with multidomain and lifestyle approaches, although this finding is qualified due to possibility of bias, inherently large number of intervention methods applied, and large number of outcomes examined.
2. *What were the common characteristics found to be effective across studies?* There was a positive influence associated with interventions using memory and multidomain-lifestyle forms of approaches, as examined through moderator variable and subgroup analyses.
3. *What are specific interventions that may be applied to individuals diagnosed with MCI in the clinical setting?* Multicomponent types of cognitive training appear to improve performance on cognitive outcomes for individuals with MCI. Significant point estimates in the context of relatively low values on bias indicators infer there was a positive benefit from multicomponent training, although moderate heterogeneity due to the range of strategies, number of outcomes and various settings was present. The effects of cognitive stimulation, restorative training, and compensatory training were indeterminate due to heterogeneity, publication bias and the limited number of studies.
4. *What are the key structural factors needed to set up an effective MCI intervention program (*e.g. *duration, group format,* etc.*)?* With the exception of intervention content noted above, we found no significant influence for MCI diagnosis, training type, mode of intervention, type of control, post-intervention follow-up assessment period, or control for repeat administration on outcome measures. However, these findings should be interpreted with caution and regarded to be observational only as this would not be the equivalent of conducting specific RCT studies which directly compare each characteristic of training.
5. *What inferences may be made regarding interventions applied and the neural processes involved in MCI?* Multicomponent and multidomain forms of intervention may facilitate recruitment of alternate neural processes as well as supporting primary networks to meet task demands simultaneously, resulting in small to moderate training effects. Interventions with memory-based or multidomain forms of content may, specifically, facilitate some activation of compensatory scaffolding and neuroplastic reorganization, although the creation of new primary network paths may be limited due to the neuropathological processes associated with MCI.

### Strengths & Limitations

Strengths of this review were to limit study inclusion to RCTs with individuals who met strict MCI diagnostic guidelines and to have measured cognitive performance with well-established standard neuropsychological instruments. Improvements in establishing clearer diagnostic criteria for the types of MCI and implementing this into RCTs has contributed significantly to understanding effects of cognitive training in MCI. In addition, the increased number of RCTs in MCI conducted over the past several years lends credence to the conclusions drawn. To our knowledge, this is the largest review of RCTs in MCI with MCI controls to date (*k* = 26), 92% of studies examined were published within the past seven years. The number of countries represented (*n* = 11) including a multicenter group of nations was also regarded as a strength, although the technical factors associated with this level of diversity also represents a challenge.

The limitations of this study are similar to other meta-analyses in MCI. There were generally small sample sizes, a range of interventions employed, diversity of sites, heterogeneity, publication bias and, while greatly improved in our view, a diverse number of instruments used to measure outcome. As such, there was insufficient data to conduct a complete evaluation of all interventions applied. Of greatest concern were the limited number of studies in individual cells, various interventions (some overlapping), types of training, heterogeneous number of outcomes (low precision), and modest number of active controls. A substantial amount of heterogeneity may have occurred secondary to the number of settings and variability in measurement precision. The studies examined were conducted across multiple countries with instruments which appeared to be similar, prima facie*,* but may have substantially differed in their psychometric properties. Evidence of this may be seen in effects of interventions on language, some heterogeneity was observed with an almost exclusive use of one measure of semantic fluency across studies.

Other limitations include the small number of studies in each diagnostic category, absence of measurement of premorbid IQ, unknown date of illness onset or character of course, minimal use of measures of adherence, and design variation between studies (Hampstead et al. 2014). The limited number of studies in each diagnostic category is problematic as MCI subtypes may demonstrate distinct connectivity patterns (Jacquemont et al. 2017). Improved characterization of various factors associated with baseline status and other covariates would also have aided comparability across studies. This has implications for our findings and a potential confound, the shifting deficits across illness and the progressive nature of MCI may require ‘evolving-strategies’ which complement and keep pace with patient cognitive decline to demonstrate a clinical benefit. Additionally, the lack of a unified method of reporting data and clear structure to communicate this information hinders accessibility to the results provided in published works.

In summary, findings from these meta-analyses suggest individuals with MCI who received multicomponent forms of training or used interventions which targeted multiple cognitive domains (including lifestyle changes) also demonstrated small to moderate improvements in cognition post-intervention. As such, multicomponent and multidomain forms of intervention may prompt recruitment of alternate neural processes as well as support primary networks to meet task demands simultaneously. In addition, interventions with memory and multidomain forms of content appear to be particularly helpful, with memory-based approaches possibly being more effective than multidomain methods. Otherwise, the effects of interventions by cognitive domain, training type, or other targeted domains (content) did not achieve statistical significance or were not significant. Given this pattern of results, although the creation of new primary network paths may be limited in MCI, interventions with memory or multidomain forms of content may facilitate partial activation of compensatory scaffolding and neuroplastic reorganization. The positive benefit of memory-based strategies may also reflect transfer effects indicative of compensatory network activation and the multiple-pathways involved in memory processes. Cautions interpreting these findings is warranted, however, due to heterogeneity, probability of publication bias, and small number of studies across cognitive domains. Significant amounts of variability and bias were present precluding more a definitive interpretation of the outcomes observed.

## Electronic supplementary material

Figure S2 – Funnel plot of Standard Error by Hedges’ *g* for effect of cognitive interventions on all outcome measures (DOCX 18 kb)

Figure S3a – Funnel plot of Standard Error by Hedges’ *g* for effect of cognitive interventions on mental status and general cognition (DOCX 17 kb)

Figure S3b – Funnel plot of Standard Error by Hedges’ *g* for effect of cognitive interventions on working memory and attention (DOCX 17 kb)

Figure S3c – Funnel plot of Standard Error by Hedges’ *g* for effect of cognitive interventions on speed of information processing (DOCX 17 kb)

Figure S3d – Funnel plot of Standard Error by Hedges’ *g* for effect of cognitive interventions on language (DOCX 17 kb)

Figure S3e – Funnel plot of Standard Error by Hedges’ *g* for effect of cognitive interventions on memory: verbal and nonverbal combined (DOCX 17 kb)

Figure S3f – Funnel plot of Standard Error by Hedges’ *g* for effect of cognitive interventions on memory: verbal (DOCX 17 kb)

Figure S3g – Funnel plot of Standard Error by Hedges’ *g* for effect of cognitive interventions on memory: nonverbal (DOCX 17 kb)

Figure S3h – Funnel plot of Standard Error by Hedges’ *g* for effect of cognitive interventions on executive functions (DOCX 18 kb)

Figure S4a – Funnel plot of Standard Error by Hedges’ *g* for effects of restorative training (DOCX 17 kb)

Figure S4b – Funnel plot of Standard Error by Hedges’ *g* for effects of multicomponent training (DOCX 17 kb)

Figure S5a – Funnel plot of Standard Error by Hedges’ *g* for effects of targeted outcome: Memory (DOCX 17 kb)

Figure S5b – Funnel plot of Standard Error by Hedges’ *g* for effects of targeted outcome: Multidomain (DOCX 18 kb)

Table S1 – Terms and Definitions (DOCX 14 kb)

Table S2 – PRISMA Checklist (DOCX 28 kb)

Table S3 – Boolean Search Strategy, Terms, and Results (DOCX 23 kb)

Table S4 – List of Abbreviations (DOCX 16 kb)

Table S5 – List of outcome measures by domain (DOCX 18 kb)

Table S6 – Summary of overall sample characteristics and length of interventions (DOCX 23 kb)

Table S7 – Effect sizes, confidence intervals and *p*-values for individual outcomes by study (assumes independence) (DOCX 42 kb)

Table S8 – Sensitivity Analysis: Effect sizes and measures of dispersion when combining outcomes at various levels of correlation between test instruments (DOCX 17 kb)
