# Supplementary material for: The Efficacy of Cognitive Intervention in Mild Cognitive Impairment (MCI): a Meta-Analysis of Outcomes on Neuropsychological Measures
Source: Neuropsychol Rev. 2017 Dec 27;27(4):440–84. doi: 10.1007/s11065-017-9363-3 (PMC5754430; doi:10.1007/s11065-017-9363-3)
Supplement: Supplementary file 10 — – Funnel plot of Standard Error by Hedges’ g for effects of restorative training (DOCX 17 kb) [file 11065_2017_9363_MOESM10_ESM.docx]

Figure S4a

*Funnel plot of Standard Error (SE) by Hedges’ g for the effect of restorative training - all outcome measures (observed and imputed)*
