# Supplementary material for: The Efficacy of Cognitive Intervention in Mild Cognitive Impairment (MCI): a Meta-Analysis of Outcomes on Neuropsychological Measures
Source: Neuropsychol Rev. 2017 Dec 27;27(4):440–84. doi: 10.1007/s11065-017-9363-3 (PMC5754430; doi:10.1007/s11065-017-9363-3)
Supplement: Supplementary file 14 — – Terms and Definitions (DOCX 14 kb) [file 11065_2017_9363_MOESM14_ESM.docx]

Table S1

*Terms and definitions*

| **Term** | **Definition** |
| --- | --- |
| Restorative Strategies | Methods of training which target specific cognitive areas with the goal of returning the deficit to premorbid levels. These types of training methods include errorless learning, spaced retrieval, vanishing cues, reality orientation therapy, and reminiscence therapy. |
| Compensatory Strategies | Methods of training which complement or supplement existing abilities in an effort maximize remaining skill sets. This may include visual imagery, method of loci, mind mapping, categorization and organization, face-name associations, chunking, cueing, and memory aids such as logs, notebooks, and calendars. |
| Cognitive Stimulation | Nonspecific activities used to increase cognitive and social functioning (i.e. discussion of a topic) or leisure-based interventions used to increase cognitive engagement (i.e. board-games or computer-based activities). |
| Cognitive Intervention | General reference to nonspecific actions applied to facilitate thinking skills (i.e. memory training, repetition, etc.). |
| Cognitive Rehabilitation | Typically refers to an individualized approach to remediation in which goals are identified for the patient and targeted in a systematic manner to treat deficits resulting from illness or injury. Cognitive rehabilitation has also been used to refer to lifestyle changes such as activities to increase protective factors (i.e. exercise, nutrition, sleep, etc.) or interventions to decrease negative, high risk behaviors (i.e. smoking). |
| Computer Training | Training methods which are software based administered paradigms targeting specific cognitive functions or multidomain processes. These may be administered through online access or applications on mobile devices, laptops, or desktop systems. |
| Education-based or psychoeducation approaches | Interventions which are instructional in nature and serve to explain how the brain functions or to describe the details of other processes (i.e. types of memory and how the brain stores information in memory). |
| Lifestyle interventions | Wholistic approaches in which cognitive training is an element of a larger multifaceted program designed to increase protective health behaviors (i.e. diet, nutrition and exercise), facilitate engagement in social activities, reduce high-risk negative behaviors, and monitor key aspects of health (Kivipelto et al. 2013). |
| Multicomponent or Multimodal training | Typically refers to combining several training approaches in a treatment program |
| Trained Effect | Direct effects on outcome measures are observed in areas specifically targeted by cognitive training. |
| Near-Transfer Effect | Effects seen from training are observed on similar outcome measures in the same cognitive domain which may not have been targeted by training. |
| Far-Transfer Effect | Effects of the intervention are observed on non-trained domains of cognition. |

Explanation:

Multiple types of interventions and a heterogenous array of terms have been used ranging from nonspecific group-based activities to rigorously structured interventions which target highly focused cognitive abilities at the individual level (Bahar-Fuchs, Clare & Woods, 2013; Belleville, 2008; Hampstead, Gillis, & Stringer, 2014; Huckans, Hutson, Twamley, Jak, Kaye, & Storzbach, 2013; Jean, Bergeron, Thivierge, & Simard, 2010; Sitzer et al, 2006; Stott & Spector, 2011). Establishing an operational definition and delineating these into functional tasks (Gates & Venezuela, 2010; Kinsella et al., 2009; Martin, Clare, Altgassen, Cameron, & Zehnder, 2011; Simon, Yokomizo, & Bottino, 2012), cognitive training generally refers to two categories of nonpharmacological interventions used to increase cognitive functions: restorative strategies and compensatory strategies. Restorative strategies target specific cognitive areas with the goal of returning the deficit to premorbid levels. These types of training methods include errorless learning, spaced retrieval, vanishing cues, reality orientation therapy, and reminiscence therapy. Compensatory strategies refer to new methods which complement or supplement existing abilities in an effort maximize remaining skill sets. This may include visual imagery, method of loci, mind mapping, categorization and organization, face-name associations, chunking, cueing, and memory aids such as logs, notebooks, and calendars.

Other terms which have been used to describe cognitive interventions include cognitive stimulation, general cognitive interventions, cognitive rehabilitation, computer administered training programs, education, lifestyle approaches, as well as other instrumental activities (i.e. exercise, music, etc.). Cognitive stimulation refers to nonspecific activities used to increase cognitive and social functioning (i.e. discussion of a topic) or leisure-based interventions used to increase cognitive engagement (i.e. board-games or computer-based activities). Similarly, cognitive intervention is a general reference to nonspecific actions applied to facilitate thinking skills (i.e. memory training, repetition, etc.). Cognitive rehabilitation is typically used to refer to an individualized approach to remediation in which goals are identified for the patient and targeted in a systematic manner to treat deficits resulting from illness or injury. Cognitive rehabilitation has also been used to refer to lifestyle changes such as activities to increase protective factors (i.e. exercise, nutrition, sleep, etc.) or interventions to decrease negative, high risk behaviors (i.e. smoking). Computer training methods are software administered paradigms targeting specific cognitive functions or multidomain processes. These may be administered through online access or applications on mobile devices, laptops, or desktop systems. Education-based or psychoeducational approaches are instructional in nature and serve to explain how the brain functions or to describe the details of other processes (i.e. types of memory and how the brain stores information in memory). Lifestyle interventions are wholistic approaches in which cognitive training is an element of a larger multifaceted program designed to increase protective health behaviors (i.e. diet, nutrition and exercise), facilitate engagement in social activities, reduce high-risk negative behaviors, and monitor key aspects of health (Kivipelto et al. 2013). Lastly, multicomponent or multimodal training typically refers to combining several training approaches in a treatment program. For the purposes of this review, we have adopted the following nosology: (1) intervention as a broad-based idiom to refer, generally, to any effort employed; (2) cognitive stimulation to mean nonspecific and leisure forms of activities; (3) cognitive training to denote either compensatory or restorative forms of training (based on the definitions above); and (3), multicomponent forms of intervention to mean the combination of several approaches used together.
