# Supplementary material for: The Efficacy of Cognitive Intervention in Mild Cognitive Impairment (MCI): a Meta-Analysis of Outcomes on Neuropsychological Measures
Source: Neuropsychol Rev. 2017 Dec 27;27(4):440–84. doi: 10.1007/s11065-017-9363-3 (PMC5754430; doi:10.1007/s11065-017-9363-3)
Supplement: Supplementary file 16 — – Boolean Search Strategy, Terms, and Results (DOCX 23 kb) [file 11065_2017_9363_MOESM16_ESM.docx]

Table S3

*Boolean search strategy, terms, and results*

|  | **Search Terms** | **Result** | **Total Considered** |
| --- | --- | --- | --- |
| 1. | Mild cognitive impairment OR MCI OR pre-Alzheimer’s disease OR early cognitive decline OR early onset Alzheimer’s disease OR preclinical Alzheimer’s disease | 59938 |  |
|  | Limit to RCT | 5692 |  |
|  | Limit to “1995 – Current” | 5319 |  |
|  | Limit to Humans | 5318 |  |
| 2. | Intervention OR Training OR Stimulation OR Rehabilitation OR Treatment | 9155538 |  |
|  | Limit to RCT | 1358160 |  |
|  | Limit to “1995 – Current” | 1073936 |  |
|  | Limit to Humans | 1067754 |  |
| 3. | Cognition OR Thinking OR Neuropsychology | 370250 |  |
|  | Limit to RCT | 35517 |  |
|  | Limit to “1995 – Current” | 31188 |  |
|  | Limit to Humans | 31175 |  |
| 1 & 2 & 3 | Mild cognitive impairment OR MCI OR pre-Alzheimer’s disease OR early cognitive decline OR early onset Alzheimer’s disease OR preclinical Alzheimer’s disease [Limit to RCT; 1995-Current; Humans] AND Intervention OR Training OR Stimulation OR Rehabilitation OR Treatment [Limit to RCT; 1995-Current; Humans] AND Cognition OR Thinking OR Neuropsychology [Limit to RCT; 1995-Current; Humans] | 1199 |  |
|  | Excluded: Did not meet MCI criteria, absence of adequate controls, lack of adequate study design | <1102> |  |
|  | Excluded: Non-cognitive training interventions (pharmacological, physical exercise, dietary, etc.); previously reported data | <71> |  |
|  | Studies included in meta-analysis |  | 26 |
