# Supplementary material for: The Efficacy of Cognitive Intervention in Mild Cognitive Impairment (MCI): a Meta-Analysis of Outcomes on Neuropsychological Measures
Source: Neuropsychol Rev. 2017 Dec 27;27(4):440–84. doi: 10.1007/s11065-017-9363-3 (PMC5754430; doi:10.1007/s11065-017-9363-3)
Supplement: Supplementary file 17 — – List of Abbreviations (DOCX 16 kb) [file 11065_2017_9363_MOESM17_ESM.docx]

Table S4

*Summary of Abbreviations*

ACTIVE - Advanced Cognitive Training for Independent and Vital Elderly

AD8 – Alzheimer’s Disease-8 Cognitive Screen

ADAS-Cog: Alzheimer’s Disease Assessment Scale

ADL - Activities of Daily Living

ADL-PI – Activities of Daily Living – Prevention Instrument

aMCI -MD - amnestic Mild Cognitive Impairment - Multiple Domain

AMT - Attention Matrices Test

BADL - Basic Activities of Daily Living

BADS - Behavioural Assessment of the Dysexecutive Syndrome

BEM – Batterie d’Efficience Mnesique

BDI - Beck Depression Inventory

BNT - Boston Naming Test

BPSD - Behavioral and Psychotic Symptoms of Dementia

BVMT - Benton Visuospatial Memory Test

BVMT-R - Benton Visuospatial Memory Test – Revised

CAMCOG-R - Cambridge Cognitive Examination – Revised

CANTAB - Cambridge Neuropsychological Test Automated Battery

CBI - Caregiver Burden Inventory

CBQ - Caregiver Burden Questionnaire

CMMSE – Cantonese Mini-Mental Status Examination

CDAD – Chinese Disability Assessment for Dementia

CDR - Clinical Dementia Rating

CDT - Clock Drawing Test

CERAD - Consortium to Establish a Registry for Alzheimer’s Disease

CES-D - Center for Epidemiologic Studies - Depression Scale

CFQ – Cognitive Failures Questionnaire

CFT – Culture Fair Test

COWAT - Controlled Oral Word Association Test

CSDD – Cornell Scale for Depression in Dementia

CSE – Coping Self-Efficacy Scale

CSST – Corsi Supra-span Test

CTMT – Chinese Trail Making Test

CTT - Color Trail Test 1 and 2

CVFT – Category Verbal Fluency Test (Cantonese)

CVLT-II - California Verbal Learning Test – II

CWMS - Complex Working Memory Span

DAFS-R - Assessment of Functional Scale Revised

DASS-21 - Depression and Anxiety Stress Scale 21

DAQ - Divided Attention Questionnaire

D-KEFS – Delis-Kaplan Executive Function System Test Battery

DMS48 - Visual Delayed Matching-to-Sample Task

DRS-II - Dementia Rating Scale II

DS-FWD/BWD -  Digit Spin forward/backward

DVT - Digit Vigilance Test

EF – Executive Functions

EFPT – Executive Function Performance Test

EPT - Everyday Problems Test

FAQ - Functional Assessment Questionnaire

FOME - Fuld Object–Memory Evaluation

FRSSD - Functional Rating Scale for Symptoms of Dementia

FUCAS - Functional Cognitive Assessment Scale

GCS - Global Cognitive Score

GDS - Geriatric Depression Scale

GP-Cog – General Practitioner Assessment of Cognition

HADS - Hospital Anxiety and Depression Scale

HVLT-R - Hopkins Verbal Learning Test - Revised

IADL - Instrumental Activities of Daily Living

IED - Intra-/Extra-Dimensional set shifting

IGT - Iowa Gambling Task

IQCODE - Informant Questionnaire on Cognitive Decline in the Elderly

LST - Listening Span Test

MADRS - Montgomery–Åsberg Depression Rating Scale
MDB - Memory Deterioration Battery

MDRS - Mattis Dementia Rating Scale

MEC-35 - Mini Examen Cognitivo (Spanish version of MMSE)

Mem-REC - Memory Free Recall

MFQ - Memory Function Questionnaire

MMQ - Multifactorial Metamemory Questionnaire

MMAA – Medication Management Ability Assessment

MMSE - Mini-Mental State Examination

MOCA - Montreal Overall Cognitive Assessment

MWT-B - Mehrfachwahl-Wortschatz-Intelligenztest B

Nelson’s MCST - Nelson's Modified Card Sorting Test

NINDS-ADRDA - National Institute of Neurological and Communicative Disorders and Stroke - Alzheimer’s Dementia and Related Disorders Association

N-LS – Number-Letter Switching (D-KEFS)

NPE - Neuropsychiatric Evaluation

NPI - Neuropsychiatric Inventory

NPT-ES - Non-Pharmacological Therapy Experience Scale

N-S – Number Sequencing (D-KEFS)

PAL - Paired-Associates learning

PhF - Phonological Fluency

PMT – Prospective Memory Test

POMS – Profile of Mood States

PRMQ – Prospective and Retrospective Memory Questionnaire

PPT - Physical Performance Test

QoL-AD - Quality of Life in Alzheimer's Disease Scale

RAVLT - Rey Auditory Verbal Learning Test

RBANS-B/C - Repeatable Battery for the Assessment of Neuropsychological Status

RBMT - Rivermead Behavioral Memory Test

RCFT - Rey Complex Figure Test and Recognition Trial

RCT - Randomized Controlled Trial

ROCF - Rey-Osterrieth Complex Figure

RVP A - Rapid visual information processing

SDMT - Symbol Digit Modalities Test

SES - Self-Esteem Scale

SF - Semantic fluency

SKT - Short Cognitive Test

SMAF - Functional Autonomy Measurement System

SMB - Signoret’s Memory Battery

SMQ - Self-reported Motivation Questionnaire

SNA - Symbol Number Association

SOPT – Speed of Processing Training

SPMSSQ - Short Portable Mental Status Screening Questionnaire

SRT – Story Recall Test

SS – Symbol Search

STAI - State Trait Anxiety Inventory

STAI-S - State-Trait Anxiety Inventory

Stroop - Stroop Color and Word Test

TEA – Test of Everyday Attention (Elevator Test)

TICS – Telephone Interview of Cognitive Status

TMT A/B- Trail Making Test part A and B

ToL - Tower of London

ULS-8 - UCLA Loneliness Scale 8

VNT - Verbal Naming Test

VPA – Verbal Paired Associates

WAIS- III - Wechsler Adult Intelligence Scale – III

WASI - Wechsler Abbreviated Scale of Intelligence

WBS - Well-Being Scale

WCST-64 - Wisconsin Card Sorting Test 64

WTAR – Wechsler Test of Adult Reading

WMS III - Wechsler Memory Scale
