# Supplementary material for: The Efficacy of Cognitive Intervention in Mild Cognitive Impairment (MCI): a Meta-Analysis of Outcomes on Neuropsychological Measures
Source: Neuropsychol Rev. 2017 Dec 27;27(4):440–84. doi: 10.1007/s11065-017-9363-3 (PMC5754430; doi:10.1007/s11065-017-9363-3)
Supplement: Supplementary file 18 — – List of outcome measures by domain (DOCX 18 kb) [file 11065_2017_9363_MOESM18_ESM.docx]

Table S5

*Neuropsychological test instruments by cognitive domain*

| **DOMAIN** | **MEASURE** |
| --- | --- |
| **Mental Status/ General Cognitive** | |
|  | Alzheimer’s Disease Assessment Scale (ADAS-Cog) |
|  | CAMCOG-R - Cambridge Cognitive Examination - Revised |
|  | Cantonese Mini-Mental Status Examination (CMMSE) |
|  | Culture Fair Test – Scale 3 (CFT) |
|  | Dementia Rating Scale – II (DRS-2) |
|  | Mini Mental Status Exam (MMSE) |
|  | Montreal Cognitive Assessment (MOCA) |
|  | Repeatable Battery for the Assessment of Neuropsychological Status (RBANS) |
|  | Rivermead Behavioral Memory Test – I/II (RBMT I/II) |
|  |  |
| **Attention & Concentration** | |
|  | Attention/ Working Memory Composite (DS-FWB + DS-BWD) |
|  | Attentive Matrices Test (AMT; Spinnler & Tognoni (1987) |
|  | Cambridge Automated Neuropsychological Test Battery (CANTAB) – Rapid Visual Information Processing (RVP) |
|  | Categorized Working Memory Span (CWMS) |
|  | Combined Visual Detection + Digit Span Tasks (Gagnon et al. (2012) |
|  | Corsi Supra-Span Test (CSST; Orsini et al. 1987) |
|  | Digit Span – Forward (DS-FWD; De Beni et al. (2008) |
|  | Digit Span Backward (DS-BWD; De Beni et al. (2008) |
|  | Digit Span Backward (DS-BWD; Herrera et al. (2012) |
|  | Divided Attention Task – Alpha-Arithmetic & Visual Detection (Accuracy & Reaction Time; Gagnon et al., 2012) |
|  | Dot Matrix Task (Miyake et al., 2001) – Visuospatial Working Memory (VSWM) |
|  | Test of Everyday Attention (TEA) – Telephone Search |
|  | Test of Everyday Attention (TEA) – Telephone Search While Counting |
|  | Visual Detection Task – Visuospatial Attention (VDT – Gagnon et al., 2012) |
|  | Visual-Span (Lam et al., 2015) |
|  | Wechsler Adult Intelligence Scale-III (WAIS-III) – Digit Span (Total, FWD, BWD) |
|  | Wechsler Memory Scale – III (WMS-III) – Spatial Span |
|  | Wechsler Memory Scale – IV (WMS-IV) – Symbol Span |
|  |  |
| **Speed of Information Processing** | |
|  | Delis-Kaplan Executive Function System (D-KEFS) – Number Sequencing (N-S) |
|  | Symbol Digit Modalities Test (SMDT) |
|  | Trail Making Test (TMT) – Form A |
|  | Useful Field of View (UFOV) |
|  | Wechsler Adult Intelligence Scale-III (WAIS-III) – Symbol Search |
|  |  |
| **Language** |  |
|  | Boston Naming Test (BNT) |
|  | Category Verbal Fluency Test (CVFT, Lam et al., 2006) |
|  | Semantic Fluency |
|  |  |
| **Visual-Spatial** | |
|  | Repeatable Battery for the Assessment of Neuropsychological Status (RBANS) – Figure Copy |
|  | Rey-Osterrieth Complex Figure Test (RCFT) – Figure Copy |
|  | Montreal Cognitive Assessment (MOCA) – Clock Drawing |
|  |  |
| **Memory – Combined Scores** | |
|  | Memory Composite Score (HVLT & RAVLT) |
|  | Memory Composite Score (Store immediate recall, delayed recall, and recognition) |
|  | Repeatable Battery for the Assessment of Neuropsychological Status (RBANS) – Verbal and Non-Verbal Combined |
|  |  |
| **Memory – Verbal** | |
|  | 16 – Item Free and Cued Reminding Test (16-FCRT, Van der Linden et al., 2004) |
|  | California Verbal Learning Test – II (CVLT-II) |
|  | ADOS-Cog – List Learning |
|  | Batterie d’Efficience Mnesique (BEM-144; Signoret, 1991) – List Recall – Delay |
|  | List Learning – Delayed Recall |
|  | List Recall (Carretti et al., 2007) |
|  | Mini Mental Status Exam – List Recall |
|  | Montreal Cognitive Assessment (MOCA) – List Recall |
|  | Repeatable Battery for the Assessment of Neuropsychological Status (RBANS) – Story Delay |
|  | Rey Auditory Verbal Learning Test (RAVLT) |
|  | Signoret Memory Battery – Logical Memory + Word List Learning – Delay |
|  | Story Recall Test (SRT) (Choi, et al. 2011) |
|  | Wechsler Memory Scale (WMS) – Story Delay |
|  | Wechsler Memory Scale – IV (WMS-IV) - Verbal Paired Associates (VPA) I & II |
|  |  |
| **Memory – Non-Verbal** | |
|  | Benton Visual Retention Test – Revised 5^th^ Edition (BVMT-R) |
|  | Cambridge Automated Neuropsychological Test Battery (CANTAB) – Paired-Associates Learning (PAL) |
|  | Cambridge Automated Neuropsychological Test Battery (CANTAB) – Pattern Recognition Memory (PRM) |
|  | Face-Name Memory (Jean et al. 2010) |
|  | Name – Face Recall Task – Delay (Rapp et al., 2002) |
|  | Object Location – Recall (Hampstead et al., 2012) |
|  | Rey-Osterrieth Complex Figure Test (RCFT) – Figure Delay |
|  |  |
| **Executive Functions** | |
|  | Cambridge Automated Neuropsychological Test Battery (CANTAB) – Intra-/extra Dimensional Set Shifting (IED) |
|  | Cambridge Automated Neuropsychological Test Battery (CANTAB) – Spatial Working Memory (SWM) Errors & Strategy |
|  | Delis-Kaplan Executive Function System (D-KEFS) – Number-Letter Switching (N-LS) |
|  | Executive Function Composite (Animal fluency, COWAT, Stroop Color-Reading, Digit Symbol Coding) |
|  | Functional Cognitive Assessment Scale (FUCAS) – Planning |
|  | Phonemic Fluency (FAS)/ Phonological Fluency |
|  | Wechsler Adult Intelligence Scale – III (WAIS-III) – Matrices |
|  | Wechsler Adult Intelligence Scale – III (WAIS-III) – Similarities |
|  | Test of Everyday Attention (TEA) – Visual Elevator |
|  | Trail Making Test (TMT) – Form B |
|  | Reasoning Composite Score (Letter Series, Letter Sets, & Word Series combined) |
