# Supplementary material for: The Efficacy of Cognitive Intervention in Mild Cognitive Impairment (MCI): a Meta-Analysis of Outcomes on Neuropsychological Measures
Source: Neuropsychol Rev. 2017 Dec 27;27(4):440–84. doi: 10.1007/s11065-017-9363-3 (PMC5754430; doi:10.1007/s11065-017-9363-3)
Supplement: Supplementary file 19 — – Summary of overall sample characteristics and length of interventions (DOCX 23 kb) [file 11065_2017_9363_MOESM19_ESM.docx]

Table S6

*Summary of overall sample characteristics and length of intervention programs*

|  | **Mean** | **Standard Deviation** | **Minimum** | **Maximum** |
| --- | --- | --- | --- | --- |
| **Age** | 72.42 | 2.85 | 66.00 | 76.95 |
| **Edu** | 11.64 | 1.09 | 3.90 | 17.20 |
| **MMSE/MOCA** | 27.08 | 0.72 | 24.71 | 28.82 |
| **Number**  **of Participants** | 33.69 | 35.07 | 7.00 | 145.00 |
| **Duration**  **(Weeks)** | 10.49 | 7.47 | 2.00 | 24.00 |
| **Total Time**  **(Hours)** | 27.57 | 24.60 | 4.50 | 96.00 |
| **Number of Sessions**  **(Count)** | 18.29 | 13.52 | 3.00 | 48.00 |
| **Average Time/Session**  **(Hours)** | 1.49 | 0.79 | 0.33 | 4.50 |
