# Supplementary material for: The Efficacy of Cognitive Intervention in Mild Cognitive Impairment (MCI): a Meta-Analysis of Outcomes on Neuropsychological Measures
Source: Neuropsychol Rev. 2017 Dec 27;27(4):440–84. doi: 10.1007/s11065-017-9363-3 (PMC5754430; doi:10.1007/s11065-017-9363-3)
Supplement: Supplementary file 20 — – Effect sizes, confidence intervals and p-values for individual outcomes by study (assumes independence) (DOCX 42 kb) [file 11065_2017_9363_MOESM20_ESM.docx]

Table S7

*Hedges’ g effect sizes†, confidence intervals and p-values for individual outcome measures for each study*

| **Study (Year)** | **Format** | **Mode** | **Training Focus/ Intervention** | | **Outcomes** | | | **Hedges’ g**† | **Lower (95%)** | **Upper (95%)** | **p-**  **Value** | **Sig.** |
| --- | --- | --- | --- | --- | --- | --- | --- | --- | --- | --- | --- | --- |
|  |  |  |  | |  | |  |  |  |  |  |  |
|  |  |  |  | | **Domain** | | **Measure** |  |  |  |  |  |
| Balietti et al. (2016) | 10 sessions, 60 min-1x/ week | Group | Multicomponent | | Attention (WM) | | Digit Span – FWD | 1.153 | 0.651 | 1.655 | 0.000 | *** |
|  |  |  |  |  | Attention (WM) | | Corsi/CSST | 1.083 | 0.586 | 1.581 | 0.000 | *** |
|  |  |  |  |  | Attention (WM) | | Visual - Matrices | 2.172 | 1.585 | 2.759 | 0.000 | *** |
|  |  |  |  |  | Language | | Semantic Fluency | 1.031 | 0.536 | 1.525 | 0.000 | *** |
|  |  |  |  |  | Memory – Verbal | | Stories – Delay | 2.871 | 2.207 | 3.536 | 0.000 | *** |
|  |  |  |  |  | Memory – Verbal | | Stories – Imm | 3.004 | 2.323 | 3.684 | 0.000 | *** |
|  |  |  |  |  | Memory – Verbal | | Stories – Total | 3.099 | 2.407 | 3.791 | 0.000 | *** |
|  |  |  |  |  | Memory – Verbal | | Word Pairs | 2.776 | 2.123 | 3.430 | 0.000 | *** |
|  |  |  |  |  | Executive Functions | | Phonemic Fluency | 5.266 | 4.278 | 6.254 | 0.000 | *** |
|  |  |  |  |  |  | |  |  |  |  |  |  |
|  |  |  |  | |  | |  |  |  |  |  |  |
| Barban et al. (2016) | 24 sessions, 60 min session (2x/ week for 3 months) | Group based, computer administered | Multicomponent | | Memory – Verbal | | RAVLT – Delay | 0.083 | -0.299 | 0.464 | 0.671 |  |
|  |  |  |  |  | Mental Status – General Cognition | | MMSE | 0.111 | -0.271 | 0.493 | 0.570 |  |
|  |  |  |  |  |  | |  |  |  |  |  |  |
|  |  |  |  | |  | |  |  |  |  |  |  |
| Barnes et al. (2009) | 1.67 hr/day, 5days/week | Computer | Restorative | | Mental Status – General Cognition | | RBANS Total | 0.374 | -0.272 | 1.020 | 0.256 |  |
|  |  |  |  |  | Attention (WM) | | WMS-III - Spatial Span | 0.663 | 0.006 | 1.321 | 0.048 | * |
|  |  |  |  |  | Visual-Spatial Ability | | RBANS – Visuospatial Index | 0.483 | -0.089 | 1.054 | 0.098 |  |
|  |  |  |  |  | Memory (Verbal & Non-Verbal) | | RBANS – Delayed Memory (V + NV) | 0.611 | -0.044 | 1.266 | 0.068 |  |
|  |  |  |  |  |  | |  |  |  |  |  |  |
|  |  |  |  | |  | |  |  |  |  |  |  |
| Buschert et al. (2011) | 11 sessions minimum - 20 "units", 2hrs/week | Group | Multicomponent | | Mental Status – General Cognition | | ADAS-Cog | -0.911 | -1.762 | -0.060 | 0.036 | * |
|  |  |  |  |  | Mental Status – General Cognition | | MMSE | 1.685 | 0.737 | 2.634 | 0.000 | *** |
|  |  |  |  |  | Speed of Information Processing | | TMT-A | -0.327 | -1.140 | 0.486 | 0.431 |  |
|  |  |  |  |  | Memory – Verbal | | RBANS – Story Delay | 1.276 | 0.385 | 2.167 | 0.005 | ** |
|  |  |  |  |  | Executive Functions | | TMT-B | -0.954 | -1.809 | -0.099 | 0.029 | * |
|  |  |  |  |  |  | |  |  |  |  |  |  |
|  |  |  |  | |  | |  |  |  |  |  |  |
| Carretti et al. (2012) | 5, 1.5hr sessions | Individual (1:1 sessions) | Restorative | | Mental Status – General Cognition | | Cattell – CFT | 0.818 | -0.059 | 1.695 | 0.068 |  |
|  |  |  |  |  | Attention (WM) | | Digit Span – FWD | 0.203 | -0.638 | 1.045 | 0.636 |  |
|  |  |  |  |  | Attention (WM/DA) | | Digit Span – BWD | 0.346 | -0.500 | 1.193 | 0.423 |  |
|  |  |  |  |  | Attention (WM) | | CWMS – Verbal working memory | 0.989 | 0.096 | 1.883 | 0.030 | * |
|  |  |  |  |  | Attention (WM) | | Dot Matrix – Visual-spatial working memory | 0.357 | -0.490 | 1.204 | 0.409 |  |
|  |  |  |  |  | Memory – Verbal | | List Recall – Delay | 0.538 | -0.318 | 1.394 | 0.218 |  |
|  |  |  |  |  |  | |  |  |  |  |  |  |
|  |  |  |  | |  | |  |  |  |  |  |  |
| Fiatarone Singh et al. (2014) | 48, 45 min sessions (2x/week for 24 weeks) | Computer | Multicomponent | | Mental Status – General Cognition | | ADAS-Cog | -0.057 | -0.626 | 0.512 | 0.844 |  |
|  |  |  |  |  | Language | | Semantic Fluency | 0.609 | 0.027 | 1.191 | 0.040 | * |
|  |  |  |  |  | Speed of Information Processing | | SDMT | -0.272 | -0.844 | 0.299 | 0.350 |  |
|  |  |  |  |  | Memory – Verbal | | List Learning Sum (ADAS-Cog) | 0.171 | -0.399 | 0.741 | 0.557 |  |
|  |  |  |  |  | Memory – Verbal | | WMS-III – LM I (Imm) | 0.097 | -0.472 | 0.666 | 0.739 |  |
|  |  |  |  |  | Memory – Verbal | | WMS-III – LM II (Delay) | 0.296 | -0.276 | 0.868 | 0.310 |  |
|  |  |  |  |  | Memory – Non-Verbal | | BVMT-R | 0.254 | -0.317 | 0.825 | 0.383 |  |
|  |  |  |  |  | Executive Functions | | Phonemic Fluency (FAS) | 0.064 | -0.505 | 0.632 | 0.826 |  |
|  |  |  |  |  | Executive Functions | | WAIS-III Similarities | 0.559 | -0.021 | 1.138 | 0.059 |  |
|  |  |  |  |  | Executive Functions | | WAIS-III Matrices | 0.123 | -0.446 | 0.692 | 0.673 |  |
|  |  |  |  |  |  | |  |  |  |  |  |  |
|  |  |  |  | |  | |  |  |  |  |  |  |
| Finn and McDonald (2011) | 30 sessions, 11.43wks/ completion | Computer | Restorative | | Attention (WM) | | Visual Sustained Attention (RVP) | 1.056 | 0.060 | 2.052 | 0.038 | * |
|  |  |  |  |  | Memory – Non -Verbal | | Visual Delay (PAL) | 1.085 | 0.085 | 2.084 | 0.033 | * |
|  |  |  |  |  | Memory – Non -Verbal | | Visual Delay (PRM) | 0.771 | -0.193 | 1.736 | 0.117 |  |
|  |  |  |  |  | Executive Functions | | Problem Solving - SWM errors | 1.027 | 0.034 | 2.019 | 0.043 | * |
|  |  |  |  |  | Executive Functions | | Problem Solving – SWM strategy | 1.083 | 0.083 | 2.083 | 0.034 | * |
|  |  |  |  |  | Executive Functions | | Attentional Set Shifting - IED errors | 1.177 | 0.165 | 2.189 | 0.023 | * |
|  |  |  |  |  |  | |  |  |  |  |  |  |
|  |  |  |  | |  | |  |  |  |  |  |  |
| Finn and McDonald (2015) | 6 sessions over several weeks | Computer | Restorative | | Attention (WM) | | Visual WM (WMS-IV Symbol Span) | 0.404 | -0.377 | 1.185 | 0.310 |  |
|  |  |  |  |  | Speed of Information Processing | | D-KEFS - Number Seq | -0.210 | -0.985 | 0.565 | 0.596 |  |
|  |  |  |  |  | Memory – Verbal | | VPA-I | 0.151 | -0.623 | 0.924 | 0.703 |  |
|  |  |  |  |  | Memory – Verbal | | VPA-II | 0.182 | -0.592 | 0.956 | 0.645 |  |
|  |  |  |  |  | Executive Functions | | D-KEFS - N-L Switch | 0.099 | -0.675 | 0.872 | 0.803 |  |
|  |  |  |  |  |  | |  |  |  |  |  |  |
|  |  |  |  | |  | |  |  |  |  |  |  |
| Förster et al. (2011) | 26, ~2hr sessions (1x/week for 6 months) | Group | Multicomponent | | Mental Status – General Cognition | | ADAS-Cog | -0.898 | -1.825 | 0.030 | 0.058 |  |
|  |  |  |  |  | Mental Status – General Cognition | | MMSE | 1.751 | 0.701 | 2.800 | 0.001 | *** |
|  |  |  |  |  |  | |  |  |  |  |  |  |
|  |  |  |  |  |  | |  |  |  |  |  |  |
|  |  |  |  | |  | |  |  |  |  |  |  |
| Gagnon et al. (2012) | 6, 1hr sessions (3x/week for 2 weeks) | Computer | Restorative | | Attention (WM – DA) | | TEA (Telephone Search + Counting) | -0.012 | -0.784 | 0.761 | 0.976 |  |
|  |  |  |  |  | Attention (WM – Visual) | | Memory (Visual - TEA Telephone Search) | 0.644 | -0.150 | 1.437 | 0.112 |  |
|  |  |  |  |  | Attention (WM – DA) | | Alpha-Arithmetic - Accuracy | 0.827 | 0.246 | 1.407 | 0.005 | ** |
|  |  |  |  |  | Attention (WM – DA) | | Alpha-Arithmetic – Reaction Time | 0.577 | 0.008 | 1.145 | 0.047 | * |
|  |  |  |  |  | Attention (WM – DA) | | Visual Detection - Accuracy | 3.544 | 2.643 | 4.446 | 0.000 | *** |
|  |  |  |  |  | Attention (WM – DA) | | Visual Detection – Reaction Time | 0.167 | -0.391 | 0. 724 | 0.557 |  |
|  |  |  |  |  | Attention | | Cross Modality – Visual Attn & Digit Span | 0.505 | -0.280 | 1.291 | 0.208 |  |
|  |  |  |  |  | Speed of Information Processing | | TMT-A | -0.280 | -1.057 | 0.497 | 0.480 |  |
|  |  |  |  |  | Executive Functions (& DA) | | Switching - TEA (Visual Elevator) | 0.729 | -0.070 | 1.529 | 0.074 |  |
|  |  |  |  |  | Executive Functions | | TMT-B | -0.471 | -1.255 | 0.313 | 0.239 |  |
|  |  |  |  |  |  | |  |  |  |  |  |  |
|  |  |  |  | |  | |  |  |  |  |  |  |
| Giuli et al. (2016) | 10, 45 min sessions (1x/ week) | Individual (1:1) | Multicomponent | | Attention (WM) | | Digit Span – FWD | 0.210 | -0.186 | 0.606 | 0.299 |  |
|  |  |  |  |  | Attention (WM/DA) | | Digit Span – BWD | 0.789 | 0.378 | 1.199 | 0.000 | *** |
|  |  |  |  |  | Attention (WM) | | Corsi/CSST | 0.361 | -0.037 | 0.759 | 0.076 |  |
|  |  |  |  |  | Attention (WM) | | Matrices | 0.298 | -0.099 | 0.695 | 0.141 |  |
|  |  |  |  |  | Language | | Semantic Fluency | 0.134 | -0.261 | 0.530 | 0.505 |  |
|  |  |  |  |  | Memory – Verbal | | Stories – Imm | 0.543 | 0.141 | 0.945 | 0.008 | ** |
|  |  |  |  |  | Memory - Verbal | | Word Pairs | 0.812 | 0.401 | 1.223 | 0.000 | *** |
|  |  |  |  |  | Executive Functions | | Phonemic Fluency | 0.943 | 0.527 | 1.360 | 0.000 | *** |
|  |  |  |  |  |  | |  |  |  |  |  |  |
|  |  |  |  |  |  | |  |  |  |  |  |  |
|  |  |  |  | |  | |  |  |  |  |  |  |
| Greenaway et al. (2012) | 12, 1hr sessions (2x/week for 6weeks) | Group (Participant and partner-dyads) | Compensatory | | Mental Status – General Cognition | | DRS2 | 0.522 | -0.096 | 1.140 | 0.098 |  |
|  |  |  |  |  | Mental Status – General Cognition | | MMSE | 0.495 | -0.122 | 1.112 | 0.116 |  |
|  |  |  |  |  |  | |  |  |  |  |  |  |
|  |  |  |  | |  | |  |  |  |  |  |  |
| Hampstead et al. (2012) | 3, 1.0-1.5hr sessions, over 2 weeks | Individual | Compensatory | | Memory – Non-Verbal | | Visual - Percent Change | 1.312 | 0.399 | 2.225 | 0.005 | ** |
|  |  |  |  |  |  | |  |  |  |  |  |  |
|  |  |  |  |  |  | |  |  |  |  |  |  |
|  |  |  |  | |  | |  |  |  |  |  |  |
| Herrera et al. (2012) | 24, 1hr sessions (2x/week for 12weeks) | Computer | Restorative | | Attention (WM) | | Digit Span – FWD | 4.106 | 2.651 | 5.562 | 0.000 | *** |
|  |  |  |  |  | Attention (WM/DA) | | Digit Span – BWD | 1.775 | 0.815 | 2.735 | 0.000 | *** |
|  |  |  |  |  | Memory – Verbal | | MMSE – List Delay (MMSE) | 4.404 | 2.874 | 5.934 | 0.000 | *** |
|  |  |  |  |  | Memory – Verbal | | 16 Item – List Delay (16 Item) | 4.648 | 3.056 | 6.239 | 0.000 | *** |
|  |  |  |  |  | Memory – Verbal | | BEM – List Delay (BEM) | 4.639 | 3.050 | 6.229 | 0.000 | *** |
|  |  |  |  |  | Memory – Visual | | ROCFT – Figure Delay | 0.185 | -0.620 | 0.991 | 0.652 |  |
|  |  |  |  |  |  | |  |  |  |  |  |  |
|  |  |  |  | |  | |  |  |  |  |  |  |
| Jean et al. (2010a) | 6, 45min sessions (2x/week for 3weeks) | Individual | Restorative | | Mental Status – General Cognition | | MMSE | 0.495 | -0.362 | 1.353 | 0.258 |  |
|  |  |  |  |  | Mental Status – General Cognition | | DRS2 | 0.358 | -0.493 | 1.209 | 0.410 |  |
|  |  |  |  |  | Mental Status – General Cognition | | RBMT | 0.063 | -0.781 | 0.907 | 0.883 |  |
|  |  |  |  |  | Memory | | DRS2 – Memory Subscale | 0.542 | -0.318 | 1.402 | 0.217 |  |
|  |  |  |  |  | Memory – Verbal | | CVLT-II – Total Words Recalled | 0.352 | -0.499 | 1.203 | 0.417 |  |
|  |  |  |  |  | Memory – Verbal | | CVLT-II – Short Delay | 0.180 | -0.665 | 1.026 | 0.676 |  |
|  |  |  |  |  | Memory – Verbal | | CVLT-II – Long Delay | 0.005 | -0.839 | 0.849 | 0.991 |  |
|  |  |  |  |  |  | |  |  |  |  |  |  |
|  |  |  |  | |  | |  |  |  |  |  |  |
| Jeong et al. (2016) | 24, 90 min sessions (2x/week for 12 weeks) | Group | Multicomponent | | Mental Status – General Cognition | | ADAS-Cog | -0.228 | -0.572 | 0. 117 | 0.195 |  |
|  |  |  |  |  | Mental Status – General Cognition | | MMSE | 0.055 | -0.288 | 0.399 | 0.753 |  |
|  |  |  |  |  | Attention (WM/DA) | | Composite Score (FWD+BWD) | 0.106 | -0.238 | 0.449 | 0.547 |  |
|  |  |  |  |  | Memory – Verbal | | Composite Score (Imm + Delay + Recog) | 0.161 | -0.183 | 0.505 | 0.360 |  |
|  |  |  |  |  | Memory – Prospective | | Prospective | 0.343 | -0.003 | 0.689 | 0.052 |  |
|  |  |  |  |  | Executive Functions | | Composite Score | 0.065 | -0.278 | 0. 408 | 0.711 |  |
|  |  |  |  |  |  | |  |  |  |  |  |  |
|  |  |  |  | |  | |  |  |  |  |  |  |
| Lam et al. (2015) | 48, 60 min sessions (3x/ week for 4 months [Time 1], 12 months total) | Group | Multicomponent | | Mental Status – General Cognition | | ADAS-Cog | -0.117 | -0.353 | 0. 119 | 0.330 |  |
|  |  |  |  |  | Mental Status – General Cognition | | CMMSE | 0.000 | -0.236 | 0.236 | 1.000 |  |
|  |  |  |  |  | Language | | Semantic Fluency | 0.223 | -0.014 | 0.459 | 0.065 |  |
|  |  |  |  |  | Memory – Verbal | | List Learning – Delayed Recall | 0.190 | -0.046 | 0.426 | 0.115 |  |
|  |  |  |  |  |  | |  |  |  |  |  |  |
|  |  |  |  |  |  | |  |  |  |  |  |  |
|  |  |  |  | |  | |  |  |  |  |  |  |
| Mowszowski et al. (2014) | 14, 120 min sessions (2x/ week, 7 weeks) | Group & computer | Multicomponent | | Attention (WM/DA) | | WAIS-III Digit Span – Total | 0.254 | -0.376 | 0.884 | 0.429 |  |
|  |  |  |  |  | Language | | Semantic Fluency (Animals) | 0.380 | -0.252 | 1.013 | 0.239 |  |
|  |  |  |  |  | Memory – Verbal | | RAVLT – Total Words (1-5) | 0.277 | -0.354 | 0. 907 | 0.390 |  |
|  |  |  |  |  | Memory – Verbal | | RAVLT – Delay | 0.356 | -0.276 | 0.989 | 0.269 |  |
|  |  |  |  |  | Executive Functions | | Phonemic Fluency (FAS) | 0.483 | -0.154 | 1.119 | 0.137 |  |
|  |  |  |  |  | Executive Functions | | TMT-B | -0.142 | -0.770 | 0. 486 | 0.657 |  |
|  |  |  |  |  |  | |  |  |  |  |  |  |
|  |  |  |  |  |  | |  |  |  |  |  |  |
|  |  |  |  | |  | |  |  |  |  |  |  |
| Olchik et al. (2013) | 8, 90 min sessions (2x/ week, 4 weeks) | Group | Multicomponent | | Mental Status – General Cognition | | RBMT – Screening Score | 0.132 | -0.567 | 0.830 | 0.712 |  |
|  |  |  |  |  | Mental Status – General Cognition | | RBMT – Story – Imm | 0.069 | -0.629 | 0. 768 | 0.845 |  |
|  |  |  |  |  | Mental Status – General Cognition | | RBMT – Story – Delay | 0.422 | -0.284 | 1.128 | 0.241 |  |
|  |  |  |  |  | Language | | Semantic Fluency | 0.688 | -0.031 | 1.407 | 0.061 |  |
|  |  |  |  |  | Memory – Verbal | | RAVLT – Total words | 0.453 | -0.254 | 1.161 | 0.209 |  |
|  |  |  |  |  | Memory – Verbal | | RAVLT – Imm | 0.449 | -0.258 | 1.156 | 0.213 |  |
|  |  |  |  |  | Memory – Verbal | | RAVLT – Delay | 0.487 | -0.221 | 1.196 | 0.178 |  |
|  |  |  |  |  | Executive Functions | | Phonemic Fluency (FAS) | 0.270 | -0.431 | 0.971 | 0.450 |  |
|  |  |  |  |  |  | |  |  |  |  |  |  |
|  |  |  |  | |  | |  |  |  |  |  |  |
| Polito et al. (2015) | 10, 100 min sessions (2x/ week, 5 weeks) | Group | Multicomponent | | Mental Status – General Cognition | | MMSE | 0.020 | -0.560 | 0.600 | 0.946 |  |
|  |  |  |  |  | Mental Status – General Cognition | | MOCA | 0.139 | -0.442 | 0.720 | 0.639 |  |
|  |  |  |  |  | Attention (WM) | | CSST | 0.031 | -0.550 | 0.611 | 0.918 |  |
|  |  |  |  |  |  | |  |  |  |  |  |  |
|  |  |  |  | |  | |  |  |  |  |  |  |
| Rapp et al. (2002) | 6, 2hr sessions (1x/week for 6weeks) | Group | Multicomponent | | Memory – Verbal | | Shopping List – Imm | 0.144 | -0.791 | 1.079 | 0.762 |  |
|  |  |  |  |  | Memory – Verbal | | Shopping List – Delay | 0.534 | -0.418 | 1.486 | 0.272 |  |
|  |  |  |  |  | Memory – Verbal | | Word List – Imm | 1.162 | 0.145 | 2.179 | 0.025 | * |
|  |  |  |  |  | Memory – Verbal | | Word List – Delay | 0.909 | -0.076 | 1.895 | 0.071 |  |
|  |  |  |  |  | Memory – Verbal | | WMS Story – Imm | 0.030 | -0.904 | 0.964 | 0.949 |  |
|  |  |  |  |  | Memory – Verbal | | WMS Story – Delay | 0.518 | -0.433 | 1.469 | 0.285 |  |
|  |  |  |  |  | Memory – Non-Verbal | | Name-Face – Imm | 0.611 | -0.346 | 1.569 | 0.211 |  |
|  |  |  |  |  | Memory – Non-Verbal | | Name-Face – Delay | 0.619 | -0.339 | 1.577 | 0.205 |  |
|  |  |  |  |  |  | |  |  |  |  |  |  |
|  |  |  |  | |  | |  |  |  |  |  |  |
| Rojas et al. (2013) | 52, 2hr sessions (2x/week for 6 months) | Group | Multicomponent | | Mental Status – General Cognition | | MMSE | 0.926 | 0.191 | 1.661 | 0.014 | * |
|  |  |  |  |  | Language | | BNT | 0.441 | -0.264 | 1.146 | 0.220 |  |
|  |  |  |  |  | Language | | Semantic Fluency | 1.493 | 0.701 | 2.286 | 0.000 | *** |
|  |  |  |  |  | Memory – Verbal | | Memory - List Delay | 0.944 | 0.208 | 1.680 | 0.012 | * |
|  |  |  |  |  | Executive Functions | | Phonemic Fluency (FAS) | 0.663 | -0.053 | 1.380 | 0.069 |  |
|  |  |  |  |  |  | |  |  |  |  |  |  |
|  |  |  |  |  |  | |  |  |  |  |  |  |
|  |  |  |  | |  | |  |  |  |  |  |  |
| Schmitter-Edgecombe & Dyck (2014) | 20, 2hr sessions, (2x/week for 10 weeks) | Group | Multicomponent | | Mental Status – General Cognition | | RBMT-II | 0.336 | -0.236 | 0.908 | 0.250 |  |
|  |  |  |  |  | Memory (Verbal & Non-Verbal) | | Memory - Imm (RBANS V + NV) | 0.194 | -0.375 | 0.764 | 0.504 |  |
|  |  |  |  |  | Memory (Verbal & Non-Verbal) | | Memory - Delay (RBANS V + NV) | 0.293 | -0.278 | 0.865 | 0.314 |  |
|  |  |  |  |  |  | |  |  |  |  |  |  |
|  |  |  |  |  |  | |  |  |  |  |  |  |
|  |  |  |  | |  | |  |  |  |  |  |  |
| Tsolaki et al. (2011) | 60, 1.5hr sessions, (3x/week for 5 months) | Group | Multicomponent | | Mental Status – General Cognition | | MMSE | 0.388 | 0.086 | 0.689 | 0.012 | * |
|  |  |  |  |  | Mental Status – General Cognition | | MOCA | 0.584 | 0.279 | 0.890 | 0.000 | *** |
|  |  |  |  |  | Memory – Verbal | | MOCA – List Delay | 0.482 | 0.179 | 0.786 | 0.002 | ** |
|  |  |  |  |  | Visual – Spatial | | MOCA – Clock Drawing | 0.367 | 0.065 | 0.669 | 0.017 | * |
|  |  |  |  |  | Visual – Spatial | | ROCFT – Copy | 0.381 | 0.079 | 0.683 | 0.013 | * |
|  |  |  |  |  | Executive Functions | | FUCAS Planning | 0.343 | 0.042 | 0.645 | 0.026 | * |
|  |  |  |  |  |  | |  |  |  |  |  |  |
|  |  |  |  | |  | |  |  |  |  |  |  |
| Valdes et al. (2012) | 10 sessions, 1.0 hr/ group sessions (5-week duration) | Group | Restorative | | Speed of Information Processing | | UFOV | -1.565 | -1.887 | -0.243 | 0.000 | *** |
|  |  |  |  |  |  | |  |  |  |  |  |  |
|  |  |  |  |  |  | |  |  |  |  |  |  |
|  |  |  |  | |  | |  |  |  |  |  |  |
| Vidovich et al. (2015) | 10, 1.5hr sessions, (2x/week for 5 weeks) | Group | Multicomponent | | Mental Status – General Cognition | | CAMCOG-R | 0.247 | -0.066 | 0.561 | 0.122 |  |
|  |  |  |  |  | Attention (WM) | | Digit Span – FWD | 0.196 | -0.118 | 0.510 | 0.222 |  |
|  |  |  |  |  | Attention (WM/DA) | | Digit Span – BWD | 0.088 | -0.225 | 0.402 | 0.580 |  |
|  |  |  |  |  | Speed of Information Processing | | WAIS-III – Symbol Search (Items Comp) | 0.361 | 0.042 | 0.680 | 0.026 | * |
|  |  |  |  |  | Speed of Information Processing | | TMT-A | -0.087 | -0.400 | 0.227 | 0.588 |  |
|  |  |  |  |  | Memory – Verbal | | CVLT-II – Total | 0.212 | -0.102 | 0.526 | 0.186 |  |
|  |  |  |  |  | Memory – Verbal | | CVLT-II – Short Delay | 0.358 | 0.042 | 0.674 | 0.026 | * |
|  |  |  |  |  | Memory – Verbal | | CVLT-II – Long Delay | 0.291 | -0.024 | 0.606 | 0.070 |  |
|  |  |  |  |  | Executive Functions | | Phonemic Fluency (FAS) | 0.014 | -0.299 | 0.327 | 0.931 |  |
|  |  |  |  |  | Executive Functions | | TMT-B | -0.220 | -0.537 | 0.097 | 0.174 |  |
|  |  |  |  |  |  | |  |  |  |  |  |  |
|  |  |  |  | |  | |  |  |  |  |  |  |
|  | | | |  | | †Assumes independence | | | | | | |
|  | | | |  | | *p < 0.05 | | | | | | |
|  | | | |  | | **p < 0.01 | | | | | | |
|  | | | |  | | ***p < 0.001 | | | | | | |
