# Supplementary material for: The Efficacy of Cognitive Intervention in Mild Cognitive Impairment (MCI): a Meta-Analysis of Outcomes on Neuropsychological Measures
Source: Neuropsychol Rev. 2017 Dec 27;27(4):440–84. doi: 10.1007/s11065-017-9363-3 (PMC5754430; doi:10.1007/s11065-017-9363-3)
Supplement: Supplementary file 21 — – Sensitivity Analysis: Effect sizes and measures of dispersion when combining outcomes at various levels of correlation between test instruments (DOCX 17 kb) [file 11065_2017_9363_MOESM21_ESM.docx]

Table S8

*Sensitivity Analysis: Effect sizes and measures of dispersion when combining outcomes at various levels of correlation between test instruments (within-study)*

| **Combined Outcomes** | | **Hedges’**  **g** | **Standard Error** | **Variance** | **Actual/ Observed**  **Intervals** | | ***Z* -Value** | ***p -*Value** | **τ^2^** | **τ** |
| --- | --- | --- | --- | --- | --- | --- | --- | --- | --- | --- |
|  | **Correlation Assumed** |  |  |  | **Lower**  **(95%)** | **Upper**  **(95%)** |  |  |  |  |
|  |  |  |  |  |  |  |  |  |  |  |
|  | **All Interventions – All Outcomes** | | |  |  |  |  |  |  |  |
|  | *r* = 0.00 | 0.492 | 0.122 | 0.015 | 0.253 | 0.732 | 4.028 | 0.000 | 0.356 | 0.597 |
|  | *r* = 0.20 | 0.479 | 0.128 | 0.016 | 0.229 | 0.729 | 3.756 | 0.000 | 0.373 | 0.611 |
|  | *r* = 0.40 | 0.470 | 0.134 | 0.018 | 0.207 | 0.733 | 3.500 | 0.000 | 0.401 | 0.633 |
|  | *r* = 0.60 | 0.463 | 0.141 | 0.020 | 0.187 | 0.739 | 3.288 | 0.001 | 0.430 | 0.656 |
|  | *r* = 0.80 | 0.457 | 0.147 | 0.022 | 0.169 | 0.745 | 3.112 | 0.002 | 0.459 | 0.678 |
|  | *r* = 1.00 | 0.452 | 0.153 | 0.023 | 0.153 | 0.752 | 2.964 | 0.003 | 0.488 | 0.698 |
|  |  |  |  |  |  |  |  |  |  |  |
|  | **Multicomponent Training – All Outcomes** | | |  |  |  |  |  |  |  |
|  | *r* = 0.00 | 0.440 | 0.137 | 0.019 | 0.171 | 0.709 | 3.202 | 0.001 | 0.280 | 0.529 |
|  | *r* = 0.20 | 0.433 | 0.127 | 0.016 | 0.184 | 0.682 | 3.402 | 0.001 | 0.224 | 0.473 |
|  | *r* = 0.40 | 0.425 | 0.124 | 0.015 | 0.182 | 0.668 | 3.431 | 0.001 | 0.198 | 0.445 |
|  | *r* = 0.60 | 0.417 | 0.123 | 0.015 | 0.177 | 0.658 | 3.407 | 0.001 | 0.180 | 0.424 |
|  | *r* = 0.80 | 0.410 | 0.122 | 0.015 | 0.171 | 0.648 | 3.364 | 0.001 | 0.166 | 0.407 |
|  | *r* = 1.00 | 0.402 | 0.121 | 0.015 | 0.164 | 0.639 | 3.312 | 0.001 | 0.153 | 0.392 |
|  |  |  |  |  |  |  |  |  |  |  |
